# Nonparametric Regression for 3D Point Cloud Learning

**Published:** 2024

**Authors:** Xinyi Li, Shan Yu, Yueying Wang, Guannan Wang, Li Wang, Ming-Jun Lai, Alzheimer’s Disease Neuroimaging Initiative

**Affiliations:** School of Mathematical and Statistical Sciences, Clemson University Clemson, SC 29634, USA; Department of Statistics, University of Virginia Charlottesville, VA 22904, USA; Amazon.com, Inc. Seattle, WA 98121, USA; Department of Mathematics, College of William & Mary Williamsburg, VA 23185, USA; Department of Statistics, George Mason University Fairfax, VA 22030, USA; Department of Mathematics, University of Georgia Athens, GA 30602, USA

**Keywords:** 3D pattern recognization, complex domain, penalized splines, triangulation, trivariate splines

## Abstract

In recent years, there has been an exponentially increased amount of point clouds collected with irregular shapes in various areas. Motivated by the importance of solid modeling for point clouds, we develop a novel and efficient smoothing tool based on multivariate splines over the triangulation to extract the underlying signal and build up a 3D solid model from the point cloud. The proposed method can denoise or deblur the point cloud effectively, provide a multi-resolution reconstruction of the actual signal, and handle sparse and irregularly distributed point clouds to recover the underlying trajectory. In addition, our method provides a natural way of numerosity data reduction. We establish the theoretical guarantees of the proposed method, including the convergence rate and asymptotic normality of the estimator, and show that the convergence rate achieves optimal nonparametric convergence. We also introduce a bootstrap method to quantify the uncertainty of the estimators. Through extensive simulation studies and a real data example, we demonstrate the superiority of the proposed method over traditional smoothing methods in terms of estimation accuracy and efficiency of data reduction.

## 1 Introduction

Recent advances in computer and information technology have dramatically boosted the availability of three-dimensional (3D) point clouds in many fields, such as geography, environmental science, computer graphics, engineering, economics, and medical imaging. These point clouds are usually collections of enormous measurements in space defined by a given coordinates system. Sometimes, these point clouds are rendered and inspected directly; examples can be found in Rusinkiewicz and Levoy (2000). More often, the point clouds are converted to polygon mesh or triangle mesh models; for example, 3D reconstruction is widely used for automated driving vehicles (Lee et al., 2016; Shao, 2021). Many efforts have been devoted to investigating the converting technique, and a survey of surface reconstruction from point clouds can be found in Berger et al. (2017).

These 3D data typically contain more information than the shape of an object, as they represent some meaningful values in real life. For example, healthcare professionals can use brain activity levels contained in 3D neuroimages to access new angles, resolutions, and details to better understand tissue. Another example is using cloud-based 3D scans to explore the location and access the grade and type of ore in the mining industry. Regardless of the potential of these applications, limited research has been developed on identifying underlying signals from 3D solid objects, as there are many challenges in achieving this goal.

**Figure 1:**
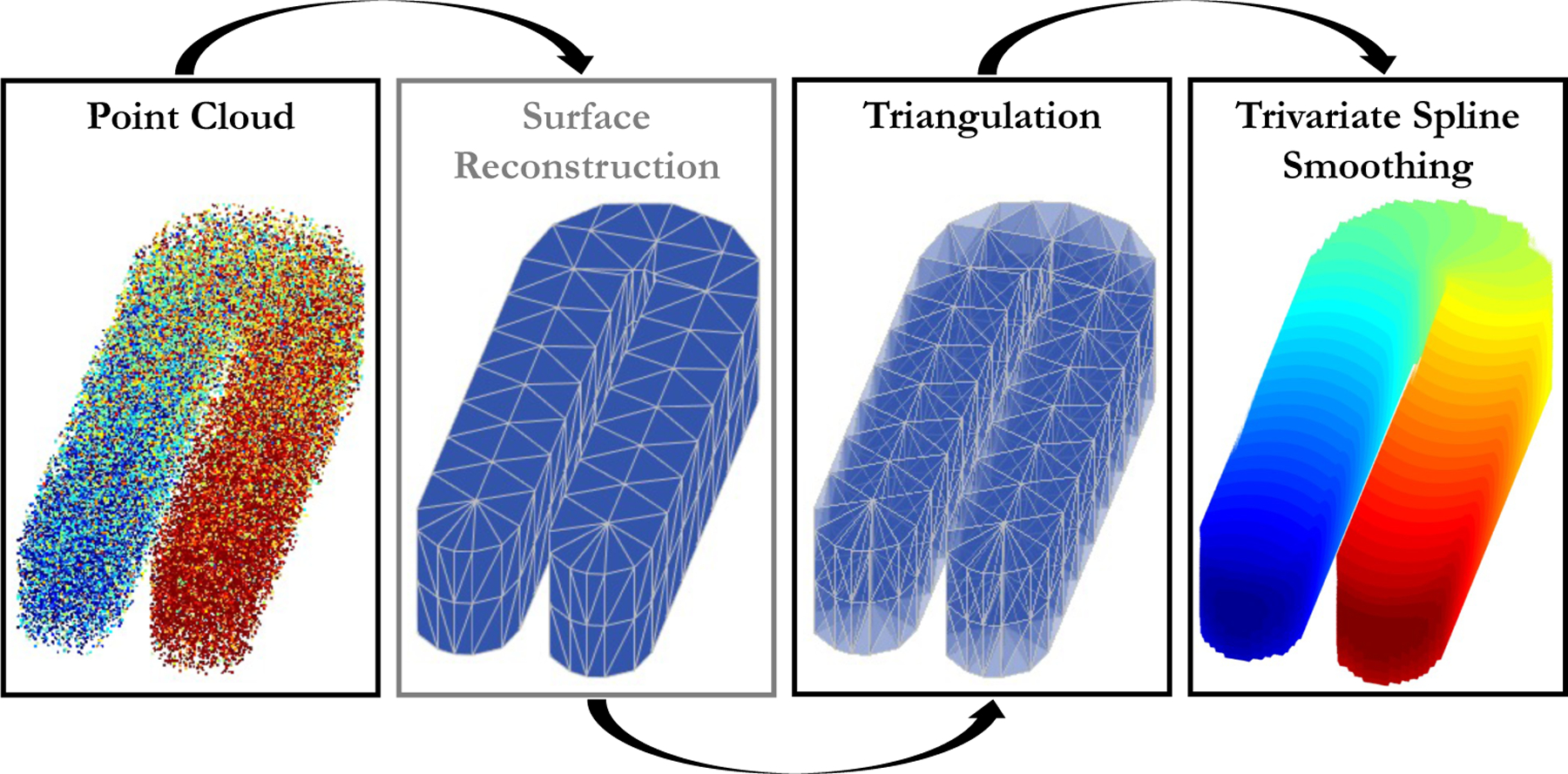
A flowchart demonstrating the overall procedure of TPST method analyzing a 3D point cloud.

**Figure 2:**
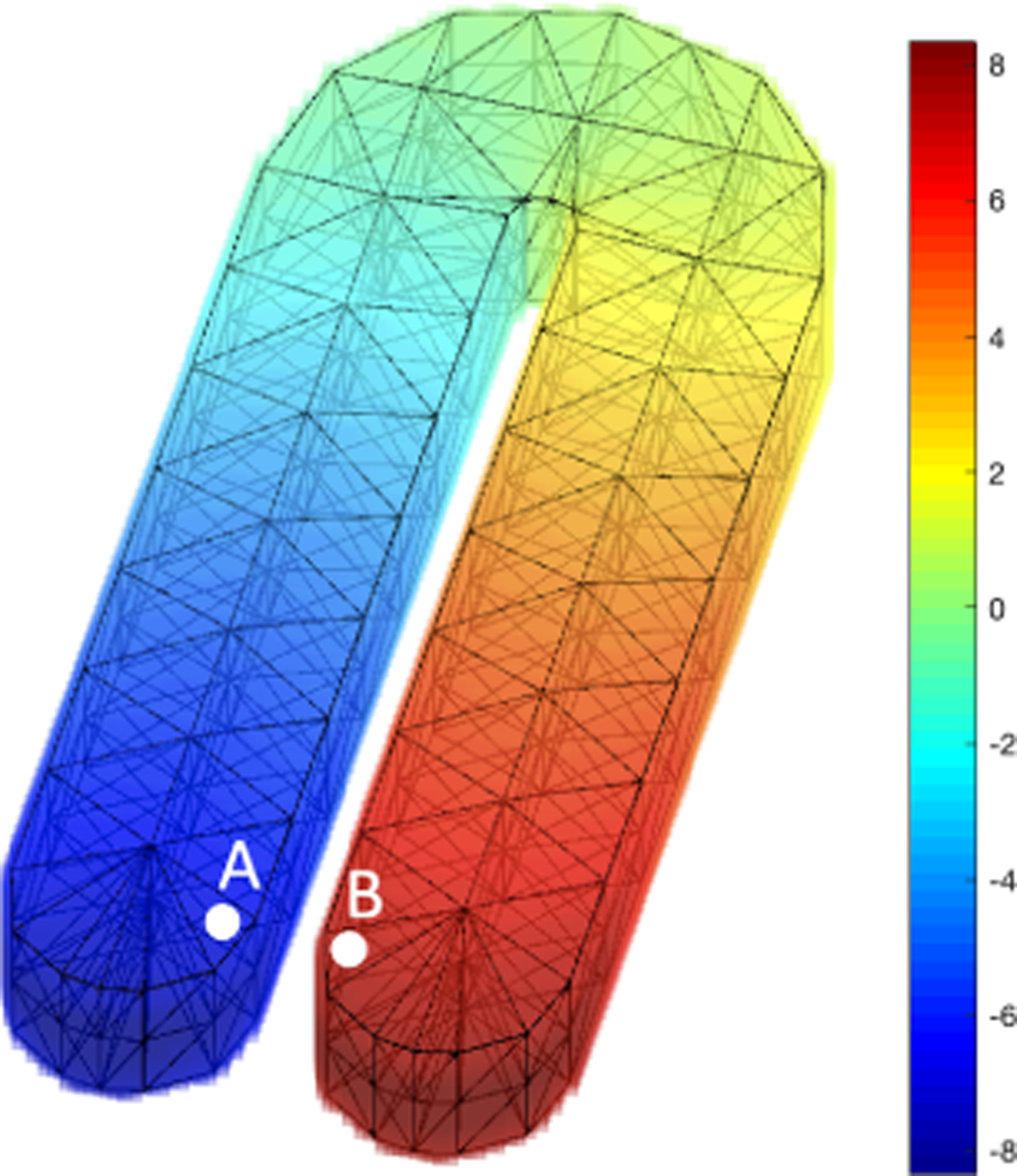
A illustration of “leakage” problem.

First of all, as shown in the point cloud inputs in Figure 1, the shape of the point clouds is usually irregular. To extract the underlying signal over the irregular-shaped 3D solid object, many conventional methods, such as kernel smoothing (Zhu et al., 2014), tensor product smoothing (Reiss and Ogden, 2010), thin plate spline smoothing (Duchon, 1976; Yue and Speckman, 2010), and wavelet smoothing (Morris and Carroll, 2006), suffer from the problem of “leakage”, which is referred as the inaccurate signal reconstruction across the complex domains; see the discussions in Ramsay (2002), Wang and Ranalli (2007), and Kim and Wang (2021) for more details. For example, as shown in the pillar of the horseshoe domain in Figure 2, although the Euclidean distance between points A and B is very short, erroneously borrowing information from point B when estimating point A will lead to unsatisfactory results. Several techniques have been developed in the last two decades to tackle the “leakage” problem for two-dimensional (2D) smoothing, such as spline smoothing (Awanou et al., 2006; Wang and Ranalli, 2007; Lai and Wang, 2013; Wilhelm et al., 2016), finite element analysis (Lindgren et al., 2011; Arnone et al., 2022), and kernel smoothing (Guo et al., 2010). For 3D images, Chung et al. (2018) applied the discrete heat kernel smoothing, and for irregular-shaped 3D objects, Huo et al. (2020) considered the finite element method.

Second, the information comprised in the 3D point clouds usually contains unwanted noise that can obscure the features of the underlying signal of interest. Consequently, denoising is a crucial step in the modeling process. Various smoothing methods have been adopted for denoising in many 2D studies (Goldsmith et al., 2014; Kang, 2020). However, the unique characteristics of 3D point clouds present significant challenges in denoising along with analysis (Wong et al., 2016).

Third, due to the nature of the devices for generating point cloud data, raw point cloud data is often sparse, uneven, or even partially missing. Therefore, accurately extracting the underlying signal from these point clouds becomes incredibly challenging. Consequently, it is necessary to preprocess the raw data to generate complete, dense, and uniform point cloud data.

Last but not least, as aforementioned, the size of the point clouds is usually enormous, and thus, makes it challenging in both storage and the corresponding data analysis. For example, a 3D image produced from a standard positron emission tomography (PET) brain scan contains over half a million voxels, which leaves data processing and analysis computing intensive tasks.

To address the prevalent challenges in converting a 3D irregular-shaped point cloud to a solid model and accurately estimating the underlying function, we propose a novel and efficient smoothing method based on trivariate penalized spline over triangulations (TPST) with a directional derivative-based penalty function. Figure 1 demonstrates the flowchart of the overall procedure of the TPST method for analyzing a 3D point cloud. The proposed TPST smoothing method has several appealing properties for handling data collected from a point cloud.

One of the main advantages is its ability to effectively solve the problem of “leakage” across complex domains. By borrowing the information from neighboring tetrahedra, the TPST method is able to accurately denoise or deblur the collected data while preserving inherent geometric features or spatial structures. This makes it particularly useful for analyzing irregularly shaped 3D point clouds. Similar to P-splines of Eilers and Marx (1996), TPST uses compactly supported basis functions and a sparse penalty, which is computationally efficient for smoothing unevenly distributed data, such as sparse and irregular 3D point cloud data. By using an appropriate partition, the method can reconstruct or recover the underlying function from both global and local missing data, and provide a multi-resolution reconstruction of the actual signal.

In addition, the proposed TPST has the potential to be used as a numerosity reduction method (Han et al., 2011). With a smooth underlying signal, the smaller set of spline coefficients could well represent and thus significantly reduce the amount of original data. Once the spline basis functions are determined, we can use the coefficients to recover the underlying signal of the point clouds. For example, as demonstrated in Section 6, a point cloud of size 50,000 can be represented by a vector of 323 spline coefficients. Let’s take the biomedical image for another example, where the proposed method can reduce an image point cloud of 510,340 voxels to a vector of 4,856 coefficients, with a peak signal-to-noise ratio reaching 30.76. See Table 2 in a later section for details. This can greatly alleviate the storage and computational challenges associated with large point clouds.

Furthermore, the proposed TPST is computationally efficient in dealing with point clouds of large size. As shown in Section 3, it is very easy to set up the spline basis functions and penalties of TPST. Similar to many other types of spline smoothing (Wang and Yang, 2009; Ma, 2012; Wang et al., 2020a,b; Liu and Zhao, 2021), TPST is a global estimation method with an explicit model expression, and consequently, it only requires solving a single linear system to obtain the estimate. Thus, TPST could reduce the computational complexity and make it suitable for handling large point clouds.

In this article, we also investigate the theoretical properties of the proposed smoothing method. To be more specific, we establish the convergence rate of the proposed TPST estimator, which is determined by the fineness of the triangulation, the trivariate spline degree and smoothness, penalty parameter, and smoothness of the unknown function. When the penalty parameter is zero, the rate achieves the optimal nonparametric convergence rate. We further derive the asymptotic normality for the TPST estimator.

When working with point clouds, uncertainty quantification is essential after the estimate, as it provides an assessment of the reliability and robustness of the estimators, based on which results one could make more accurate conclusions. The aforementioned asymptotic normality of the proposed TPST estimator could thus be used to establish pointwise confidence intervals in theory. In practice, however, it can be difficult to derive the exact form of the standard error due to the characteristics of the trivariate spline basis functions. As an alternative, we propose using a wild bootstrap method to estimate the standard error. This method is particularly useful for 3D point clouds that may have varying levels of noise or uncertainty as it can handle the heterogeneity effectively in data. Our simulation results show that the bootstrap standard error is very close to the true standard errors, providing a reliable method for quantifying the uncertainty of the estimators.

The rest of the paper is structured as follows. In Section 2, we give an overview of the triangulations and the methods for constructing the triangulations from a 3D point cloud. Once a triangulation is obtained, we can construct the spline space over this triangulation. We introduce the penalized spline estimators in Section 3. In Section 4, we present the theoretical results. In Section 5, we discuss implementation details for the proposed TPST method, including the creation of the penalty matrix, selection of the penalty parameter, choice of triangulation, and evaluations of uncertainties. We then demonstrate the performance of the proposed smoothers on several simulation examples and a real data example in Section 6. Finally, this paper is concluded, and future work is outlined in Section 7. We put all the theoretical details and more detailed introductions in the supplementary material.

## 2 Trivariate Splines over Triangulations and Basic Properties

In this section, we provide a basic framework for triangulations and trivariate splines on those triangulations. Please refer to Appendix A for more detailed introductions.

### 2.1 Triangulations

**Figure 3:**
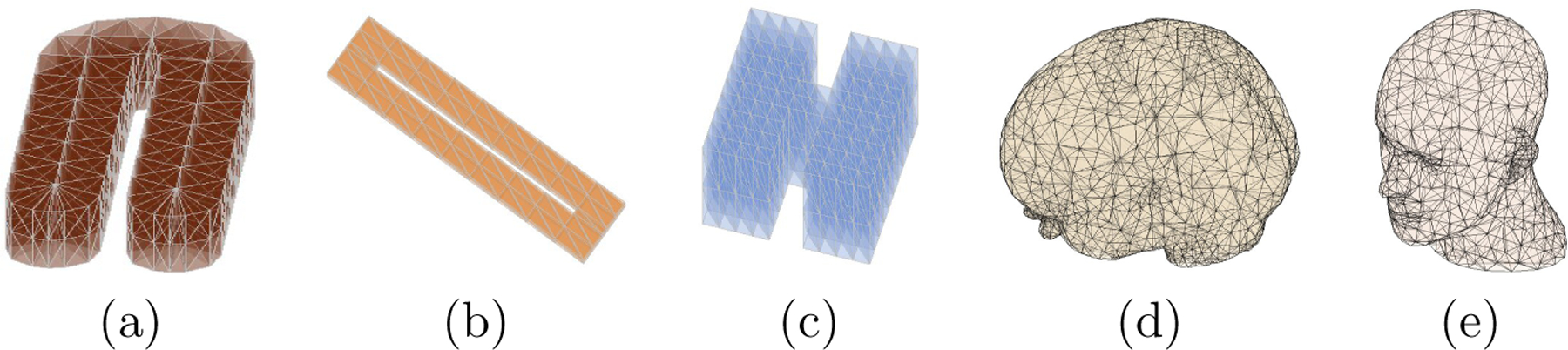
Examples of 3D point clouds and corresponding triangulation.

**Figure 4:**
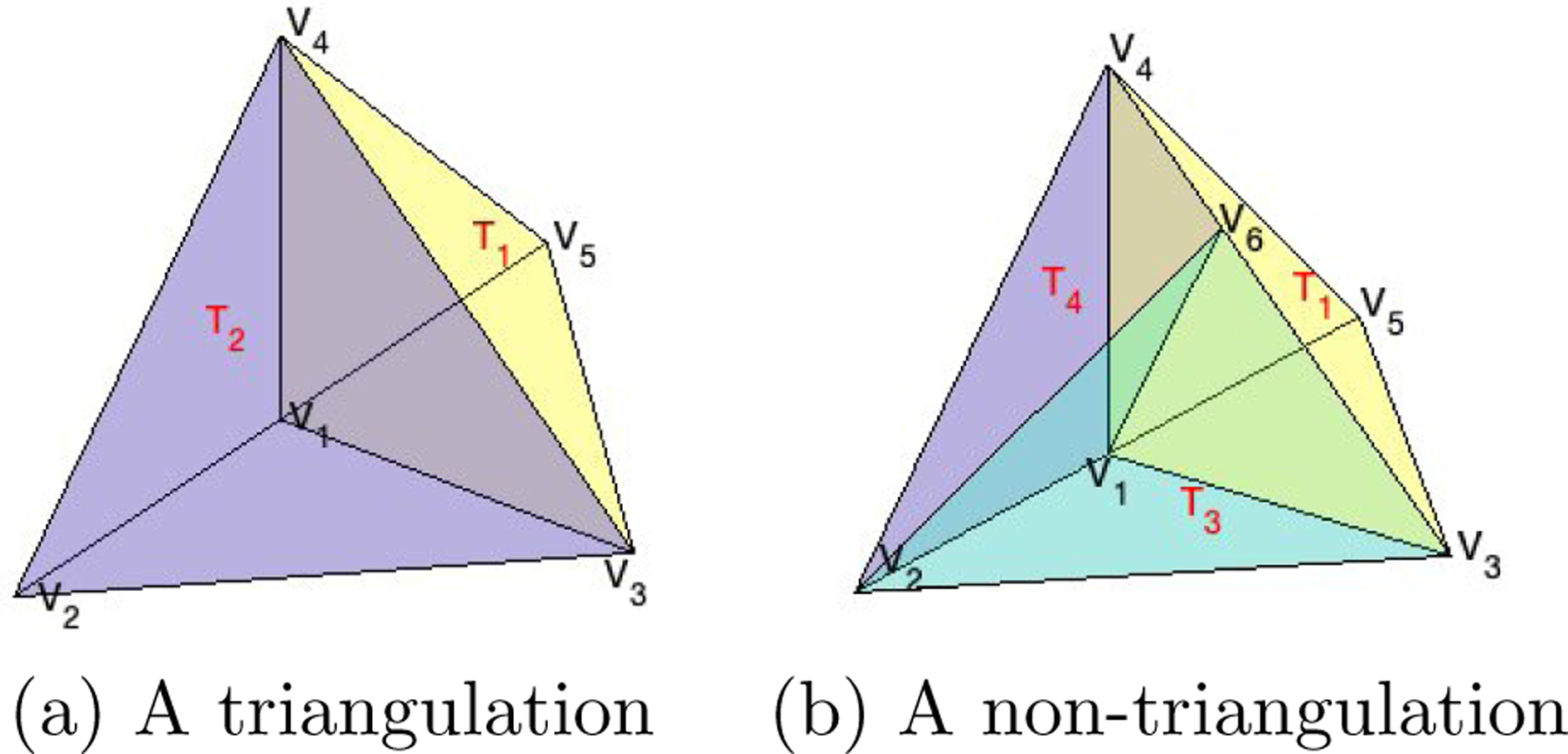
(a) and (b) provide an example and a counterexample of triangulation.

We assume there exists a tight polyhedral domain, Ω, that contains all the data locations of a point cloud. In the theoretical development below, we consider the Ω is fixed and given. Additionally, we take into account that the domain may have one or multiple holes that do not contain any observations, as seen in the 3D domain of Figure 3 (b). Triangulations are very popular in approximating the domain Ω. In the following, we use T to denote a tetrahedron, that is, a convex hull of four noncoplanar points in $R^{3}$. Then, a collection $\Delta =\left\{T_{1},\ldots ,T_{N}\right\}$ of N tetrahedra is called a *triangulation* of $\Omega =\cup_{h=1}^{N}T_{h}$, provided that any pair of tetrahedra in Δ intersect at most at a common vertex, along a common edge, or along a common triangular face. An example of triangulation $\Delta =\left\{T_{1}=\left\langle v_{5},v_{1},v_{4},v_{3}\right\rangle ,T_{2}=\right.\;\left.\left\langle v_{2},v_{1},v_{3},v_{4}\right\rangle \right\}$ of the domain $\Omega =\left\langle v_{2},v_{5},v_{3},v_{4}\right\rangle$ is illustrated in Figure 4 (a), where $v_{ı},\;ı=1,\ldots ,5$, are the vertices of the tetrahedra. In contrast, as illustrated in Figure 4 (b) for tetrahedra $T_{1},\;T_{3}=\left\langle v_{2},v_{1},v_{3},v_{6}\right\rangle$ and $T_{4}=\left\langle v_{2},v_{1},v_{6},v_{4}\right\rangle ,\left\{T_{1},T_{3},T_{4}\right\}$ does not form a triangulation of Ω, because of the intersections between the pairs of tetrahedra $\left(T_{1},T_{3}\right)$ and $\left(T_{1},T_{4}\right)$.

**Figure 5:**
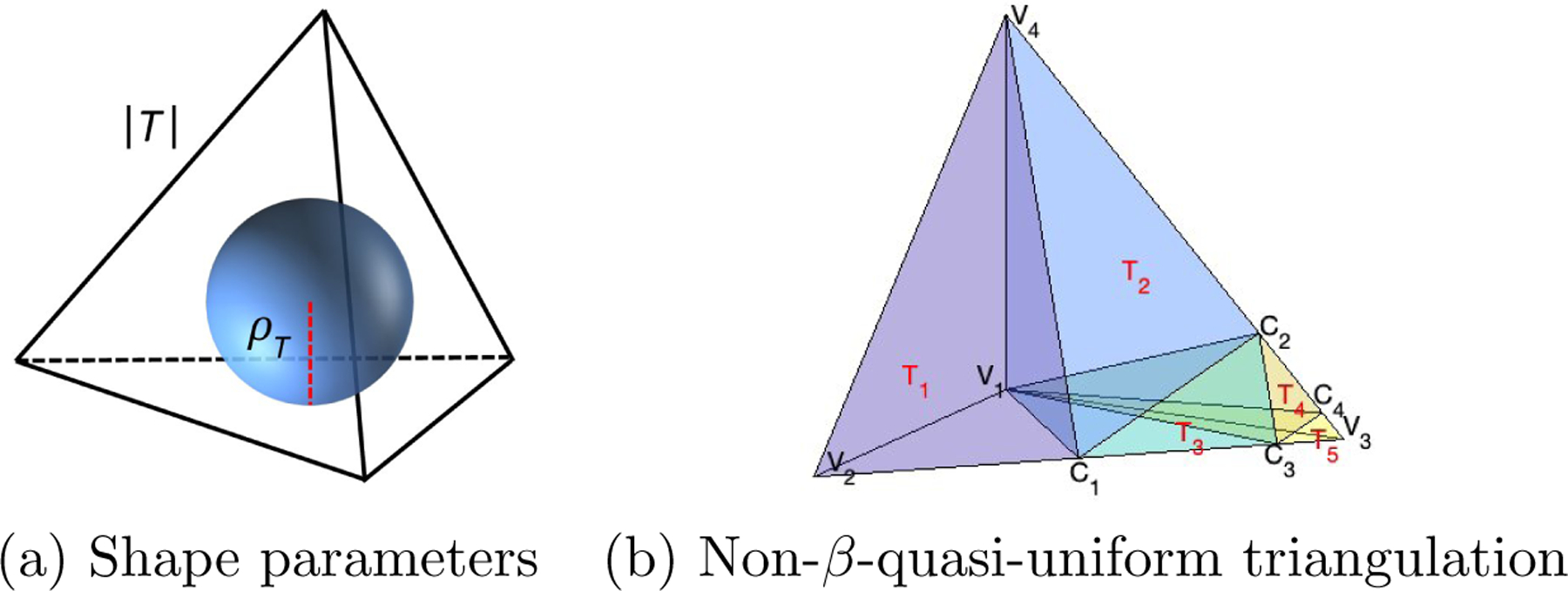
(a) shows an illustration of the shape parameters of a tetrahedron; (b) shows an example of a non-β-quasi-uniform triangulation.

Furthermore, let |T| be the length of the longest edge of T, and $\varrho_{T}$ be the radius of the largest ball that can be inscribed in T, then the ratio $\beta_{T}:=|T|/\varrho_{T}$ is the *shape parameter* of T; see an illustration example in Figure 5 (a). In general, the shape parameter $\beta_{T}$ describes the shape of T, and the larger $\beta_{T}$ is, the flatter the tetrahedron T becomes. If T is a regular tetrahedron whose six edges are all of the same lengths, then $\beta_{T}=2\sqrt{6}$, and for any other tetrahedron, $\beta_{T}>2\sqrt{6}$. Based on this shape parameter, we can define that a triangulation Δ is β-*quasi-uniform* if there is a positive value β such that Δ satisfies

(1) $$|\Delta |/\varrho_{T}\leq \beta <\infty ,\;\text{for all }T\in \Delta ,$$

where |Δ|:=max{|T|,T∈Δ} is referred as the *size* of Δ, that is, the length of the longest edge of Δ. Let N be the number of the tetrahedra in the polygonal domain Ω. From Equation 1, we have for a β-quasi-uniform partition, $N\leq {\left(4\pi |\Delta {|}^{3}\right)}^{-1}3V_{\Omega }\beta^{3}$, where $V_{\Omega }$ denotes the volume of Ω.

To illustrate the definition of quasi-uniform partition, we show an example of non-β-quasi-uniform triangulation in Figure 5 (b),

$$\Delta =\left\{T_{1}=\right.\left\langle v_{2},v_{1},c_{1},v_{4}\right\rangle ,T_{2}=\left\langle v_{1},c_{2},c_{1},v_{4}\right\rangle ,T_{3}=\left\langle c_{1},v_{1},c_{3},c_{2}\right\rangle ,\left.T_{4}=\left\langle c_{3},v_{1},c_{4},c_{2}\right\rangle ,T_{5}=\left\langle c_{3},v_{1},v_{3},c_{4}\right\rangle ,\ldots \right\}.$$

where the edges $\left\langle v_{4},c_{1}\right\rangle$ and $\left\langle c_{2},c_{3}\right\rangle$ are perpendicular to the edge $\left\langle v_{2},v_{3}\right\rangle$, and $\left\langle c_{1},c_{2}\right\rangle$ and $\left\langle c_{3},c_{4}\right\rangle$ are perpendicular to $\left\langle v_{3},v_{4}\right\rangle$.

### 2.2 Point Clouds to Triangulations

We now describe how to construct a triangulation from a 3D point cloud. The first step is to determine the domain of the point cloud, which is often given as a polygonal domain Ω, a geometric region defined by a set of polygons. This can be used to represent a wide range of objects, such as buildings, terrain, and objects in medical imaging. If the domain of the point cloud is not known, we can use surface reconstruction techniques, such as triangular meshes, to find a tight polygonal domain that encases the point cloud. As Ω has piecewise linear boundary faces, we have a normal direction perpendicular to each planar face of Ω, which can be used to determine whether a point is inside or outside of Ω. Then, we use this normal direction information of all boundary faces and the observed data locations to partition Ω into a collection of tetrahedra. Many mesh generation algorithms for 3D domains available in various software packages and toolboxes, such as the MATLAB functions “delaunay” and “distmesh/distmeshnd” (Persson and Strang, 2004), the C++ library “CGAL” (Jamin et al., 2015; Project, 2020), the “TetGen” (Si, 2015), and “iso2mesh” (Fang and Boas, 2009) built on “CGAL”. In this article, we consider a triangulation, Δ, with vertices containing the partial or whole set of observed data locations to achieve interpolation at the given data locations. This type of triangulation can be constructed using the constrained Delaunay triangulation method, as described in Shewchuk (1998). Based on a similar method proposed in Xu (2019), we obtain all the triangulations using MATLAB.

**Figure 6:**
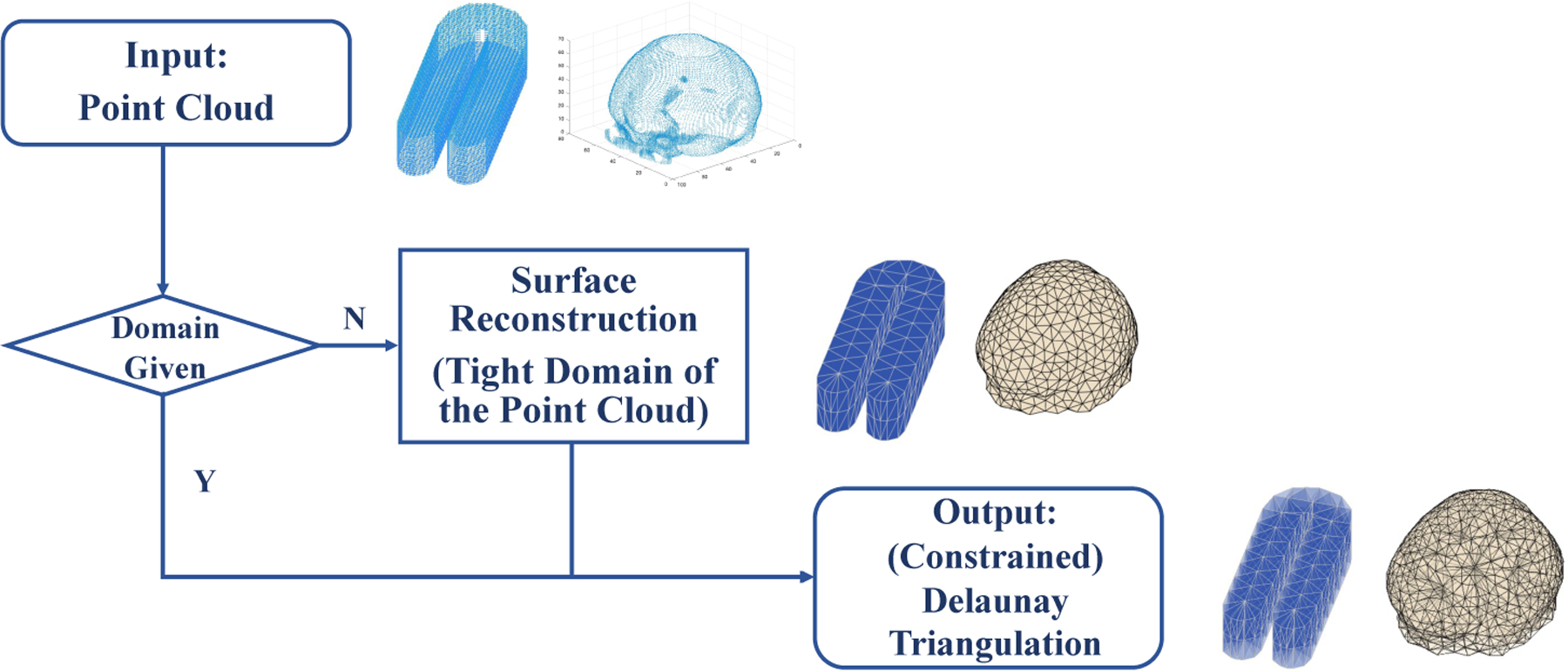
Flowchart demonstrating the process of constructing a triangulation from a 3D point cloud.

Figure 6 illustrates our flowchart for obtaining a triangulation. This generation procedure can be applied to many other 3D point clouds collected in various fields, as shown in the triangulation examples in Figure 3.

### 2.3 Trivariate Splines on a Triangulation

Suppose we have obtained a triangulation Δ for a tight domain Ω of a given point cloud. For any tetrahedron $T=\left\langle v_{1},v_{2},v_{3},v_{4}\right\rangle \in \Delta$, any point $p=(x,y,z)\in R^{3}$ has a unique representation in terms of $\left\langle v_{1},v_{2},v_{3},v_{4}\right\rangle$,

$$p=b_{1}v_{1}+b_{2}v_{2}+b_{3}v_{3}+b_{4}v_{4},\;\text{with }b_{1}+b_{2}+b_{3}+b_{4}=1,$$

where $\left(b_{1},b_{2},b_{3},b_{4}\right)$ are called the *barycentric coordinates* of p *relative to the tetrahedron* T, and they are nonnegative if p is inside or on the faces of T. Accordingly, for some nonnegative integers i,j,k,l with i+j+k+l=d, define *trivariate Bernstein basis polynomial of degree d relative to T* as

(2) $$B_{\text{ijkl}}^{d,T}(p)≔\frac{d!}{i!j!k!l!}b_{1}^{i}b_{2}^{j}b_{3}^{k}b_{4}^{l},\;\text{with }i+j+k+l=d\text{.}$$

For any positive integer d, let ${𝒫}_{d}$ be the space of all trivariate polynomials with total degrees less than or equal to d. Note that the dimension of ${𝒫}_{d}$ is $\left(\begin{array}{c} d+3\\ 3 \end{array}\right)$. According to Theorem 15.8 in Lai and Schumaker (2007) and Lemma A.6 in the Appendix A, the set of Bernstein basis polynomials in Equation 2 forms a basis for the space of polynomials ${𝒫}_{d}$. Thus, any polynomial $\phi (p)\in {𝒫}_{d}$ on with domain T can be written uniquely as the B-*form*,

(3) $$\phi (p)=\sum_{i+j+k+l=d}\;\gamma_{T;\text{ijkl}}B_{\text{ijkl}}^{d,T}(p)=B_{T}^{d}(p{)}^{⊤}\gamma_{T},$$

where the coefficients $\gamma_{T}={\left\{\gamma_{T;\text{ijkl}}\right\}}_{i+j+k+l=d}$ are called B-*coefficients* of ϕ(⋅).

For any nonnegative integer r, we use ${𝒞}^{r}(\Omega )$ to denote the collection of all r-th continuously differentiable functions over Ω. For triangulation $\Delta =\left\{T_{1},\ldots ,T_{N}\right\}$, let ${𝒮}_{d}^{r}(\Delta )=\left\{s\in {𝒞}^{r}(\Omega ):{\left.s\right|}_{T}\in {𝒫}_{d}(T),T\in \Delta \right\}$ be a spline space of degree d and smoothness r over Δ, where ${\left.s\right|}_{T}$ is the polynomial piece of spline s restricted on tetrahedron T. According to Equation 3, for any $s\in {𝒮}_{d}^{r}(\Delta )$, there exists a coefficient vector $\gamma ={\left(\gamma_{T_{1}}^{⊤},\ldots ,\gamma_{T_{N}}^{⊤}\right)}^{⊤}$ with $\gamma_{T_{J}}={\left\{\gamma_{T_{J};\text{ijkl}}\right\}}_{i+j+k+l=d}$ such that

(4) $${\left.s\right|}_{T_{J}}(p)=\sum_{i+j+k+l=d}\;\gamma_{T_{J};\text{ijkl}}B_{\text{ijkl}}^{d,T_{J}}(p)=B_{T_{J}}^{d}(p{)}^{⊤}\gamma_{T_{J}},J=1,\ldots ,N.$$

That is, $s(p)=B_{d}(p{)}^{⊤}\gamma$, where $B_{d}={\left\{{\left(B_{T_{1}}^{d}\right)}^{⊤},\ldots ,{\left(B_{T_{N}}^{d}\right)}^{⊤}\right\}}^{⊤}$.

For noise-free data, the approximation order of trivariate spline spaces was studied in Lai and Schumaker (2007) when d≥8r+1 and Lai (1989) when d≥6r+3. Particularly, Lai (1989) proved that when d≥6r+3, the space ${𝒮}_{d}^{r}(\Delta )$ can attain the optimal converge rate; see Lemma 1 below. For any index $\alpha =\left(\alpha_{1},\alpha_{2},\alpha_{3}\right)$ of order $|\alpha |=\alpha_{1}+\alpha_{2}+\alpha_{3}$, we denote the derivatives $D^{\alpha }s=\partial^{\left|\alpha \right|}s/\partial_{x}^{\alpha_{1}}\partial_{y}^{\alpha_{2}}\partial_{z}^{\alpha_{3}}$. For any function f over the closure of domain Ω, denote the $L^{q}(\Omega )$ norm (1≤q<∞) and supremum norm as $∥f{∥}_{L^{q}(\Omega )}={\left\{\int_{\Omega }\;|f(v){|}^{q}dv\right\}}^{1/q}$ and $∥f{∥}_{\infty ,\Omega }={\text{sup}}_{v\in \Omega }\;|f(v)|$, respectively. Next, for any k≥0, denote $|f{|}_{k,\infty ,\Omega }={\text{max}}_{|\alpha |=k}\;{∥D^{\alpha }f∥}_{\infty ,\Omega }$, and $|f{|}_{k,q,\Omega }=\sum_{|\alpha |\leq k}\;{∥D^{\alpha }f∥}_{L^{q}(\Omega )}$. Further, we define the Sobolev space ${𝒲}^{\ell ,q}(\Omega )=\left\{f:|f{|}_{k,q,\Omega }<\infty ,0\leq k\leq \ell \right\}$ for 1≤q≤∞,ℓ≥1.

**Lemma 1** *(Theorem 3.5.2 in* Lai, 1989*) For all* $f\in {𝒲}^{d+1,q}(\Omega )$ *with* 1≤q≤∞,r≥0, *and* d≥6r+3*, there exists a spline* $s_{f}\in {𝒮}_{d}^{r}(\Delta )$ *such that*

$${∥D^{\alpha }\left(f-s_{f}\right)∥}_{L^{q}(\Omega )}\leq K|\Delta {|}^{d+1-|\alpha |}|f{|}_{d+1,q,\Omega },$$

*for all* 0≤|α|≤m, where K>0 *is a constant independent of* f *and* |Δ| *but is dependent on the geometry of* Δ.

**Remark 2** *Both* d *and* r *are parameters for the spline space* ${𝒮}_{d}^{r}(\Delta )$*, which determines the smoothness of the TPST estimator and are usually predetermined by the user. Practically, as long as* d≥r, we can construct the spline bases. If d≥6r+3*, we can achieve the full approximation (approximation with optimal convergence rate) order, as shown in Lemma 1. A higher value of* d *indicates a higher degree polynomial, which can result in a higher computation burden; see our analysis in* Section 6*. In practice, the choice of* d *and* r *is closely tied to the intended interpretation of the estimated function. If the goal is to enhance the signal-to-noise ratio for visualization or to suggest a simple parametric model, then a slightly oversmoothed function with a subjectively chosen parameter may be appropriate. However, if the focus is on accurately estimating the regression function and preserving local structures, then a slightly undersmoothed function may be more suitable*.

## 3 Penalized Spline Estimators

With all the preparations in the previous introduction, we apply the trivariate spline over the triangulation to recognize the underlying signal from 3D point clouds in this section.

In the following, for any i=1,…,n, let point $p_{i}=\left(x_{i},y_{i},z_{i}\right)\in R^{3}$ be the location or design point of the ith observation in a point cloud of sample size n. Let $W_{i}$ be the response variable observed on the ith location $p_{i}$. Then, we regard any point cloud as a set of n observations ${\left\{\left(p_{i},W_{i}\right)\right\}}_{i=1}^{n}$ in general. To extract the underlying signal, we consider the following nonparametric regression model

(5) $$W_{i}=m\left(p_{i}\right)+\sigma \left(p_{i}\right)\varepsilon_{i},$$

where m(⋅) is some smooth but unknown 3D function, σ(⋅) is the unknown conditional standard deviation function, and $\varepsilon_{i}$ is the random error term with mean zero and variance one. Assume $\left(W_{i},\varepsilon_{i}\right)$ are general iid copies of (W,ε).

In nonparametric smoothing, the roughness penalty approach is widely used when smoothing noisy data (Green and Silverman, 1994; Wood, 2003; Lai and Wang, 2013). Including a roughness penalty and choosing a proper tuning parameter can avoid overfitting problems and balance the bias and variance of the estimator of the function. To estimate the underlying function m in Equation 5, we formulate the roughness penalty approach as the following penalized least squares problem:

(6) $$\underset{s\in {𝒮}_{d}^{r}(\Delta )}{\text{min}}\;\sum_{i=1}^{n}\;{\left\{W_{i}-s\left(p_{i}\right)\right\}}^{2}+\rho_{n}ℰ\left(s\right),$$

where the roughness penalty

(7) $$ℰ\left(s\right)=\sum_{\left|\alpha \right|=2}\;\left(\begin{array}{c} 2\\ \alpha_{1} \end{array}\right)\left(\begin{array}{c} 2-\alpha_{1}\\ \alpha_{2} \end{array}\right)\int_{\Omega }\;{\left\{D^{\alpha }s\left(p\right)\right\}}^{2}\;\text{d}p,$$

and $\rho_{n}$ is the roughness penalty parameter. We aim to find the minimizer of Equation 6 in ${𝒮}_{d}^{r}(\Delta )$, denoted as ${\hat{m}}_{\rho_{n}}$, which is the *Trivariate Penalized Spline over triangulation* (TPST) estimator of m. The tuning parameter $\rho_{n}$ controls the smoothness of the fitted spline function. A larger $\rho_{n}$ leads to a less fluctuating function. If $\rho_{n}$ goes to infinity, our estimator shrinks to linear functions where the roughness penalty ℰ(s)=0. On the other hand, when $\rho_{n}=0$, the estimator becomes the standard unpenalized least squares spline estimator. A proper penalty parameter $\rho_{n}$ balances the goodness of fit for the data and the volatility of estimated functions.

The penalty ℰ(s) in Equation 7 is a commonly used penalty; see Green and Silverman (1994) for the 2D case. For a spline function $s\in {𝒮}_{d}^{r}(\Delta )$, combining Equation 4, the roughness penalty in Equation 7 can be written as follows:

(8) $$ℰ(s)=\sum_{T\in \Delta }\;ℰ\left({\left.s\right|}_{T}\right)=\sum_{T\in \Delta }\;\sum_{|\alpha |=2}\;\left(\begin{array}{c} 2\\ \alpha_{1} \end{array}\right)\left(\begin{array}{c} 2-\alpha_{1}\\ \alpha_{2} \end{array}\right)\int_{T}\;{\left\{{\left.D^{\alpha }s\right|}_{T}(p)\right\}}^{2}\;\text{d}p=\sum_{T\in \Delta }\;\gamma_{T}^{⊤}P_{T}\gamma_{T},$$

where $P_{T}$ is the corresponding penalty matrix. It is easy to show that $ℰ\left(B_{d}^{⊤}\gamma \right)=\gamma^{⊤}P\gamma$, where P is the block diagonal penalty matrix. See Section A.6 for more details in derivations and calculations of penalty matrices.

Since $s\in {𝒞}^{r}(\Delta )$, the coefficients of s satisfy some smoothness conditions across each interior faces of Δ. One can obtain a smoothness constraint matrix H such that Hγ=0 by repeatedly applying Equation A.7 in the Appendix A over all shared triangular faces, and more details are available in Section A.2 in the supplementary material. A MATLAB implementation is discussed in Awanou et al. (2006). Thus, the objective function can be written as

(9) $$\underset{\gamma }{\text{min}}\;\sum_{i=1}^{n}\;{\left\{W_{i}-B_{d}{\left(p_{i}\right)}^{⊤}\gamma \right\}}^{2}+\rho_{n}\gamma^{⊤}P\gamma \text{, subject to }H\gamma =0,$$

where H is the matrix for all smoothness conditions across shared edges or faces of tetrahedra, which depends on the smoothness parameter r and the structure of the triangulation. One can use QR decomposition to get rid of the constraint in Equation 9. Specifically, $H^{⊤}=QR=\left(Q_{1}Q_{2}\right)\left(\begin{array}{l} R_{1}\\ R_{2} \end{array}\right)$, where Q is an orthogonal matrix, R is an upper triangular matrix, $R_{1}$ is a full rank matrix with the same rank as H, and $R_{2}$ is a matrix of zeros. Note that for any vector γ satisfying Hγ=0, there exists some θ such that $\gamma =Q_{2}\theta$. Also, for any $\theta ,H\left(Q_{2}\theta \right)=0$ holds. Thus, Equation 9 is equivalent to a penalized regression problem without constraint:

(10) $$\sum_{i=1}^{n}\;{\left\{W_{i}-B_{d}{\left(p_{i}\right)}^{⊤}Q_{2}\theta \right\}}^{2}+\rho_{n}\theta^{⊤}Q_{2}^{⊤}PQ_{2}\theta ,$$

which leads to a closed form of the solution. To be specific, let $W={\left(W_{1},\ldots ,W_{n}\right)}^{⊤}$, then the minimizer of Equation 10 is given by ${\hat{\theta }}_{\rho_{n}}={\left(Q_{2}^{⊤}B_{d}^{⊤}B_{d}Q_{2}+\rho_{n}Q_{2}^{⊤}PQ_{2}\right)}^{-1}Q_{2}^{⊤}B_{d}^{⊤}W$. Consequently, the spline coefficient in Equation 9 can be estimated by ${\hat{\gamma }}_{\rho_{n}}=Q_{2}{\hat{\theta }}_{\rho_{n}}$, which yields the TPST estimator ${\hat{m}}_{\rho_{n}}(p)=B_{d}(p{)}^{⊤}{\hat{\gamma }}_{\rho_{n}}$. Several methods can be used for choosing the penalty parameter $\rho_{n}$, such as the block cross-validation (block CV) and generalized cross-validation (GCV). Detailed discussion is given in Section 5.2.

## 4 Theoretical Results

In the previous section, we discuss how to construct TPST and capture the underlying signal using TPST from the point clouds. In the following, we investigate the theoretical support of TPST.

For random variables $X_{n},\;X_{n}=O_{P}\left(b_{n}\right)$ if ${\text{lim}}_{c\to \infty }\;{\text{lim}\;\text{sup}}_{n}\;P\left(\left|X_{n}\right|\geq cb_{n}\right)=0,n\geq 1$. Similarly, for any constant $c>0,\;X_{n}=o_{P}\left(b_{n}\right)$ if ${\text{lim}}_{n\to \infty }\;P\left(\left|X_{n}\right|\geq cb_{n}\right)=0$. And $a_{n}≍b_{n}$ if there exist two positive constants $c_{1},\;c_{2}$ such that $c_{1}\left|a_{n}\right|\leq \left|b_{n}\right|\leq c_{2}\left|a_{n}\right|$, for all n≥1.

### 4.1 Convergence Rate

Before we state our main results, we make the following assumptions, which are standard in nonparametric literature (Huang, 2003; Lai and Wang, 2013; Yu et al., 2020). Let $\epsilon_{i}=\sigma \left(p_{i}\right)\varepsilon_{i}$, and denote the generic variable of $\epsilon_{i}$ as ϵ.

(A1) The trivariate function $m\in {𝒲}^{\ell +1,\infty }(\Omega )$ for an integer ℓ≥1.
(A2) The noise ϵ satisfies that ${\text{lim}}_{\eta \to \infty }\;\text{E}\left[\epsilon^{2}I(|\epsilon |>\eta )\right]=0$ and $\text{E}\left|\epsilon^{2+\eta }\right|\leq v_{\eta }$ for some η>0. The standard deviation function σ(p) is continuous on Ω and $0<c_{\sigma }\leq {\text{inf}}_{p\in \Omega }\;\sigma (p)\leq {\text{sup}}_{p\in \Omega }\;\sigma (p)\leq C_{\sigma }<\infty$.
(A3) The density function of the observations is bounded below and above.
(A4) The number of the tetrahedra N and the sample size n satisfy that $N={\text{Cn}}^{\gamma }$ for some constant C>0 and γ<η/(2+η), where η is given in the Assumption (A2).

To obtain the asymptotic analysis of spline estimators, we account on an important property that the data-driven norm uniformly approach to its expectation uniformly over the entire spline space. To see this, for any function f over the closure of domain Ω, let ${\text{E}}_{n}(f)=n^{-1}\sum_{i=1}^{n}\;f\left(p_{i}\right)$ and E(f)=E[f(p)]. Define the empirical inner product and norm as ${\left\langle f_{1},f_{2}\right\rangle }_{n,\Omega }={\text{E}}_{n}\left(f_{1}f_{2}\right)$ and ${∥f_{1}∥}_{n,\Omega }^{2}={\left\langle f_{1},f_{1}\right\rangle }_{n,\Omega }$, respectively, for measurable functions $f_{1}$ and $f_{2}$ on Ω. The theoretical $L^{2}$ inner product and the induced norm are given by ${\left\langle f_{1},f_{2}\right\rangle }_{L^{2}(\Omega )}=\text{E}\left(f_{1}f_{2}\right)$ and ${∥f_{1}∥}_{L^{2}(\Omega )}^{2}={\left\langle f_{1},f_{1}\right\rangle }_{L^{2}(\Omega )}$. We illustrate the uniform convergence rate of empirical inner product to the theoretical one in the following Lemma 3.

**Lemma 3** *Denote the basis for* ${𝒮}_{d}^{r}(\Delta )$ *constructed in* Lai and Schumaker (2007) *by* ${\left\{B_{\xi }\right\}}_{\xi \in ℳ}$*, where* ℳ *stands for the index set of spline bases. Let $g_{1}=\sum_{\xi \in ℳ}\;c_{\xi }B_{\xi },g_{2}=\sum_{\zeta \in ℳ}\;{\tilde{c}}_{\zeta }B_{\zeta }$ be any spline functions in ${𝒮}_{d}^{r}(\Delta )$. Under Assumptions (A3) and (A4), we have*

$$R_{n}=\underset{g_{1},g_{2}\in {𝒮}_{d}^{r}(\Delta )}{\text{sup}}\;\left|\frac{{\left\langle g_{1},g_{2}\right\rangle }_{n,\Omega }-{\left\langle g_{1},g_{2}\right\rangle }_{\Omega }}{{∥g_{1}∥}_{\Omega }{∥g_{2}∥}_{\Omega }}\right|=O_{P}\left\{(N\;\text{log}\;n{)}^{1/2}n^{-1/2}\right\}.$$

For the purpose of illustrating theoretical development, we rewrite the penalty in Equation 7 in terms of linear operation. Let ℬ≔ℬ(Ω) be the space of all bounded real-valued functions over $\Omega =\cup_{T\in \Delta }T$ equipped with the inner product $n{\left\langle f_{1},f_{2}\right\rangle }_{n,\Omega }+\rho_{n}{\left\langle f_{1},f_{2}\right\rangle }_{ℰ}$, where

$${\left\langle f_{1},f_{2}\right\rangle }_{ℰ}=\sum_{|\alpha |=2}\;\left(\begin{array}{c} 2\\ \alpha_{1} \end{array}\right)\left(\begin{array}{c} 2-\alpha_{1}\\ \alpha_{2} \end{array}\right)\sum_{T\in \Delta }\;\int_{T}\;\left\{D^{\alpha }f_{1}(p)\right\}\left\{D^{\alpha }f_{2}(p)\right\}\text{d}p$$

for $f_{1},f_{2}\in ℬ$.

Next, we introduce a measure of the complexity of the spline space ${𝒮}_{d}^{r}(\Delta )$, and another measure which bounds the size of the derivatives:

(11) $$V_{n}=\underset{g\in {𝒮}_{d}^{r}(\Delta )}{\text{sup}}\;\left\{\frac{∥g{∥}_{\infty ,\Omega }}{∥g{∥}_{n,\Omega }},∥g{∥}_{n,\Omega }\neq 0\right\},\;{\overset{‾}{V}}_{n}=\underset{g\in {𝒮}_{d}^{r}(\Delta )}{\text{sup}}\;\left\{\frac{∥g{∥}_{ℰ}}{∥g{∥}_{n,\Omega }},∥g{∥}_{n,\Omega }\neq 0\right\}.$$

These two measures will play an important role in developing the asymptotic results. We use the following Lemma 4 to demonstrate the upper bounds of $V_{n}$ and ${\overset{‾}{V}}_{n}$.

**Lemma 4** *Under Assumptions (A3) and (A4), we have $V_{n}=O_{P}\left(|\Delta {|}^{-3/2}\right),{\overset{‾}{V}}_{n}=O_{P}\left(|\Delta {|}^{-2}\right)$*.

We then define a linear operator $P_{\rho_{n}}:ℬ\mapsto {𝒮}_{d}^{r}(\Delta )$ such that $P_{\rho_{n}}W={\hat{m}}_{\rho_{n}}$. Note that in general, $P_{\rho_{n}}$ is not a linear projection. Thus, we have $P_{\rho_{n}}W=P_{\rho_{n}}m+P_{\rho_{n}}\epsilon$, where $P_{\rho_{n}}m$ and $P_{\rho_{n}}\epsilon$ are the penalized spline estimators based on ${\left\{m\left(p_{i}\right)\right\}}_{i=1}^{n}$ and ${\left\{\epsilon_{i}\right\}}_{i=1}^{n}$, respectively. Under some conditions (Huang, 2003), $P_{0}$ is a bounded operator on ${𝒮}_{d}^{r}(\Delta )$ in the maximum norm. Denote $s_{\rho_{n},m}=P_{\rho_{n}}m$ and $s_{\rho_{n},\epsilon }=P_{\rho_{n}}\epsilon$. Consequently, for the penalized spline estimator ${\hat{m}}_{\rho_{n}}$ in Equation 6,

(12) $${\hat{m}}_{\rho_{n}}(p)-m(v)=\left\{s_{\rho_{n},m}(p)-m(p)\right\}+s_{\rho_{n},\epsilon }(p),$$

where $s_{\rho_{n},m}(p)-m(p)$ and $s_{\rho_{n},\epsilon }(p)$ are referred to as the bias and noise terms, respectively.

According to the error decomposition in Equation 12, to derive the convergence rate of ${\hat{m}}_{\rho_{n}}$ to m, it is sufficient to evaluate the size of the bias and noise terms. The following propositions provide the upper bound of the bias size and noise size.

**Proposition 5** *Under Assumptions (A1), (A3) and (A4), if* d≥6r+3 *and* Δ *is a* β*-quasi-uniform triangulation, then we have*

$${∥s_{\rho_{n},m}-m∥}_{\infty ,\Omega }=O_{P}\left\{\frac{\rho_{n}}{n|\Delta {|}^{7/2}}|m{|}_{2,\infty ,\Omega }+\left(1+\frac{\rho_{n}}{n|\Delta {|}^{11/2}}\right)|\Delta {|}^{\ell +1}|m{|}_{\ell +1,\infty ,\Omega }\right\}.$$

**Proposition 6** *Under Assumptions (A2) and (A4), ${∥s_{\rho_{n},\epsilon }∥}_{L^{2}(\Omega )}=O_{P}\left(\frac{1}{\sqrt{n}|\Delta {|}^{3/2}}\right)$*.

**Proposition 7** *Under Assumptions (A2) and (A4), ${∥s_{\rho_{n},\epsilon }∥}_{\infty ,\Omega }=O_{P}\left\{\frac{(\text{log}n{)}^{1/2}}{\sqrt{n}|\Delta {|}^{3/2}}+\frac{\rho_{n}}{n^{3/2}|\Delta {|}^{7}}\right\}$*.

Based on Propositions 5—7, we illustrate the convergence rates of the TPST estimator in the following Theorem 8, in terms of both the $L^{2}(\Omega )$ and supremum norms.

**Theorem 8** *Under Assumptions (A1)—(A4), if* d≥6r+3 *and* Δ *is a* β*-quasi-uniform triangulation, we have*

$${∥{\hat{m}}_{\rho_{n}}-m∥}_{L^{2}(\Omega )}=O_{P}\left\{\frac{\rho_{n}}{n|\Delta {|}^{7/2}}|m{|}_{2,\infty ,\Omega }+\left(1+\frac{\rho_{n}}{n|\Delta {|}^{11/2}}\right)|\Delta {|}^{\ell +1}|m{|}_{\ell +1,\infty ,\Omega }+\frac{1}{\sqrt{n}|\Delta {|}^{3/2}}\right\},$$

$${∥{\hat{m}}_{\rho_{n}}-m∥}_{\infty ,\Omega }=O_{P}\left\{\frac{\rho_{n}}{n|\Delta {|}^{7/2}}|m{|}_{2,\infty ,\Omega }+\left(1+\frac{\rho_{n}}{n|\Delta {|}^{11/2}}\right)|\Delta {|}^{\ell +1}|m{|}_{\ell +1,\infty ,\Omega }\right.+\left.\frac{(\text{log}\;n{)}^{1/2}}{\sqrt{n}|\Delta {|}^{3/2}}+\frac{\rho_{n}}{n^{3/2}|\Delta {|}^{7}}\right\}.$$

**Remark 9** *For the unpenalized spline estimator, that is,* $\rho_{n}=0$*, if one takes* $N≍n^{3/(2\ell +5)}$*, then* ${∥{\hat{m}}_{0}-m∥}_{L^{2}(\Omega )}=O_{P}\left(n^{-(\ell +1)/(2\ell +5)}\right)$, *which achieves the optimal convergence rate as shown in* Stone (1982)*. Similarly, for the supremum norm, one can obtain that* $∥{\hat{m}}_{0}-m{∥}_{\infty ,\Omega }=O_{P}\left\{{\left(n^{-1}\text{log}n\right)}^{\left(\ell +1)/(2\ell +5\right)}\right\}$ *when* $N≍(n/\text{log}n{)}^{3/(2\ell +5)},$ *which is also the optimal rate of convergence. When* $\rho_{n}>0$, *one can obtain optimal convergence rates for both* $L^{2}$ *and supremum norms with $\rho_{n}=o\left(n^{\left(\ell +1/2)/(2\ell +5\right)}\right)$ and same orders of* N.

**Remark 10** *Assumption (A3) is standard in nonparametric literature. Even though this assumption may not be satisfied with missing data, we usually can find a proper choice of triangulation by adjusting the size of some of the tetrahedra to obtain a decent penalized spline fitting*.

### 4.2 Asymptotic Normality

To derive the asymptotic normality of the TPST estimator, we further assume the following conditions.

(A4’) The number of the tetrahedra N and the sample size n satisfy that $N=Cn^{\gamma }$ for some constant C>0 and 1/(ℓ+2)<γ<η/(2+η), where ℓ and η are given in the Assumptions (A1) and (A2), respectively.
(A5) The penalized parameter $\rho_{n}$ satisfies $\rho_{n}=o\left(n^{1/2}N^{-2/3}\wedge nN^{-4/3}\right)$.

**Remark 11** *A sufficient condition for a negligible bias term is provided by Assumptions (A4’) and (A5). Compared with Assumption (A4) in* Section 4.1*, (A4’) further assumes that the number of tetrahedra needs to be greater than a lower bound which depends on the degree of the function. A similar assumption for the univariate case has been discussed in* Li and Ruppert (2008)*. Meanwhile, assumption (A5) requires smaller $\rho_{n}$, which reduces the bias through under smoothing*.

**Theorem 12** *Under Assumptions (A1)—(A3), (A4’) and (A5), as* n→∞, *for each* p∈Ω,

$$\frac{{\hat{m}}_{\rho_{n}}\left(p\right)-m\left(p\right)}{\sqrt{\text{Var}\left\{{\hat{m}}_{\rho_{n}}\left(p\right)|P\right\}}}\overset{D}{\to }N(0,\;1),$$

*where* P *is the collection of all $p_{i},\;i=1,\ldots ,n$*.

**Remark 13** The above asymptotic distribution result can be used to construct asymptotic confidence intervals in theory. For example, if we estimate m(p) *using piecewise constant splines, Lemma* B.6 *in the* Appendix B *gives the size of the pointwise variance $\text{Var}\left\{{\hat{m}}_{\rho_{n}}(p)\right\}=\sigma^{2}(p){\left\{nf(p)V_{T}\right\}}^{-1}\{1+o(1)\},p\in \Omega$, where* $V_{T}$ is the volume of tetrahedron T. Therefore, an asymptotic 100(1-α)% pointwise confidence envelop for m(p) *over* Ω *is* ${\hat{m}}_{\rho_{n}}(p)\pm z_{\alpha /2}\sigma (p){\left\{nf(p)V_{T}\right\}}^{-1/2}$*, where* f(⋅) *stands for the density function of $p_{i}$. However, it is very difficult to obtain the exact form of the standard error for general TPST estimators due to the characteristic of the trivariate spline basis functions. To overcome this,* Section 5.4 *proposes using a wild bootstrap method to estimate the standard errors and quantify the uncertainty of the estimators*.

## 5 Implementation Details

This section provides some implementation details on how to construct the penalty matrix P and select the penalty parameter $\rho_{n}$ in Equation 9. To facilitate discussion, we first introduce the directional derivatives for basis functions, followed by the construction details of the penalty matrix, the selection criteria of the penalty parameter, and triangulation selection.

### 5.1 Construction of Penalty Matrix

We list here the key steps in the construction of the penalty matrix P in Equation 8. One can refer to Section A.6 in Appendix A for more details.

For a general multivariate smooth function ϕ, the directional derivative at point p with respect to direction u is defined as

$$D_{u}\phi (p)≔{\left.\frac{\partial }{\partial t}\phi (p+tu)\right|}_{t=0}=\underset{t\to 0}{\text{lim}}\;\frac{\phi (p+tu)-\phi (p)}{t}.$$

Accordingly, for vector $u≔\left(u_{x},u_{y},u_{z}\right)\in R^{3}$ and trivariate function ϕ, the directional derivative at p=(x,y,z) is

$$D_{u}\phi (x,y,z)≔{\left.\frac{\partial }{\partial t}\phi \left(x+tu_{x},y+tu_{y},z+tu_{z}\right)\right|}_{t=0}.$$

Then for the introduced Bernstein basis function with degree d, based on Lemma A.3 in Appendix A, we have

$$D_{u}B_{\text{ijkl}}^{d}(p)=d\left\{a_{1}B_{i-1,j,k,l}^{d-1}(p)+a_{2}B_{i,j-1,k,l}^{d-1}(p)+a_{3}B_{i,j,k-1,l}^{d-1}(p)+a_{4}B_{i,j,k,l-1}^{d-1}(p)\right\},$$

where $\left(a_{1},a_{2},a_{3},a_{4}\right)$ is the barycentric coordinate of direction u. Based on this conclusion, for a tetrahedron $s_{T}$, the corresponding penalty term can be written as

$$ℰ\left(s_{T}\right)\;=\sum_{|\alpha |=2}\;\;\left(\begin{array}{c} 2\\ \alpha_{1} \end{array}\right)\left(\begin{array}{c} 2-\alpha_{1}\\ \alpha_{2} \end{array}\right)\int_{T}\;\;{\left\{\sum_{i+j+k+l=d}\;\;\gamma_{T;\text{ijkl}}D^{\alpha }B_{\text{ijkl}}^{d,T}(p)\right\}}^{2}\;\text{d}p\;=\sum_{|\alpha |=2}\;\;\gamma_{T}^{⊤}P_{T}^{\alpha }\gamma_{T}=\gamma_{T}^{⊤}P_{T}\gamma_{T},$$

where each $P_{T}^{\alpha }$ is a $\left(\begin{array}{c} d+3\\ 3 \end{array}\right)\times \left(\begin{array}{c} d+3\\ 3 \end{array}\right)$ matrix with entries $\int_{T}\;\left\{D^{\alpha }B_{\text{ijkl}}^{d,T}(p)\right\}\left\{D^{\alpha }B_{i^{\prime }j^{\prime }k^{\prime }l^{\prime }}^{d,T}(p)\right\}\text{d}p$ for α satisfying |α|=2. As for the penalty term defined in the whole domain, recall that $ℰ(s)=\sum_{T\in \Delta }\;ℰ\left(s_{T}\right)$ and $ℰ\left(s_{T}\right)=\gamma_{T}^{⊤}P_{T}\gamma_{T}$. Therefore, $ℰ(s)=\gamma^{⊤}P\gamma$, where $\gamma ={\left(\gamma_{1}^{⊤},\ldots ,\gamma_{N}^{⊤}\right)}^{⊤}$, and $P=\text{diag}\left(P_{T},T\in \Delta \right)$ is a block diagonal matrix.

### 5.2 Penalty Parameter Selection

To balance the bias and variance of the proposed estimator and achieve a good estimation and prediction performance, it is crucial to choose a suitable value of the penalty parameter $\rho_{n}$. Since the in-sample fitting errors can not gauge the prediction accuracy of the fitted function, we select a criterion function that attempts to measure the out-of-sample performance of the fitted model. Minimizing the generalized cross-validation (GCV) criterion is one computationally efficient approach to selecting smoothing parameters that also has good theoretical properties.

**Figure 7:**
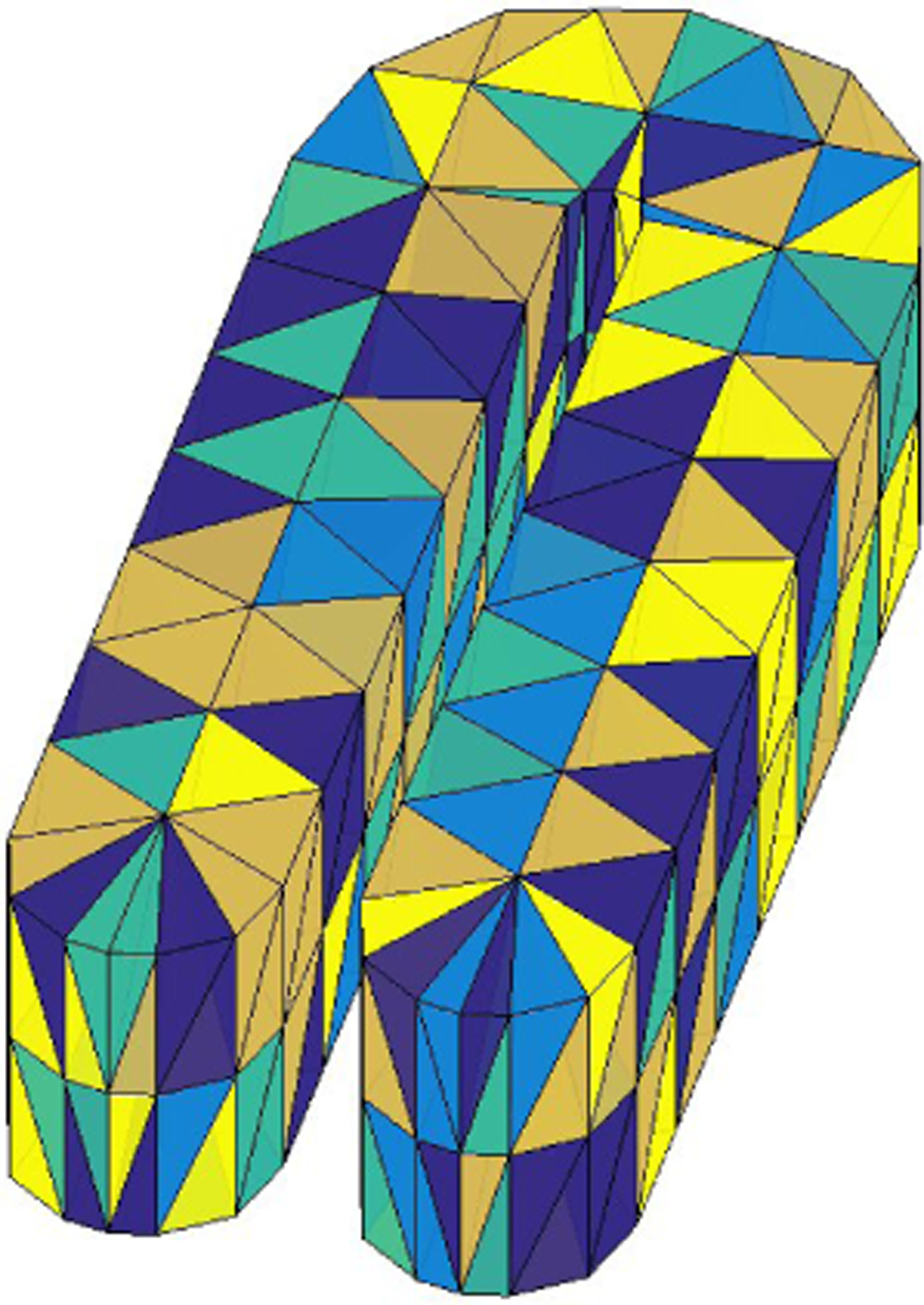
An illustration of block CV using a triangulation. Tetrahedra with the same color belong to the same fold.

Note that 3D object data are often generated with spatial dependence. When performing cross-validation (CV), these dependence structures are usually ignored, leading to underestimating the predictive error (Roberts et al., 2017). To tackle this problem, one can adopt the block CV strategy (Roberts et al., 2017; Valavi et al., 2019). To be specific, all the sample points are first divided into 3D blocks with similar volumes. Then, these blocks are randomly allocated to the CV folds. In this paper, we adopt the triangulation to divide the 3D domain into small 3D blocks. Each tetrahedron is considered as one single 3D block. Figure 7 shows an example of block CV using a triangulation. In Figure 7, we divide the domain into 504 tetrahedra and randomly assign these tetrahedra into five folds with colors indicating different folds.

### 5.3 Triangulation Selection

As illustrated in Figure 6, to form a proper triangulation, we start with surface reconstruction. A wide range of techniques has been developed to reconstruct the surface from point clouds. Our paper focuses on the methods that generalize a well-sampled point cloud to arbitrary shapes and produce a watertight surface mesh, such as a triangular mesh. Since we are interested in recovering the actual signal within the 3D point clouds, we skip the details of the surface reconstruction here. The triangulation surface representations can be coarse or fine and affect the construction of the triangulation. A very coarse triangulation could give a poor approximation to the object, while a very fine triangulation could introduce a more serious computation burden. Note that Assumption (A4’) requires that the number of the tetrahedra, N, is larger than some minimum depending upon the degree of the spline, so theoretically, we can determine the fineness of a triangulation by setting $N\approx \left\lfloor c_{1}n^{\gamma }\text{log}(n)\right\rfloor +c_{2}$, in which ⌊·⌋ denotes the integer part, $c_{1},\;c_{2}$ are tuning constants, and 1/(ℓ+2)<γ<1. In practice, we can implement the proposed method in a coarse-tofine resolution manner with similar criteria discussed in Section 5.2. Our extensive Monte Carlo simulation studies suggest that once a triangulation is fine enough, further refinement usually has little effect on the fitting accuracy. To avoid model over-fitting, we can stop the partition refinement when the model performance on the test set sits flat or even worsens.

### 5.4 Uncertainty Studies

In this section, we adopt a bootstrap method to quantitatively estimate the uncertainty of the TPST estimator. A great advantage of the bootstrap method is its simplicity, which can be a straightforward way to derive estimates of standard deviations and confidence intervals for estimators of functions over complex domains. As shown in the model in Equation 5, the assumption of homoscedasticity is often invalid when a regression model is used to estimate the point clouds. The wild bootstrap (Mammen, 1993; Hall and Horowitz, 2013) is specifically designed to work when the model is heteroscedastic. We conduct the following wild bootstrap procedure for estimating the standard errors.

Step 1. Based on the data ${\left\{\left(p_{i},W_{i}\right)\right\}}_{i=1}^{n}$, obtain the the TPST estimator $\hat{m}\left(p_{i}\right)$ described in Section 3 and the following residuals ${\hat{\epsilon }}_{i}=W_{i}-\hat{m}\left(p_{i}\right),i=1,\ldots ,n$.
Step 2. Generate the bootstrap residuals ${\left\{\epsilon_{i}^{*}\right\}}_{i=1}^{n}$ by $\epsilon_{i}^{*}=\delta_{i}{\hat{\epsilon }}_{i}$, where $\delta_{i}=\frac{1\pm \sqrt{5}}{2}$ with probability $\frac{5\pm \sqrt{5}}{10}$, respectively. Define $W_{i}^{*}={\hat{W}}_{i}+\epsilon_{i}^{*}$.
Step 3. Apply the TPST estimator to the sample ${\left\{\left(p_{i},W_{i}^{*}\right)\right\}}_{i=1}^{n}$, and obtain the estimated function ${\hat{m}}^{*}(\cdot )$ over the entire domain.
Step 4. Repeat Steps 2 and 3 B times and obtain a bootstrap sample of the TPST estimator as ${\left\{{\hat{m}}_{b}^{*}(\cdot )\right\}}_{b=1}^{B}$. Then the standard deviation of $\hat{m}(p),p\in \Omega$, is estimated by
$${\left[\frac{1}{B}\sum_{b=1}^{B}\;\;{\left\{{\hat{m}}_{b}^{*}(p)-{\overset{-}{\hat{m}}}^{*}(p)\right\}}^{2}\right]}^{1/2},$$
  where ${\bar{\hat{m}}}^{*}\left(\cdot \right)=B^{-1}{\sum \text{​}}_{b=1}^{B}{\hat{m}}_{b}^{*}\left(\cdot \right)$.

## 6 Numerical Studies

In this section, we conduct various simulation studies to assess the performance of the proposed TPST method. We use studies in Sections 6.1 and 6.2 to illustrate the capabilities of TPST in handling a variety of complex data structures, Section 6.3 to evaluate the uncertainties of the TPST estimator, and Section 6.4 to show the superiority of TPST in data reduction as well as signal enhancement, compared to other smoothing methods. More specifically, depending on the nature of the point clouds, we conduct experiments with unstructured (random design) and structured (fixed design) point clouds. The case of random design in Section 6.1 mimics the scenario that the point clouds are collections of 3D points distributed randomly in space. In contrast, the case of structured design is studied in Section 6.2. In this structured design setting, the point cloud is reconstructed from grid data; in other words, the locations of the points are deterministic. The evaluation of standard error for the proposed TPST estimator is illustrated in Section 6.3. In Section 6.4, we explore the practical performance of TPST on a structured point cloud by using PET scan data as a reference.

### 6.1 Unstructured Complete Point Clouds

**Figure 8:**
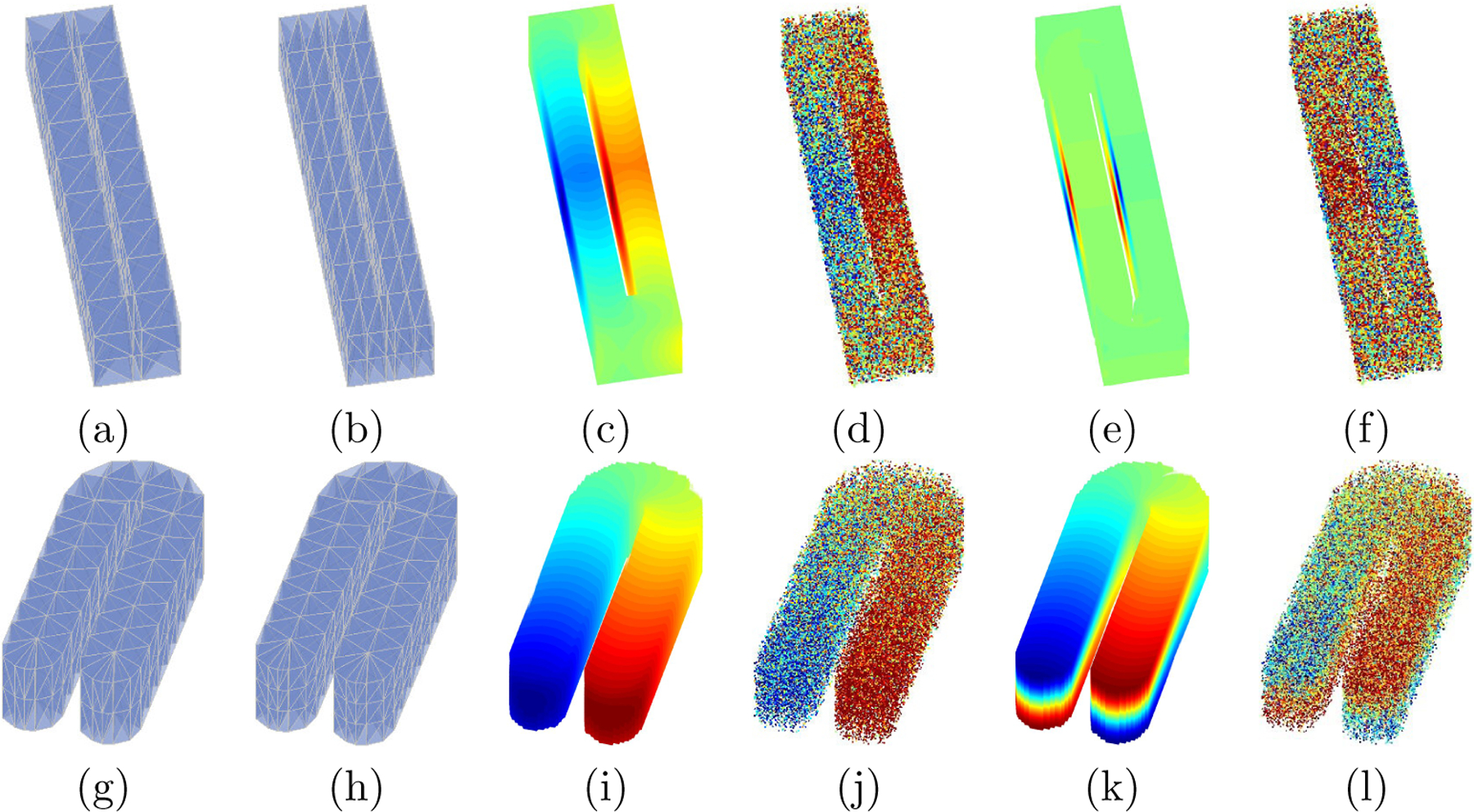
Triangulations, true functions and observed point clouds. Plots (a)—(f), are based on domain $\Omega_{1}$; Plots (g)—(l), are based on domain $\Omega_{2}$: (a) $\Delta_{11}$ for $\Omega_{1}$; (b) $\Delta_{12}$ for $\Omega_{1}$; (c) true function $m_{1}$; (d) observed point clouds with underlying function $m_{1}$; (e) true function $m_{2}$; (f) observed point clouds with underlying function $m_{2}$; (g) $\Delta_{21}$ for $\Omega_{2}$; (h) $\Delta_{22}$ for $\Omega_{2}$; (i) true function $m_{3}$; (j) observed point clouds with underlying function $m_{3};(\text{k})$ true function $m_{4}$; (l) observed point clouds with underlying function $m_{4}$.

In this example, we consider an unstructured (random design) point cloud in which the observations are randomly generated over the entire domain. We set the number of observations in each point cloud as n=20,000 and 50, 000. To mimic some complicated scenarios in practice, we generate the point clouds from the following two domains: (i) a cuboid with a hole inside $\left(\Omega_{1}\right)$; (ii) a 3D horseshoe $\left(\Omega_{2}\right)$. These domains are illustrated in Figure 8. To extract the underlying signal from point clouds, we consider the model in Equation 5, where the random noises, $\epsilon_{i}$’s, are assumed to be independent and identically distributed and follow a normal distribution $N\left(0,\sigma^{2}\right)$. For each domain, we consider two types of underlying functions with different degrees of variation: $m_{1}$ and $m_{2}$ for $\Omega_{1}$ (Figure 8 (c) and (e)), and $m_{3}$ and $m_{4}$ for $\Omega_{2}$ (Figure 8 (i) and (k)). The noises level, σ, is chosen according to the peak signal-to-noise ratios (PSNR) defined as

$$\text{PSNR}=20{\;\text{log}}_{10}\left\{\underset{i}{\text{max}}\;m\left(p_{i}\right)/\sigma \right\}.$$

In this study, we set PSNR = 5 and 10, representing scenarios of high and moderate noise levels, respectively. We use this study to investigate the effect of the size of the point cloud, degree of spline polynomial, and triangulation. We also compare the proposed method with the traditional tensor product spline method (Stone, 1994) and thin plate spline smoothing (Wood, 2003). We implement the tensor product spline and the thin plate spline smoothing using the function “gam” in R package “mgcv”. For a fair comparison, we set the dimension of the basis used to represent the underlying function for the thin plate spline and tensor product spline to be very similar to the dimension we used for the TPST method. As a result, the dimension after the numerosity reduction is comparable among different methods.

**Figure 9:**
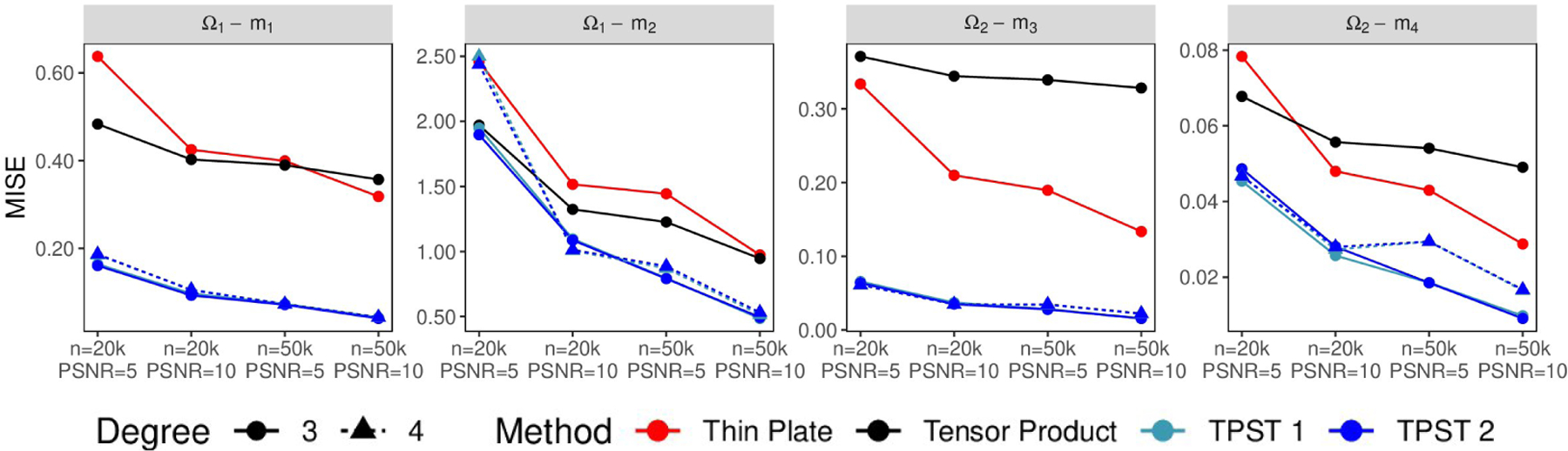
Average MISE plots for different methods on unstructured point clouds.

To evaluate the estimation and prediction performance of each method, we calculate the out-of-sample mean integrated squared error (MISE). Figure 9 presents the average of the MISEs over 200 replications for all different scenarios. Based on Figure 9, one can observe that as the size of the point cloud or the PSNR increases, the estimation and prediction accuracy improves for all the methods. Regardless of the scenarios, the proposed TPST method outperforms the other two traditional techniques with a similar dimension of numerosity reduction. These results indicate that the proposed TPST can better handle the “leakage” problem over the irregular domain than conventional smoothing methods.

To evaluate the effect of triangulations on the TPST, we consider a relatively coarse mesh $\Delta_{11}$ for $\Omega_{1}$ and $\Delta_{21}$ for $\Omega_{2}$, and a fine mesh $\Delta_{12}$ for $\Omega_{1}$ and $\Delta_{22}$ for $\Omega_{2}$. An illustration of these partitions is given in the Figure 8 (a), (b), (g), and (h), and the number of vertices and the number of tetrahedra are summarized in Table 1. From Figure 9, we can see that for each domain, the TPST estimator has a similar performance based on the two partitions. As discussed in Section 5.3, for TPST, when the number of the tetrahedra is sufficiently large to capture the pattern and features of the underlying function, more delicate triangulations will not benefit the estimation or the data reduction. For example, for domain $\Omega_{2}$, when we keep d=3, the remaining dimensions after the numerosity reduction are 323 and 340 for the two different triangulations, respectively.

Furthermore, based on Figure 9, one can see that the difference between estimates with d=3 and d=4 are relatively small. When we use a larger d, the estimators are usually less biased but with larger variance and more computationally intensive. In this example, d=3 is preferred as they are more efficient in data reduction. In general, the choice of d depends on the smoothness of the underlying function, the strength of signals, and computing resources.

As discussed previously, the spline methods are computationally efficient since they provide a global estimator. In this simulation example, the TPST method takes less than twenty seconds to fit the model for most of the simulation samples on a single Intel E5–2640 v3 core. The speed is comparable to tensor product and thin plate spline estimators.

### 6.2 Structured Point Clouds with Missing Data

**Figure 10:**
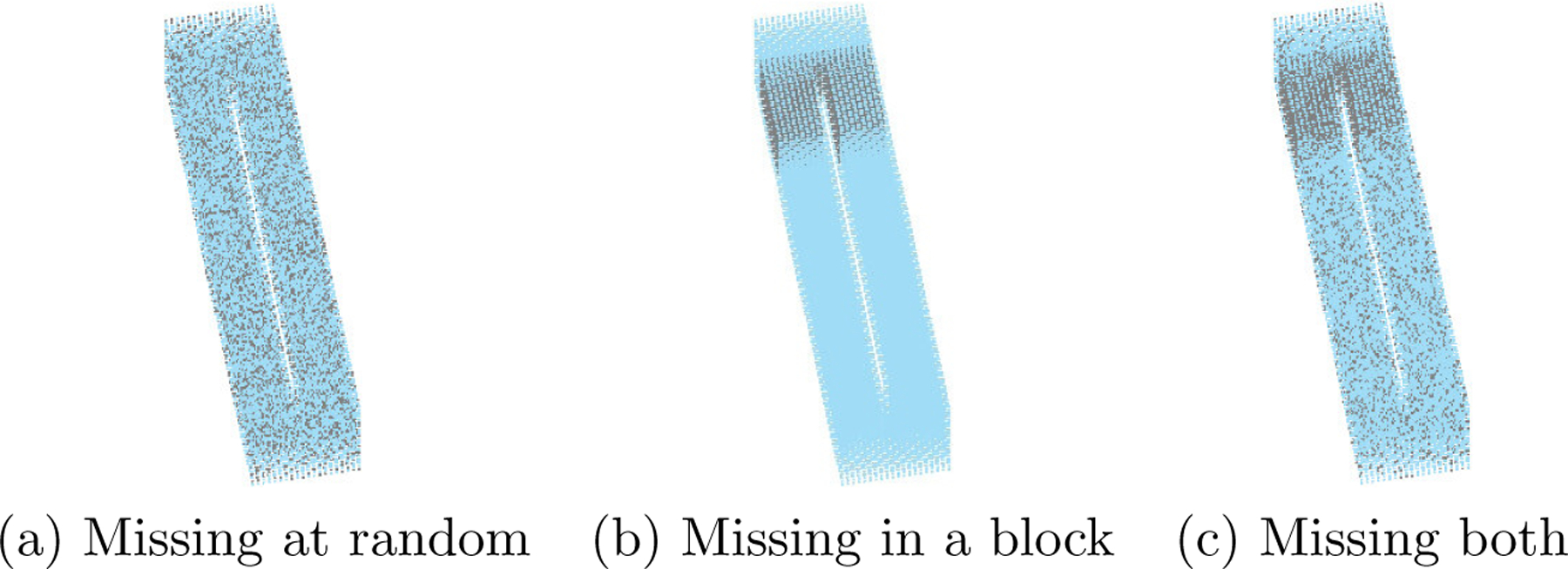
An illustration of three different missing patterns in point clouds. In (a) and (c), missing rate = 30% and in (b), missing rate = 12%.

One of the critical aspects of analyzing point cloud data is handling the unevenly distributed point clouds and/or missing values. We use this example to investigate further the proposed method on a structured point cloud at different resolutions with different missing schemes. To be more specific, we mimic three types of missing schemes, including (i) complete data with no missing, (ii) missing at random, and (iii) missing in a contiguous block as well as at random. Figures in Figure 10 illustrates different types of missing data we deal with in this example. Furthermore, we explore various missing rates under different missing mechanisms. For missing at random, we consider the missing rates ranging uniformly from 0 to 0.5, where 0 represents no missing voxels, and 0.5 means half of the voxels are missing. The contiguous block shown in Figure 10 contains 12% of the data.

Based on the first simulation example in Section 6.1, we can see that the performance for various methods is relatively consistent. Thus, we only consider the first domain, $\Omega_{1}$, and try the same triangulations in this example. We consider two fixed resolutions here: the lower resolution/scale is 60 × 20 × 20 with 22,160 voxels falling within the domain, and the higher resolution/scale is 75 × 25 × 25 with 42,600 voxels inside.

**Figure 11:**
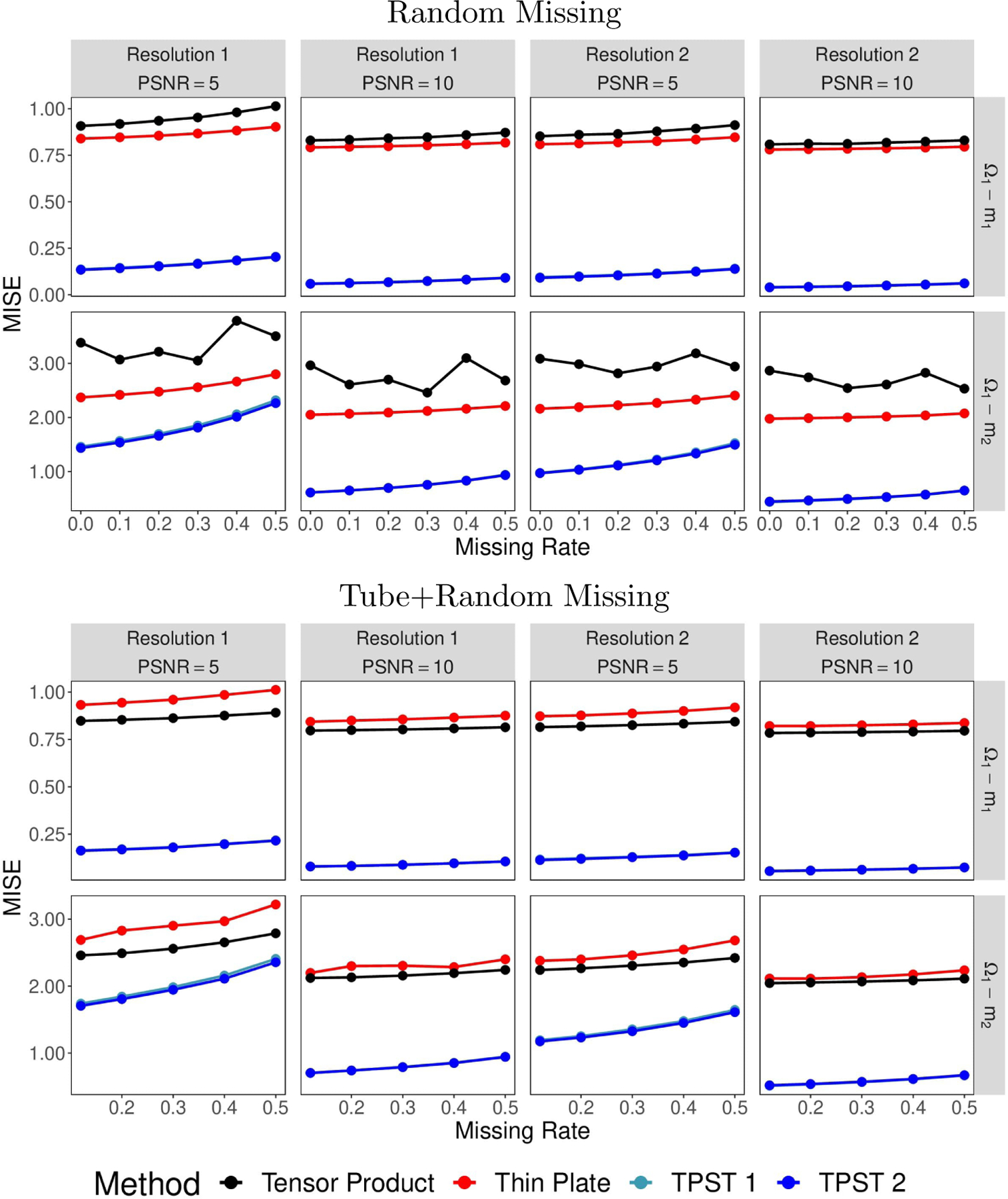
Average MISEs plots for different methods on structured point clouds with/without missing.

Similar to Section 6.1, we calculate the average MISEs over 200 replications with different missing types and missing rates and illustrated in Figure 11. Based on this Figure, one can see that the prediction accuracy improves for all of the methods as the missing rate decreases. The proposed methods outperform the two traditional methods regardless of the type of missing scheme and the missing rate. Furthermore, the missing type does not affect the proposed method very much. In contrast, the thin plate spline smoothing usually performs better for missing at random, while the tensor product spline is better when missing a contiguous block and at random.

### 6.3 Standard Error Evaluation

To evaluate the standard error (SE) for the proposed TPST estimator, we conduct another computational experiment using structured points clouds generated from the 3D horseshoe domain $\left(\Omega_{2}\right)$. We consider the model in Equation 5, where the underlying signal is illustrated in Figure 8 (i), and the random noises, $\epsilon_{i}=\sigma \left(p_{i}\right)\varepsilon_{i}$’s, are generated with $\sigma \left(p_{i}\right)=6-\left\{\left(x_{i}-\right.\right.\left.1.25{)}^{2}+y_{i}^{2}+z_{i}^{2}\right\}$ and $\varepsilon_{i}\sim N(0,\;1)$ iid. We consider a fixed solution/scale of 101 × 65 × 17 with 93, 449 voxels falling within the domain.

**Figure 12:**
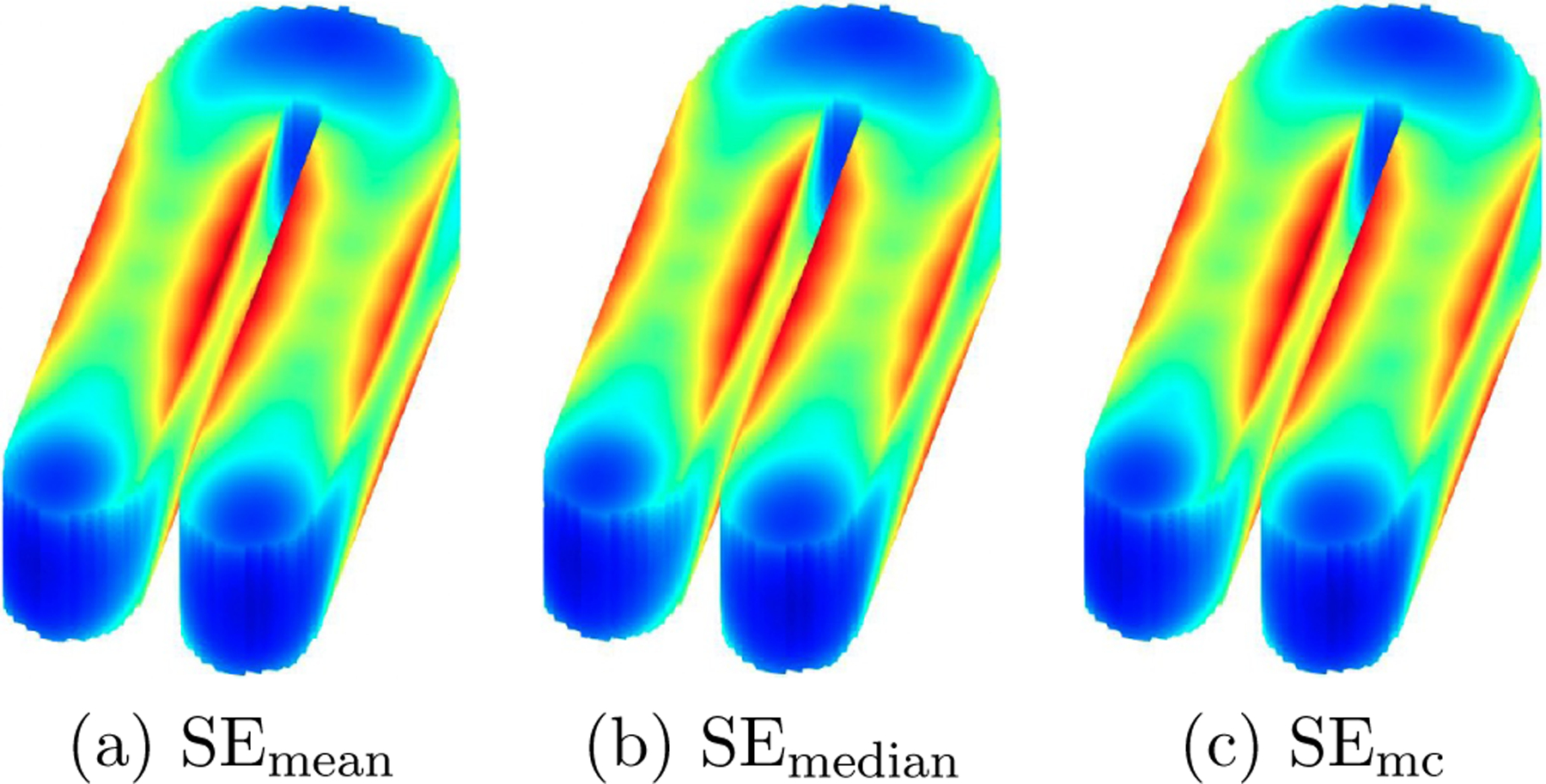
Standard error plot of the proposed TPST estimators: (a) and (b) are the mean $\left({\text{SE}}_{\text{mean}}\right)$ and median (${\text{SE}}_{\text{median}}$) of these bootstrap SEs across 200 replications, respectively; and (c) shows the standard deviation of the TPST estimator based on the 200 Monte Carlo samples (${\text{SE}}_{\text{mc}}$).

To quantify the uncertainty of the estimator, we generate 200 replications as in Sections 6.1 and 6.2. For each replication, we calculate the bootstrap SEs over 100 bootstrap samples using the wide bootstrap method introduced in Section 5.4. We then compute the the mean $\left({\text{SE}}_{\text{mean}}\right)$ and median (${\text{SE}}_{\text{median}}$) of these bootstrap SEs across 200 replications. Additionally, we calculate the standard deviation of the TPST estimator based on the Monte Carlo samples $\left({\text{SE}}_{\text{mc}}\right)$, which is served as the true value for SE. The results are displayed in Figure 12 (a) and (b) for ${\text{SE}}_{\text{mean}}$ and ${\text{SE}}_{\text{median}}$, respectively, and in Figure 12 (c) for ${\text{SE}}_{\text{mc}}$. From these plots, one sees that the ${\text{SE}}_{\text{mean}}$ and ${\text{SE}}_{\text{median}}$ is very close to ${\text{SE}}_{\text{mc}}$, which verifies the accuracy of the proposed bootstrap SE estimation method.

### 6.4 Biomedical Imaging Analysis

**Figure 13:**
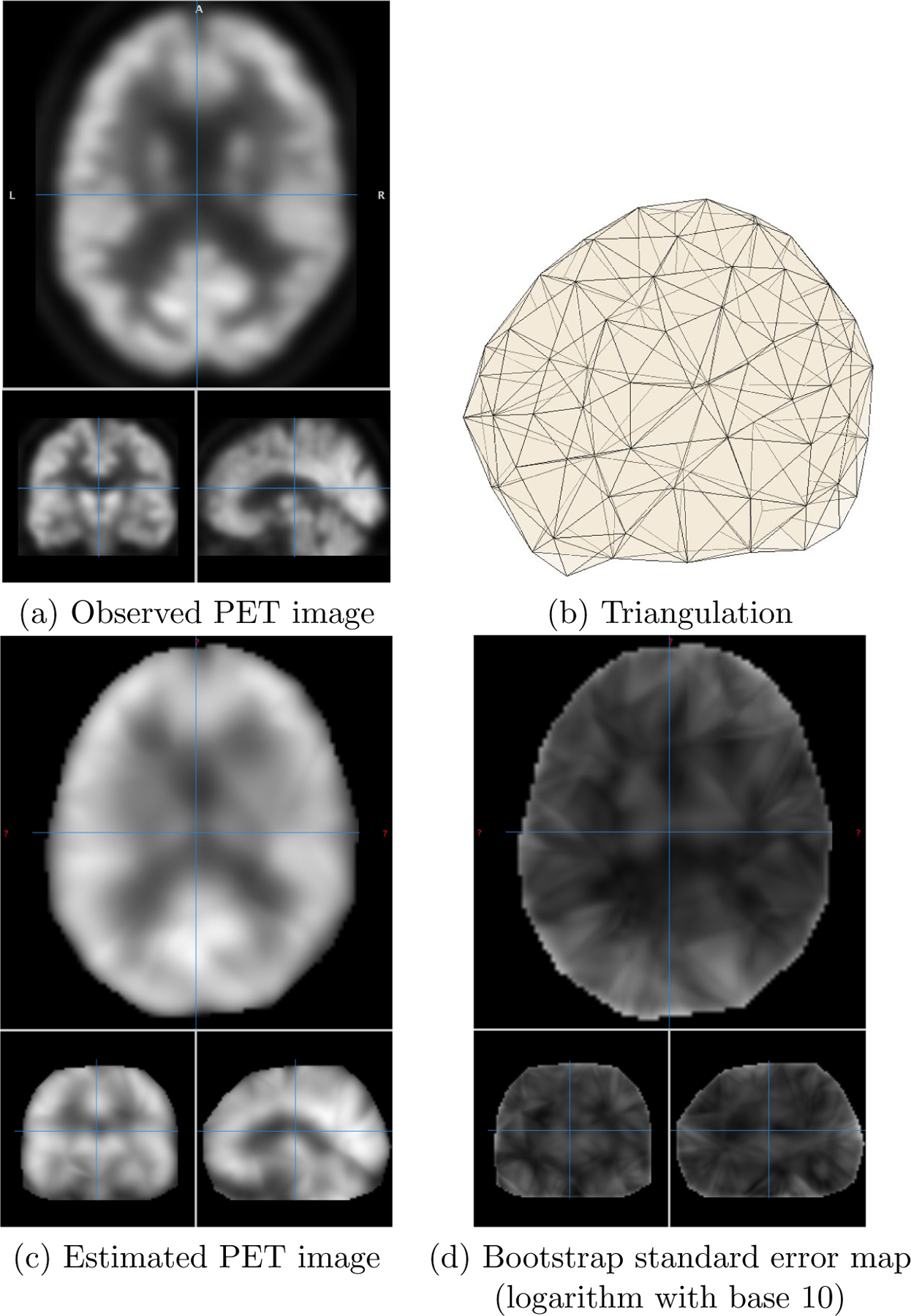
An illustration of an observed PET image, triangulations of the brain domain, estimated PET image, and corresponding bootstrap standard error map.

In our increasingly aging societies, Alzheimer’s disease (AD) has become the most frequent cause of dementia. Much progress has been made in assisting the early diagnosis of AD with neuroimaging techniques. One widely used neuroimaging technique is PET imaging, which can also be considered as an example of structured point clouds. However, the traditional PET scanning technique limits the overall resolution of the brain image, and there is a lack of effective and efficient image reconstruction methods. In this example, we apply the proposed TPST method to denoise and enhance the resolution of an actual PET image while improving the storage efficiency. A visual representation of transverse, coronal, and sagittal planes is shown in Figure 13 (a). Based on this figure, we can observe that brain images are collected on a rectangular parallelepiped grid with a dimension of 79 × 95 × 66. However, accurate signals are only present within the voxels of the human brain. As traditional methods, such as tensor product spline and thin plate spline, cannot handle complex domains, the model is trained using all the voxels within the entire image. In contrast, our proposed TPST method can manage complex domains efficiently. Therefore, we use a brain mask to determine the domain of the human brain, resulting in 280,000 voxels within the human brain domain. These masks can be obtained by many methods, such as building a PET template by averaging normalized PET images or testing the quantile value of each voxel to exceed a given threshold.

The results from Section 6.1 suggest that the choice of triangulation has a minimal effect on the performance of the TPST estimator as long as the number of tetrahedra is sufficient to capture the underlying pattern. In our implementation of the TPST method for brain imaging analysis, we use the triangulation depicted in Figure 13 (b) that consists of 317 tetrahedra and 117 vertices. To effectively capture high-frequency oscillations present in human brain scans, we set the polynomial degree d to at least 4. Additionally, we investigate the impact of different levels of global smoothness by considering r=0 and r=1. Figure 13 (c) shows an estimated PET image based on the settings d=5 and r=1, demonstrating the ability of the proposed TPST estimator to denoise the image while preserving the overall brain structure. To quantify the uncertainty of our estimations, we compute the bootstrap standard error as detailed in Section 5.4, and the logarithmic (base 10) transformation of the standard error map is presented in Figure 13 (d).

To compare the estimation accuracy, we consider four different measures: (i) root mean squared error, $\text{RMSE}=n^{-1/2}{\left\{\sum_{i=1}^{n}\;{\left({\hat{W}}_{i}-W_{i}\right)}^{2}\right\}}^{1/2}$, (ii) mean absolute error, $\text{MAE}=\;n^{-1}\sum_{i=1}^{n}\;\left|{\hat{W}}_{i}-W_{i}\right|$, (iii) peak signal-to-noise ratio, $\text{PSNR}=20{\text{log}}_{10}\left\{\text{max}\left(W_{i}\right)/\text{RMSE}\right\}$, and (iv) mean relative absolute error, $\text{MRAE}=n^{-1}\sum_{i=1}^{n}\;\left|{\hat{W}}_{i}-W_{i}\right|/W_{i}$. The results of the TPST method are compared to those of other smoothing techniques, such as thin plate smoothing and tensor product spline. Similar to simulation studies in previous sections, we implement the tensor product spline and the thin plate spline smoothing using the function “gam” in R package “mgcv”. Since the thin plate and tensor product methods could be affected by the noises outside the actual brain, we calculate all the measurements based on the voxels within the brain domain to ensure a fair comparison. The estimation and numerosity reduction results of different methods are shown in Table 2.

If a lower target for data reduction is acceptable, smoothing methods can achieve higher levels of accuracy. However, this comes at the cost of a larger basis expansion size. Conventional thin plate and tensor product splines, due to their specific functional form, may result in a high-dimensional representation of the data, particularly in the presence of significant noise or random fluctuations. This can increase the risk of overfitting, as the model may become overly complex and struggle to accurately capture the underlying patterns in the data. Additionally, the high dimensionality can result in memory constraints and make it difficult to process larger data sets. The TPST approach, however, addresses these limitations by providing better control over local variations in the data, resulting in a more compact representation compared to thin plate and tensor product splines. In this example, we cannot implement the thin plate and tensor product splines methods when the dimension of the spline basis exceeds 2500 on a regular PC. As demonstrated in Table 2, the TPST method, in contrast, is better equipped to deal with data with complex shapes or patterns due to its unique combination of sparsity, local control, adaptivity, and computational efficiency.

## 7 Conclusions and Discussion

Challenges in handling and analyzing the irregularly-shaped 3D point cloud data motivated the advanced statistical methods to uncover the underlying trajectory over the point clouds. Unlike conventional smoothing methods, the proposed TPST methods are proven to perform well for complex objects. Moreover, the proposed approaches are able to handle the irregularly and regularly missing data problem, effectively denoise or deblur the data while preserving inherent geometric features or spatial structures, and providing multi-resolution reconstruction. The experimental results demonstrate the effectiveness of the proposed approaches compared to existing smoothing techniques.

Modern geoinformation technologies have provided a variety of feasible means for generating point clouds with billions of points. The enormous size of point cloud data always leads to serious time and memory consumption problems in data processing, storage, visualization, and transmission. Two approaches are currently considered the most promising ones: parallel computing and data reduction. As a numerosity reduction method, the proposed method is an efficient data reduction technique that replaces the original big cloud point with a much smaller form of data representation. On the other hand, parallel computing is very appealing to address such “big” data issues. Unlike tensor product spline or thin plate spline, one unique feature of the proposed TPST is its great scalability in computing. Specifically, the spline basis function is generated restricted to each tetrahedron without any overlap, and the smoothness is achieved only by the constraints on the spline coefficients. Based on this feature, we plan to develop a parallel computing algorithm based on domain decomposition in future work. We will extend the ideas in Lai and Schumaker (2009) for the 2D setting to the 3D setting.

## Supplementary Material

1

## Figures and Tables

**Table 1:** The number of tetrahedra (vertices) in each triangulation.

$\Omega_{1}:\Delta_{11}$	$\Omega_{1}:\Delta_{12}$	$\Omega_{2}:\Delta_{21}$	$\Omega_{2}:\Delta_{22}$
240 (120)	456 (180)	504 (207)	756 (276)

**Table 2:** Estimation and numerosity reduction results.

Method	RMSE	MAE	PSNR	MRAE	Dimension
Thin Plate	0.0906	0.0706	26.2514	0.0917	2000
Tensor Product	0.0945	0.0740	25.8799	0.0957	2197
TPST (d=5, r=1)	0.0860	0.0656	26.7026	0.0877	2212
TPST (d=4, r=0)	0.0647	0.0490	29.1734	0.0652	4247
TPST (d=6, r=1)	0.0539	0.0405	30.7608	0.0522	4859
TPST (d=5, r=0)	0.0461	0.0339	32.1176	0.0444	7941
