# Supplementary material for: Nonparametric Regression for 3D Point Cloud Learning
Source: J Mach Learn Res. Author manuscript; Available in PMC 2024 Oct 10. (PMC11465206)
Supplement: 1 [file NIHMS1977730-supplement-1.pdf]

Appendix A. Detailed Introduction of Trivariate Splines on Triangulation

In this section, we give a detailed discussion of the trivariate splines on tetrahedra, along with an introduction to some theoretical properties. In Section A.1, we first introduce the barycentric coordinates associated with a tetrahedron and show that a trivariate polynomial can be written in a convenient form using the barycentric coordinates. In Section A.2, we describe the directional derivatives of a polynomial, and smoothness conditions for polynomials on adjoining triangular faces. The introduction of trivariate splines on a triangulation is given in Section 2.3 in the main part. Section A.3 illustrates an example of the constraint matrix. The technical details of the conclusions in Section A.2 were given in Section A.4. Sections A.5 and A.6 give a detailed introduction and induction for the directional derivatives for basis functions and penalty matrix, respectively.

A.1 Barycentric Coordinates and Bernstein Basis Polynomials

Given a tetrahedron $T = \langle \boldsymbol{v}_1, \boldsymbol{v}_2, \boldsymbol{v}_3, \boldsymbol{v}_4 \rangle$, any fixed point $\boldsymbol{p} := (x, y, z) \in \mathbb{R}^3$ has a unique representation in terms of $\langle \boldsymbol{v}_1, \boldsymbol{v}_2, \boldsymbol{v}_3, \boldsymbol{v}_4 \rangle$,

$$\boldsymbol{p} = b_1\boldsymbol{v}_1 + b_2\boldsymbol{v}_2 + b_3\boldsymbol{v}_3 + b_4\boldsymbol{v}_4, \quad \text{with } b_1 + b_2 + b_3 + b_4 = 1,$$

where (b_1, b_2, b_3, b_4) are called the *barycentric coordinates* of $\boldsymbol{p}$ *relative to the tetrahedron* T. When the point $\boldsymbol{p}$ is inside or on the faces of T, all b_1, b_2, b_3 and b_4 are nonnegative. By Cramer's rule, the barycentric coordinate corresponding to vertex $\boldsymbol{v}_\imath$, satisfies $b_\imath = \det(\mathbf{M}_\imath)/\det(\mathbf{M})$, $\imath = 1, \ldots, 4$, where

$$\mathbf{M} := \begin{pmatrix} 1 & 1 & 1 & 1 \\ x_1 & x_2 & x_3 & x_4 \\ y_1 & y_2 & y_3 & y_4 \\ z_1 & z_2 & z_3 & z_4 \end{pmatrix}$$

and $\mathbf{M}_\imath$ replaces $\mathbf{M}$'s $\imath$-th column with $(1\ x\ y\ z)^\top$.

The barycentric coordinates (b_1, b_2, b_3, b_4) also have an interesting geometric interpretation. As shown in Figure A.1, for any $\boldsymbol{p} \in T$, it divides the tetrahedron T to four sub-tetrahedra, T_1, T_2, T_3 and T_4. Notice that $\det(\mathbf{M}) = 6V_T$, then $b_\imath = V_{T_\imath}/V_T$, where $T_\imath$ replace the vertex $\boldsymbol{v}_\imath$ in V_T with $\boldsymbol{p}$, $\imath = 1, \ldots, 4$.

For a nondegenerate tetrahedron T and a point $\boldsymbol{p} \in T$ with barycentric coordinates (b_1, b_2, b_3, b_4), for nonnegative integers i, j, k, l with $i + j + k + l = d$, define *trivariate Bernstein basis polynomial of degree* d *relative to* T as

$$B^{d,T}_{ijkl}(\boldsymbol{p}) := \frac{d!}{i!j!k!l!} b_1^i b_2^j b_3^k b_4^l, \text{ with } i + j + k + l = d.$$

For any positive integer d and tetrahedron T, let $\mathcal{P}_d(T)$ be the space of all trivariate polynomials defined on T with degrees less than or equal to d. Note that the dimension of $\mathcal{P}_d(T)$ is $\binom{d+3}{3}$.

According to Theorem 15.8 in Lai and Schumaker (2007) and Lemma A.6 in Appendix Section A.4.1, the set of Bernstein basis polynomials

$$\boldsymbol{B}^d_T(\boldsymbol{p}) := \{B^{d,T}_{ijkl}(\boldsymbol{p}) : i, j, k, l \geq 0, i + j + k + l = d\}$$

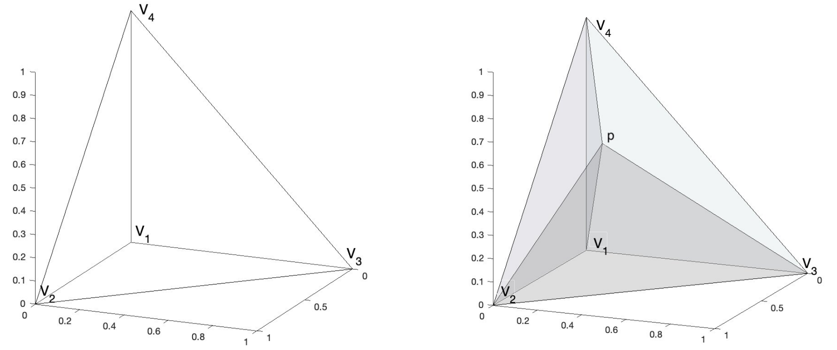

Figure A.1: An illustration of barycentric coordinates of a point $\boldsymbol{p}$ in a tetrahedron T.

forms a basis for the space of polynomials $\mathcal{P}_d(T)$. In addition, Bernstein basis functions $\{B^{d,T}_{ijkl}(\boldsymbol{p})\}_{i+j+k+l=d}$ have the following properties:

1. $\{B^{d,T}_{ijkl}\}$ form a partition of unity, that is, for all $\boldsymbol{p} \in T$, $\sum_{i+j+k+l=d} B^{d,T}_{ijkl}(\boldsymbol{p}) = 1$;

2. for all $\boldsymbol{p} \in T$, $0 \leq B^{d,T}_{ijkl}(\boldsymbol{p}) \leq 1$;

3. $B^{d,T}_{ijkl}$ has a unique maximum at the point $d^{-1}(i\boldsymbol{v}_1 + j\boldsymbol{v}_2 + k\boldsymbol{v}_3 + l\boldsymbol{v}_4)$.

Remark A.1 *Barycentric coordinates are invariant to linear transformations of Cartesian coordinates, that is, they do not depend on the orientation or location of the tetrahedra. Consequently, the trivariate splines based on Bernstein basis polynomials, which are constructed with barycentric coordinates, are also invariant to linear transformations.*

In order to further illustrate the Bernstein basis functions, we present an example of $\{B^{d,T}_{ijkl}(\boldsymbol{p})\}_{i+j+k+l=d}$ for $d = 4$ in Figure A.2. In this example, there are $\binom{7}{3} = 35$ basis functions in total. Note that all the function values vary between 0 and 1, and the colors scale to the quantiles of function values.

Thus, given Bernstein basis functions $\{B^{d,T}_{ijkl}(\boldsymbol{p})\}_{i+j+k+l=d}$, any polynomial $\phi(\boldsymbol{p}) \in \mathcal{P}_d(T)$ can be written uniquely as *B-form*,

$$\phi(\boldsymbol{p})|_T = \sum_{i+j+k+l=d} \gamma_{T;ijkl} B^{d,T}_{ijkl}(\boldsymbol{p}) = \boldsymbol{B}^d_T(\boldsymbol{p})^\top \boldsymbol{\gamma}_T, \tag{A.1}$$

where the coefficients $\boldsymbol{\gamma}_T = \{\gamma_{T;ijkl}\}_{i+j+k+l=d}$ are called *B-coefficients* of ϕ. For the purpose of computer implementation, in this paper, we employ the lexicographical order for ordering of the coefficients $\boldsymbol{\gamma}_T$. To be specific, $\gamma_{T;ijkl}$ orders ahead of $\gamma_{T;i'j'k'l'}$ either (i) $i > i'$, or (ii) $i = i'$ and $j > j'$, or (iii) $i = i'$, $j = j'$ and $k > k'$, or (iv) $i = i'$, $j = j'$, $k = k'$ and $l > l'$. Consequently, we can express

$$\boldsymbol{B}^d_T(\boldsymbol{p}) = \left(B^{d,T}_{d,0,0,0}(\boldsymbol{p}), B^{d,T}_{d-1,1,0,0}(\boldsymbol{p}), B^{d,T}_{d-1,0,1,0}(\boldsymbol{p}), \ldots, B^{d,T}_{0,0,1,d-1}(\boldsymbol{p}), B^{d,T}_{0,0,0,d}(\boldsymbol{p})\right)^\top$$

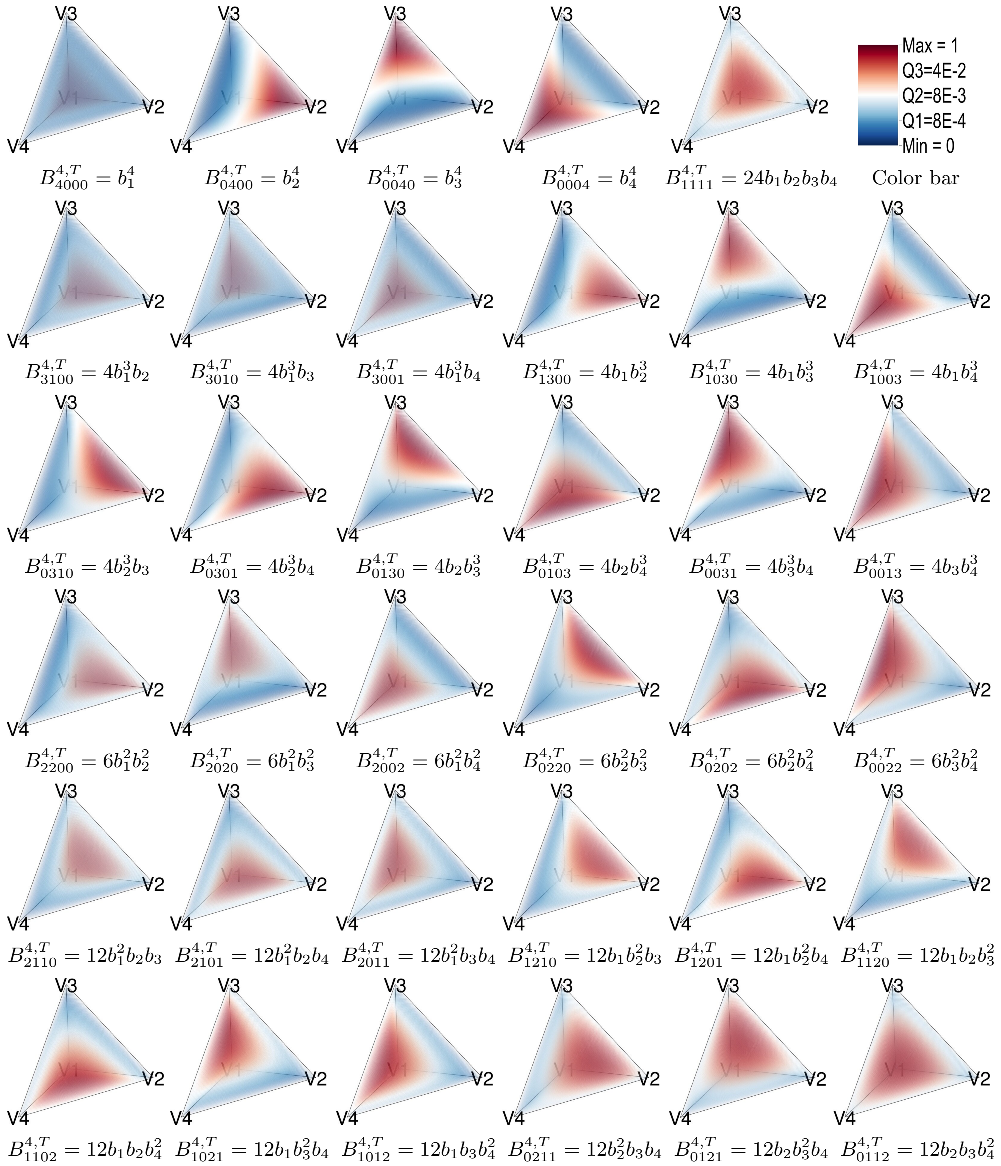

Figure A.2: An illustration of Bernstein basis functions $\{B^{4,T}_{ijkl}(\boldsymbol{p})\}$.

and

$$\boldsymbol{\gamma}_T = \left(\gamma_{T;d,0,0,0}, \gamma_{T;d-1,1,0,0}, \gamma_{T;d-1,0,1,0}, \ldots, \gamma_{T;0,0,1,d-1}, \gamma_{T;0,0,0,d}\right)^\top . \tag{A.2}$$

Accordingly, in Equation A.2, the index of the element $\gamma_{T;ijkl}$ in the vector $\boldsymbol{\gamma}_T$ is:

$$\sum_{m=0}^{d-i} \frac{(m+1)m}{2} + \sum_{n=0}^{d-i-j} (n+1) - k.$$

Note that using a different ordering method will not affect the evaluation results for the trivariate polynomial functions.

It is convenient to derive conditions of continuous connection for polynomials defined on adjacent tetrahedra in using barycentric coordinates and Bernstein basis polynomials. We use the following Example A.1 for illustration.

Example A.1 *Two adjacent tetrahedra* $T = \langle \boldsymbol{v}_2, \boldsymbol{v}_1, \boldsymbol{v}_3, \boldsymbol{v}_4 \rangle$ *and* $\widetilde{T} = \langle \boldsymbol{v}_5, \boldsymbol{v}_1, \boldsymbol{v}_4, \boldsymbol{v}_3 \rangle$ *share a common triangular face* $F = \langle \boldsymbol{v}_1, \boldsymbol{v}_3, \boldsymbol{v}_4 \rangle$*, as illustrated in Figure A.3, where the Cartesian coordinates of the five vertices are* $\boldsymbol{v}_1 = (0,0,0)$*,* $\boldsymbol{v}_2 = (1,0,0)$*,* $\boldsymbol{v}_3 = (0,1,0)$*,* $\boldsymbol{v}_4 = (0,0,1)$*,* $\boldsymbol{v}_5 = (-1,0,0)$*, respectively.*

Denote two sets of Bernstein polynomial basis defined on T and $\widetilde{T}$ using the barycentric coordinates as $\{B^{d,T}_{ijkl}(\boldsymbol{p})\}_{i+j+k+l=d}$ and $\{\widetilde{B}^{d,\widetilde{T}}_{ijkl}(\widetilde{\boldsymbol{p}})\}_{i+j+k+l=d}$, respectively. Consider two degree-d polynomials $\phi(\boldsymbol{p})$ and $\widetilde{\phi}(\widetilde{\boldsymbol{p}})$ defined on T and $\widetilde{T}$, respectively, with B-forms

$$\phi(\boldsymbol{p}) = \sum_{i+j+k+l=d} \gamma_{ijkl} B^{d,T}_{ijkl}(\boldsymbol{p}), \qquad \widetilde{\phi}(\widetilde{\boldsymbol{p}}) = \sum_{i+j+k+l=d} \widetilde{\gamma}_{ijkl} \widetilde{B}^{d,\widetilde{T}}_{ijkl}(\widetilde{\boldsymbol{p}}).$$

For point $\boldsymbol{p}_F \in F$, the barycentric coordinates with respect to T and $\widetilde{T}$ are $(0, b_1, b_3, 1 - b_1 - b_3)$ and $(0, b_1, 1 - b_1 - b_3, b_3)$, respectively. Accordingly, we have

$$\begin{aligned} \phi(\boldsymbol{p}_F) &= \sum_{j+k+l=d} \gamma_{0jkl} \frac{d!}{j!k!l!} b_1^j b_3^k (1 - b_1 - b_3)^l, \\ \widetilde{\phi}(\boldsymbol{p}_F) &= \sum_{j+k+l=d} \widetilde{\gamma}_{0jkl} \frac{d!}{j!k!l!} b_1^j (1 - b_1 - b_3)^k b_3^l. \end{aligned} \tag{A.3}$$

Therefore, ϕ and $\widetilde{\phi}$ are continuous on F if and only if

$$\gamma_{0jkl} = \widetilde{\gamma}_{0jlk} \tag{A.4}$$

for $j, k, l \geq 0$ and $j + k + l = d$.

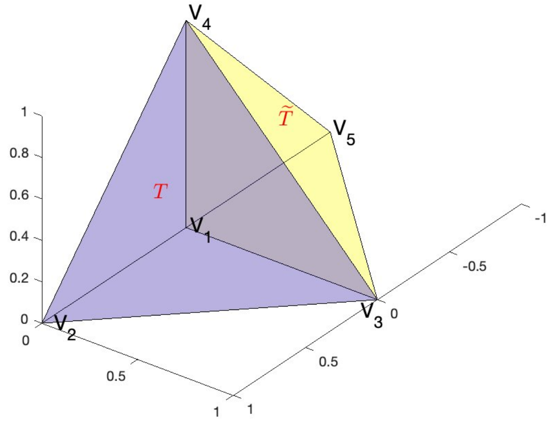

Figure A.3: An example of two tetrahedra sharing a common face.

A.2 Directional Derivatives and Smoothness

To generalize the smoothness restriction over the joint triangular face for two adjacent tetrahedra, we need to introduce the definitions of directional derivative first. Recall that for a general multivariate smooth function ϕ, the directional derivative at point $\boldsymbol{p}$ with respect to direction $\boldsymbol{u}$ is defined as

$$D_{\boldsymbol{u}}\phi(\boldsymbol{p}) := \frac{\partial}{\partial t}\phi(\boldsymbol{p}+t\boldsymbol{u})\Big|_{t=0} = \lim_{t\to 0}\frac{\phi(\boldsymbol{p}+t\boldsymbol{u})-\phi(\boldsymbol{p})}{t}.$$

Accordingly, for vector $\boldsymbol{u} := (u_x, u_y, u_z) \in \mathbb{R}^3$ and trivariate function ϕ, the directional derivative at $\boldsymbol{p} = (x, y, z)$ is

$$D_{\boldsymbol{u}}\phi(x,y,z) := \frac{\partial}{\partial t}\phi(x+tu_x, y+tu_y, z+tu_z)\Big|_{t=0}.$$

Remark A.2 *If ϕ is a polynomial of degree d, by calculus, $D_{\boldsymbol{u}}\phi(x,y,z) = u_x D_x\phi(x,y,z) + u_y D_y\phi(x,y,z) + u_z D_z\phi(x,y,z)$, so $D_{\boldsymbol{u}}\phi$ is a polynomial of degree $d-1$.*

Consider direction $\boldsymbol{u} = \boldsymbol{p}_1 - \boldsymbol{p}_2$, where for $\jmath = 1, 2$, $\boldsymbol{p}_\jmath \in \mathbb{R}^3$ have barycentric coordinates $(b_{\jmath 1}, b_{\jmath 2}, b_{\jmath 3}, b_{\jmath 4})$ with respect to T. Then $\boldsymbol{u}$ is uniquely described by the *directional coordinates* $\boldsymbol{a} = (a_1, a_2, a_3, a_4) = (b_{11} - b_{21}, b_{12} - b_{22}, b_{13} - b_{23}, b_{14} - b_{24})$. Obviously, $a_1 + a_2 + a_3 + a_4 = 0$. Direct calculation gives the directional derivative of the Bernstein basis polynomial B^d_{ijkl}.

Lemma A.3 *(Lemma 15.12 in Lai and Schumaker, 2007) Consider direction $\boldsymbol{u}$ with directional coordinates $\boldsymbol{a} = (a_1, a_2, a_3, a_4)$. Then*

$$D_{\boldsymbol{u}}B^d_{ijkl}(\boldsymbol{p}) = d\left\{a_1 B^{d-1}_{i-1,j,k,l}(\boldsymbol{p}) + a_2 B^{d-1}_{i,j-1,k,l}(\boldsymbol{p}) + a_3 B^{d-1}_{i,j,k-1,l}(\boldsymbol{p}) + a_4 B^{d-1}_{i,j,k,l-1}(\boldsymbol{p})\right\},$$

for any $\boldsymbol{p} \in T$ and $i + j + k + l = d$.

Consequently, one can obtain the directional derivative for any trivariate polynomial ϕ.

Theorem A.4 *(Theorems 15.13 and 15.14 in Lai and Schumaker, 2007) Consider direction $\boldsymbol{u}$ with directional coordinates $\boldsymbol{a} = (a_1, a_2, a_3, a_4)$. Then for any trivariate polynomial ϕ with B-form in Equation A.1, the directional derivative is*

$$D_{\boldsymbol{u}}\phi(\boldsymbol{p}) = d\sum_{i+j+k+l=d-1}\gamma^{(1)}_{ijkl}(\boldsymbol{a})B^{d-1}_{ijkl}(\boldsymbol{p}), \tag{A.5}$$

where $\gamma^{(1)}_{ijkl}(\boldsymbol{a}) = a_1\gamma_{i+1,j,k,l} + a_2\gamma_{i,j+1,k,l} + a_3\gamma_{i,j,k+1,l} + a_4\gamma_{i,j,k,l+1}$.

In general, given $\boldsymbol{u}_1, \ldots, \boldsymbol{u}_m$ with associated directional coordinates $\boldsymbol{a}^{(\imath)} = (a^{(\imath)}_1, a^{(\imath)}_2, a^{(\imath)}_3, a^{(\imath)}_4)$, $\imath = 1, \ldots, m$,

$$D_{\boldsymbol{u}_m}\cdots D_{\boldsymbol{u}_1}\phi(\boldsymbol{p}) = \frac{d!}{(d-m)!}\sum_{i+j+k+l=d-m}\gamma^{(m)}_{ijkl}(\boldsymbol{a}^{(1)},\ldots,\boldsymbol{a}^{(m)})B^{d-m}_{ijkl}(\boldsymbol{p}), \tag{A.6}$$

where the coefficients are defined recursively as follows:

$$\begin{aligned}\gamma_{ijkl}^{(m)}(\boldsymbol{a}^{(1)},\ldots,\boldsymbol{a}^{(m)}) =& a_1^{(m)}\gamma_{i+1,j,k,l}^{(m-1)}(\boldsymbol{a}^{(1)},\ldots,\boldsymbol{a}^{(m-1)}) + a_2^{(m)}\gamma_{i,j+1,k,l}^{(m-1)}(\boldsymbol{a}^{(1)},\ldots,\boldsymbol{a}^{(m-1)})\\ &+ a_3^{(m)}\gamma_{i,j,k+1,l}^{(m-1)}(\boldsymbol{a}^{(1)},\ldots,\boldsymbol{a}^{(m-1)}) + a_4^{(m)}\gamma_{i,j,k,l+1}^{(m-1)}(\boldsymbol{a}^{(1)},\ldots,\boldsymbol{a}^{(m-1)}),\end{aligned}$$

for $m = 1,\ldots,d$, *with* $\gamma_{ijkl}^{(0)}(\boldsymbol{a}) = \gamma_{ijkl}$.

In preparation for the discussion of trivariate spline and spline spaces, we need the following conditions for a smooth join between two polynomial on adjoining tetrahedra, like Example A.1 illustrated in Figure A.3.

Theorem A.5 *Suppose* $\{\gamma_{ijkl}\}$ *and* $\{\widetilde{\gamma}_{ijkl}\}$ *are B-coefficients of* ϕ *and* $\widetilde{\phi}$ *relative to two tetrahedra* $T = \langle \boldsymbol{v}_2,\boldsymbol{v}_1,\boldsymbol{v}_3,\boldsymbol{v}_4\rangle$ *and* $\widetilde{T} = \langle \boldsymbol{v}_5,\boldsymbol{v}_1,\boldsymbol{v}_4,\boldsymbol{v}_3\rangle$, *respectively, where* T *and* $\widetilde{T}$ *share a common face* $F = \langle \boldsymbol{v}_1,\boldsymbol{v}_3,\boldsymbol{v}_4\rangle$. *Then the following statements are equivalent:*

1. ϕ *and* $\widetilde{\phi}$ *join together with* $\mathcal{C}^r$ *continuity across the face* F*;*

2. *For all* $\boldsymbol{p}_F \in F$, $m = 0,\ldots,r$ *and for all directions* $\boldsymbol{u}$,

$$D_{\boldsymbol{u}}^m\phi(\boldsymbol{p}_F) = D_{\boldsymbol{u}}^m\widetilde{\phi}(\boldsymbol{p}_F);$$

3. *For* ϕ *and* $\widetilde{\phi}$ *with B-forms in Equation A.3, for* $i+j+k = d-m$, $m = 0,\ldots,r$,

$$\widetilde{\gamma}_{mijk} = \sum_{\nu+\mu+\kappa+\delta=m} \gamma_{\nu,i+\mu,k+\kappa,j+\delta} B_{\nu\mu\kappa\delta}^m(\boldsymbol{v}_5). \tag{A.7}$$

Consider the case of $d = 2$ piecewise polynomial. There are in total 10 Bernstein basis polynomials with coefficients

$$\begin{aligned}\{\gamma_{ijkl}\} &= (\gamma_{2000},\gamma_{1100},\gamma_{1010},\gamma_{1001},\gamma_{0200},\gamma_{0110},\gamma_{0101},\gamma_{0020},\gamma_{0011},\gamma_{0002})^\top,\\ \{\widetilde{\gamma}_{ijkl}\} &= (\widetilde{\gamma}_{2000},\widetilde{\gamma}_{1100},\widetilde{\gamma}_{1010},\widetilde{\gamma}_{1001},\widetilde{\gamma}_{0200},\widetilde{\gamma}_{0110},\widetilde{\gamma}_{0101},\widetilde{\gamma}_{0020},\widetilde{\gamma}_{0011},\widetilde{\gamma}_{0002})^\top.\end{aligned} \tag{A.8}$$

Note that the barycentric coordinate of $\boldsymbol{v}_5$ with respect to T is $(2,-1,0,0)$.

If the trivariate polynomial is continuous over the whole region, then applying Equation A.7 for $m = r = 0$ generates

$$\widetilde{\gamma}_{0ijk} = \gamma_{0ikj} B_{0000}^0(\boldsymbol{v}_5) = \gamma_{0ikj}, \qquad i+j+k = d, \tag{A.9}$$

which matches the conclusion in Equation A.4.

Based on the de Casteljau Algorithm (Theorem A.7 in Appendix Section A.4.2), we propose the following computationally efficient algorithm in Algorithm A.1 to calculate the derivatives of $\phi(\boldsymbol{p})$.

Algorithm A.1 Algorithm for the derivatives of $\phi(\boldsymbol{p})$

Input: Polynomial with B-form $\phi(\boldsymbol{p}) = \sum_{i+j+k+l=d} \gamma_{ijkl} B^d_{ijkl}(\boldsymbol{p})$, directions $\boldsymbol{u}_1, \dots, \boldsymbol{u}_m$ with associated directional coordinates $\boldsymbol{a}^{(\imath)} = (a^{(\imath)}_1, a^{(\imath)}_2, a^{(\imath)}_3, a^{(\imath)}_4), \imath = 1, \dots, m$.

Initialization: $\imath := 0$, $\gamma^{(0)}_{ijkl} := \gamma_{ijkl}$.

for $\imath = 1, \dots, m$ **do**

  for $i + j + k + l = d - \imath$ **do**

    Compute

$$\begin{aligned}\gamma^{(\imath)}_{ijkl}(\boldsymbol{a}^{(1)}, \dots, \boldsymbol{a}^{(m)}) =& a^{(\imath)}_1 \gamma^{(\imath-1)}_{i+1,j,k,l}(\boldsymbol{a}^{(1)}, \dots, \boldsymbol{a}^{(\imath-1)}) + a^{(\imath)}_2 \gamma^{(\imath-1)}_{i,j+1,k,l}(\boldsymbol{a}^{(1)}, \dots, \boldsymbol{a}^{(\imath-1)}) \\ &+ a^{(\imath)}_3 \gamma^{(\imath-1)}_{i,j,k+1,l}(\boldsymbol{a}^{(1)}, \dots, \boldsymbol{a}^{(\imath-1)}) + a^{(\imath)}_4 \gamma^{(\imath-1)}_{i,j,k,l+1}(\boldsymbol{a}^{(1)}, \dots, \boldsymbol{a}^{(\imath-1)}).\end{aligned}$$

  end for

end for

Output: $D_{\boldsymbol{u}_m} \cdots D_{\boldsymbol{u}_1} \phi(\boldsymbol{p}) = \frac{d!}{(d-m)!} \sum_{i+j+k+l=d-m} \gamma^{(m)}_{ijkl}(\boldsymbol{a}^{(1)}, \dots, \boldsymbol{a}^{(m)}) B^{d-m}_{ijkl}(\boldsymbol{p})$.

A.3 Example of the Constraint Matrix

We illustrate the construction of the constraint matrix $\mathbf{H}$ using the Example A.1 presented with Figure A.3.

Continue our discussions in Section A.2 and consider the case of $d = 2$ piecewise polynomial. Recall that there are in total 10 Bernstein basis polynomials, with coefficients $\{\gamma_{ijkl}\}$ and $\{\widetilde{\gamma}_{ijkl}\}$ in Equation A.8, and the barycentric coordinate of $\boldsymbol{v}_5$ with respect to T is $(2, -1, 0, 0)$.

As shown in Equation A.9, if the trivariate polynomial is continuous over the whole region, then $\widetilde{\gamma}_{0ijk} = \gamma_{0ikj}$ for $i + j + k = d$. In this case, we can write the constraint matrix $\mathbf{H}$ as

$$\mathbf{H} = \begin{pmatrix} 0&0&0&0&1&0&0&0&0&0&0&0&0&0&-1&0&0&0&0&0 \\ 0&0&0&0&0&1&0&0&0&0&0&0&0&0&0&0&-1&0&0&0 \\ 0&0&0&0&0&0&1&0&0&0&0&0&0&0&0&-1&0&0&0&0 \\ 0&0&0&0&0&0&0&1&0&0&0&0&0&0&0&0&0&0&0&-1 \\ 0&0&0&0&0&0&0&0&1&0&0&0&0&0&0&0&0&0&-1&0 \\ 0&0&0&0&0&0&0&0&0&1&0&0&0&0&0&0&0&-1&0&0 \end{pmatrix}. \tag{A.10}$$

Furthermore, if the trivariate polynomial has continuous first derivatives over the whole region, then Equation A.7 holds for both $m = r = 0$ and $m = 1$. Thus, in addition, we also need for any non-negative integers i, j, k such that $i + j + k = 1$,

$$\begin{aligned}\widetilde{\gamma}_{1ijk} =& \gamma_{1ikj} B^1_{1000}(\boldsymbol{v}_5) + \gamma_{0,i+1,j} B^1_{0100}(\boldsymbol{v}_5) + \gamma_{0,i,k+1,j} B^1_{0010}(\boldsymbol{v}_5) + \gamma_{0,i,k,j+1} B^1_{0001}(\boldsymbol{v}_5) \\ =& 2\gamma_{1ikj} - \gamma_{0,i+1,k,j}.\end{aligned} \tag{A.11}$$

Therefore, for $r = 1$ where the trivariate spline has continuous first derivatives over the whole region, we will obtain the following $\mathbf{H}$

$$\mathbf{H} = \begin{pmatrix} 0&0&0&0&1&0&0&0&0&0&0&0&0&0&-1&0&0&0&0&0 \\ 0&0&0&0&0&1&0&0&0&0&0&0&0&0&0&0&-1&0&0&0 \\ 0&0&0&0&0&0&1&0&0&0&0&0&0&0&0&-1&0&0&0&0 \\ 0&0&0&0&0&0&0&1&0&0&0&0&0&0&0&0&0&0&0&-1 \\ 0&0&0&0&0&0&0&0&1&0&0&0&0&0&0&0&0&0&-1&0 \\ 0&0&0&0&0&0&0&0&0&1&0&0&0&0&0&0&0&-1&0&0 \\ 0&2&0&0&-1&0&0&0&0&0&0&-1&0&0&0&0&0&0&0&0 \\ 0&0&2&0&0&-1&0&0&0&0&0&0&-1&0&0&0&0&0&0&0 \\ 0&0&0&2&0&0&-1&0&0&0&0&0&0&-1&0&0&0&0&0&0 \end{pmatrix}.$$

Here, the first six rows match the form in Equation A.10, and the last three rows correspond to Equation A.11.

A.4 Proof of Theorems in Section A.2

In this section, we provide detailed proofs for the theorems in Section A.2.

A.4.1 Theoretical properties of Bernstein bases

Lemma A.6 *For the Bernstein basis functions $\{B^{d,T}_{ijkl}(\boldsymbol{p})\}_{i+j+k+l=d}$, each $B^{d,T}_{ijkl}$ has a unique maximum at the point $d^{-1}(i\boldsymbol{v}_1 + j\boldsymbol{v}_2 + k\boldsymbol{v}_3 + l\boldsymbol{v}_4)$.*

To show Lemma A.6, we first need to show Lemma A.3.

Proof of Lemma A.3**.** Suppose $\boldsymbol{b} = (b_1, b_2, b_3, b_4)$ are the barycentric coordinates of $\boldsymbol{p}$. Then the barycentric coordinates of $\boldsymbol{p} + t\boldsymbol{u}$ are $(b_1 + ta_1, b_2 + ta_2, b_3 + ta_3, b_4 + ta_4)$. Thus, for $i + j + k + l = d$,

$$B^d_{ijkl}(\boldsymbol{p} + t\boldsymbol{u}) = \frac{d!}{i!j!k!l!}(b_1 + ta_1)^i(b_2 + ta_2)^j(b_3 + ta_3)^k(b_4 + ta_4)^l.$$

Hence,

$$\begin{aligned} D_{\boldsymbol{u}}B^d_{ijkl}(\boldsymbol{p}) &= \frac{\partial}{\partial t}\phi(\boldsymbol{p} + t\boldsymbol{u})\Big|_{t=0} \\ =&\frac{d!}{i!j!k!l!}\left(ia_1b_1^{i-1}b_2^jb_3^kb_4^l + ja_2b_1^ib_2^{j-1}b_3^kb_4^l + ka_3b_1^ib_2^jb_3^{k-1}b_4^l + la_4b_1^ib_2^jb_3^kb_4^{l-1}\right) \\ =&d\left\{a_1B^{d-1}_{i-1,j,k,l}(\boldsymbol{p}) + a_2B^{d-1}_{i,j-1,k,l}(\boldsymbol{p}) + a_3B^{d-1}_{i,j,k-1,l}(\boldsymbol{p}) + a_4B^{d-1}_{i,j,k,l-1}(\boldsymbol{p})\right\}. \end{aligned}$$

■

Proof of Lemma A.6 For $T = \langle \boldsymbol{v}_1, \boldsymbol{v}_2, \boldsymbol{v}_3, \boldsymbol{v}_4\rangle$, the barycentric coordinates for $\boldsymbol{v}_1$, $\boldsymbol{v}_2$, $\boldsymbol{v}_3$ and $\boldsymbol{v}_4$ are $(1, 0, 0, 0)$, $(0, 1, 0, 0)$, $(0, 0, 1, 0)$ and $(0, 0, 0, 1)$, respectively. Accordingly, the directional coordinates of $\boldsymbol{u}_1 = \boldsymbol{v}_1 - \boldsymbol{v}_2$, $\boldsymbol{u}_2 = \boldsymbol{v}_1 - \boldsymbol{v}_3$ and $\boldsymbol{u}_3 = \boldsymbol{v}_1 - \boldsymbol{v}_4$ are $(1, -1, 0, 0)$, $(1, 0, -1, 0)$ and $(1, 0, 0, -1)$, respectively. For $\boldsymbol{p} \in T$ with barycentric coordinates $\boldsymbol{b} = (b_1, b_2, b_3, b_4)$, consider derivatives of $B^d_{ijkl}(\boldsymbol{p})$ with respect to directions $\boldsymbol{u}_1$, $\boldsymbol{u}_2$ and $\boldsymbol{u}_3$, then

$$D_{\boldsymbol{u}_1}B^d_{ijkl}(\boldsymbol{p}) = B^d_{ijkl}(\boldsymbol{p})(ib_1^{-1} - jb_2^{-1}), \qquad D_{\boldsymbol{u}_2}B^d_{ijkl}(\boldsymbol{p}) = B^d_{ijkl}(\boldsymbol{p})(ib_1^{-1} - kb_3^{-1}),$$
$$D_{\boldsymbol{u}_3}B^d_{ijkl}(\boldsymbol{p}) = B^d_{ijkl}(\boldsymbol{p})(ib_1^{-1} - lb_4^{-1}).$$

Setting these equations to zero and combining with $b_1+b_2+b_3+b_4=1$ gives $(b_1,b_2,b_3,b_4)=d^{-1}(i,j,k,l)$. ■

A.4.2 Proof of Theorems A.4 and A.5

Proof of Theorem A.4 For $\phi(\boldsymbol{p})=\sum_{i+j+k+l=d}\gamma_{ijkl}B^d_{ijkl}(\boldsymbol{p})$, by Lemma A.3,

$$\begin{aligned}D_{\boldsymbol{u}}\phi(\boldsymbol{p}) &= \sum_{i+j+k+l=d}\gamma_{ijkl}D_{\boldsymbol{u}}B^d_{ijkl}(\boldsymbol{p})\\ &= \sum_{i+j+k+l=d}\gamma_{ijkl}d\left\{a_1B^{d-1}_{i-1,j,k,l}(\boldsymbol{p})+a_2B^{d-1}_{i,j-1,k,l}(\boldsymbol{p})+a_3B^{d-1}_{i,j,k-1,l}(\boldsymbol{p})+a_4B^{d-1}_{i,j,k,l-1}(\boldsymbol{p})\right\}\\ &= d\sum_{i+j+k+l=d-1}\left(a_1\gamma_{i+1,j,k,l}+a_2\gamma_{i,j+1,k,l}+a_3\gamma_{i,j,k+1,l}+a_4\gamma_{i,j,k,l+1}\right)B^{d-1}_{ijkl}(\boldsymbol{p}).\end{aligned}$$

Thus, Equation A.5 follows. Consequently, one can obtain Equation A.6 by repeatedly applying Equation A.5 for directions $\boldsymbol{u}_1,\ldots,\boldsymbol{u}_m$. ■

To show Theorem A.5, we need the following results.

Theorem A.7 *(Theorems 15.10 in Lai and Schumaker, 2007) Suppose $\phi(\boldsymbol{p})$ is a trivariate polynomial with B-form $\phi(\boldsymbol{p})=\sum_{i+j+k+l=d}\gamma_{ijkl}B^d_{ijkl}(\boldsymbol{p})$. Define $\gamma^{(0)}_{ijkl}:=\gamma_{ijkl}$, $i+j+k+l=d$. Suppose $\boldsymbol{p}$ has barycentric coordinates $\boldsymbol{b}=(b_1,b_2,b_3,b_4)$. Then*

$$\phi(\boldsymbol{p})=\sum_{i+j+k+l=d-m}\gamma^{(m)}_{ijkl}B^{d-m}_{ijkl}(\boldsymbol{p}),$$

where for $m=1,\ldots,d$, $\gamma^{(m)}_{ijkl}$ are computed by the recursion

$$\gamma^{(m)}_{ijkl}=b_1\gamma^{(m-1)}_{i+1,j,k,l}+b_2\gamma^{(m-1)}_{i,j+1,k,l}+b_3\gamma^{(m-1)}_{i,j,k+1,l}+b_4\gamma^{(m-1)}_{i,j,k,l+1}, \tag{A.12}$$

for $i+j+k+l=d-m$.

Remark A.8 *The recursive formula in Equation A.12 is also referred as de Casteljau algorithm. See Section 15.6 in Lai and Schumaker (2007) for more details.*

Lemma A.9 *The coefficients in the recursive formula of de Casteljau algorithm in Equation A.12 are given by*

$$\gamma^{(m)}_{ijkl}=\sum_{i'+j'+k'+l'=m}\gamma_{i+i',j+j',k+k',l+l'}B^m_{i'j'k'l'}(\boldsymbol{p}), \tag{A.13}$$

where $i+j+k+l=d-m$.

Proof Define operators $E_1\gamma_{ijkl}=\gamma_{i+1,j,k,l}$, $E_2\gamma_{ijkl}=\gamma_{i,j+1,k,l}$, $E_3\gamma_{ijkl}=\gamma_{i,j,k+1,l}$ and $E_4\gamma_{ijkl}=\gamma_{i,j,k,l+1}$. Thus, by Equation A.12, for $i+j+k+l=d-m$,

$$\begin{aligned}\gamma^{(m)}_{ijkl} &= (b_1E_1+b_2E_2+b_3E_3+b_4E_4)\,\gamma^{(m-1)}_{ijkl}=(b_1E_1+b_2E_2+b_3E_3+b_4E_4)^m\,\gamma_{ijkl}\\ &= \sum_{i'+j'+k'+l'=m}B^m_{i'j'k'l'}(\boldsymbol{p})E_1^{i'}E_2^{j'}E_3^{k'}E_4^{l'}\gamma_{ijkl}=\sum_{i'+j'+k'+l'=m}\gamma_{i+i',j+j',k+k',l+l'}B^m_{i'j'k'l'}(\boldsymbol{p}).\end{aligned}$$

■

Lemma A.10 *Consdier a trivariate polynomial $\phi(\boldsymbol{p})$ with $\phi(\boldsymbol{p}) = \sum_{i+j+k+l=d} \gamma_{ijkl} B^d_{ijkl}(\boldsymbol{p})$ as its B-form. Then for any $1 \leq n \leq d$, the n-th order directional derivative of ϕ with respect to the direction $\boldsymbol{u} = \boldsymbol{v}_4 - \boldsymbol{v}_2$ is given by*

$$D^n_{\boldsymbol{u}}\phi(\boldsymbol{p}) = \frac{d!}{(d-n)!} \sum_{i+j+k+l=d-n} \gamma^{(n)}_{ijkl} B^{d-n}_{ijkl}(\boldsymbol{p},$$

with

$$\gamma^{(n)}_{ijkl} = \sum_{m=0}^{n} \binom{n}{m} (-1)^m \gamma_{i+m,j,k,l+n-m}, \qquad i+j+k+l = d-n.$$

Proof The directional coordinates of $\boldsymbol{u} = \boldsymbol{v}_4 - \boldsymbol{v}_2$ is $\boldsymbol{a} = (-1, 0, 0, 1)$. By Equation A.13,

$$\begin{aligned}\gamma^{(n)}_{ijkl} &= \sum_{i'+j'+k'+l'=n} \gamma_{i+i',j+j',k+k',l+l'} B^n_{i'j'k'l'}(\boldsymbol{p}) = \sum_{i'+l'=n} \gamma_{i+i',j,k,l+l'} \frac{n!}{i'!l'!}(-1)^{i'} \\ &= \sum_{m=0}^{n} \binom{n}{m} (-1)^m \gamma_{i+m,j,k,l+n-m}, \qquad i+j+k+l = d-n.\end{aligned}$$

■

Proof of Theorem A.5 The equivalence between Statements 1 and 2 is obvious by definition. Thus, we just show the equivalence between Statements 2 and 3.

i) We start to consider when $r = 0$. It is equivalent to consider directions $\widetilde{\boldsymbol{u}}$ along F, where the directional coordinates are $(0, 1 - \widetilde{b}_3 - \widetilde{b}_4, \widetilde{b}_3, \widetilde{b}_4)$ and $(0, 1 - \widetilde{b}_3 - \widetilde{b}_4, \widetilde{b}_4, \widetilde{b}_3)$ with respect to the tetrahedron T and $\widetilde{T}$, respectively. Thus, ϕ and $\widetilde{\phi}$ join continuously along F if and only if

$$\sum_{j+k+l=d} \gamma_{0jkl} \frac{d!}{j!k!l!}(1 - \widetilde{b}_3 - \widetilde{b}_4)^j (\widetilde{b}_3)^k (\widetilde{b}_4)^l = \sum_{j+k+l=d} \widetilde{\gamma}_{0jkl} \frac{d!}{j!k!l!}(1 - \widetilde{b}_3 - \widetilde{b}_4)^j (\widetilde{b}_4)^k (\widetilde{b}_3)^l.$$

That is, $\gamma_{0jkl} = \widetilde{\gamma}_{0jlk}$, with $j + k + l = d$, which matches our conclusion in Equation A.7.

ii) Then we consider for $r > 0$. First note that

$$D^n_{\boldsymbol{u}}\phi(\boldsymbol{p}_F) = D^n_{\boldsymbol{u}}\widetilde{\phi}(\boldsymbol{p}_F) \tag{A.14}$$

holds for any $\boldsymbol{p}_F \in F$, $n = 0, \ldots, r$, if and only if Equation A.14 holds for the direction $\boldsymbol{u} = \boldsymbol{v}_5 - \boldsymbol{v}_3$. It is a fact because by the argument in i), all derivatives of ϕ and $\widetilde{\phi}$ corresponding to the directions $\boldsymbol{v}_3 - \boldsymbol{v}_1$ and $\boldsymbol{v}_4 - \boldsymbol{v}_1$, agree at every point on F. And derivatives in all other directions can be written as linear combinations of $D_{\boldsymbol{u}}$, $D_{\boldsymbol{v}_3 - \boldsymbol{v}_1}$ and $D_{\boldsymbol{v}_4 - \boldsymbol{v}_1}$.

Let $\boldsymbol{b} = (b_1, b_2, b_3, b_4)$ be the barycentric coordinates of $\boldsymbol{v}_5$ relative to the tetrahedron T. Correspondingly, the directional coordinates of $\boldsymbol{u}$ are $\boldsymbol{a} = (b_1, b_2, b_3 - 1, b_4)$ and $\widetilde{\boldsymbol{a}} = (1, 0, 0, -1)$ with respect to T and $\widetilde{T}$, respectively.

By Theorem A.4, for each $0 \leq n \leq r$,

$$\begin{aligned}D^n_{\boldsymbol{u}}\phi(\boldsymbol{p}_F)|_F &= \frac{d!}{(d-n)!} \sum_{j+k+l=d-n} \gamma^{(n)}_{0jkl}(\boldsymbol{a}) B^{d-n}_{0jkl}(\boldsymbol{p}_F), \\ D^n_{\boldsymbol{u}}\widetilde{\phi}(\boldsymbol{p}_F)|_F &= \frac{d!}{(d-n)!} \sum_{j+k+l=d-n} \gamma^{(n)}_{0jkl}(\widetilde{\boldsymbol{a}}) B^{d-n}_{0jkl}(\boldsymbol{p}_F).\end{aligned}$$

Since for points $\boldsymbol{p}_F \in F$, $\widetilde{B}^{d-n}_{0jkl}(\boldsymbol{p}_F) = B^{d-n}_{0jlk}(\boldsymbol{p}_F)$, it follows that Equation A.14 holds if and only if for $j+k+l=d-n$, $n=0,\ldots,r$,

$$\widetilde{\gamma}^{(n)}_{0jkl}(\widetilde{\boldsymbol{a}}) = \gamma^{(n)}_{0jlk}(\boldsymbol{a}). \tag{A.15}$$

By Lemma A.10, for $j+k+l=d-n$,

$$\widetilde{\gamma}^{(n)}_{0jkl}(\widetilde{\boldsymbol{a}}) = \sum_{m=0}^{n}(-1)^{n-m}\binom{n}{m}\widetilde{\gamma}_{m,j,k,d-m-j-k}. \tag{A.16}$$

In another direction, following the proof of Lemma A.9, for $j+l+k=d-n$,

$$\begin{aligned}
\gamma^{(n)}_{0jlk}(\boldsymbol{a}) &= \{b_1E_1 + b_2E_2 + (b_3-1)E_3 + b_4E_4\}^n\,\gamma_{0jlk} \\
&= (b_1E_1 + b_2E_2 + b_3E_3 + b_4E_4 - E_3)^n\,\gamma_{0jlk} \\
&= \sum_{m=0}^{n}(-1)^{n-m}\binom{n}{m}(b_1E_1 + b_2E_2 + b_3E_3 + b_4E_4)^n\,\gamma_{0,j,l+n-m,k} \\
&= \sum_{m=0}^{n}(-1)^{n-m}\binom{n}{m}\gamma^{(m)}_{0,j,d-j-k-m,k}(\boldsymbol{b}).
\end{aligned} \tag{A.17}$$

Combining Equations A.16 and A.17, Equation A.15 holds if and only if for $j+k+l=d-n$ and $n=0,\ldots,r$, $\widetilde{\gamma}_{njkl} = \gamma^{(n)}_{0jlk}(\boldsymbol{b})$. By Lemma A.9,

$$\gamma^{(n)}_{0jlk}(\boldsymbol{b}) = \sum_{i'+j'+k'+l'=n}\gamma_{i',j+j',k+k',l+l'}B^{n}_{i'j'k'l'}(\boldsymbol{v}_5).$$

Thus, Equation A.14 holds if and only if

$$\widetilde{\gamma}_{njkl} = \sum_{i'+j'+k'+l'=n}\gamma_{i',j+j',k+k',l+l'}B^{n}_{i'j'k'l'}(\boldsymbol{v}_5),$$

that is, Equation A.7 follows. ■

A.5 Directional Derivatives for Basis Functions

Based on the conclusion in Equation A.6 in Appendix A, the mth order directional derivatives for all the Bernstein basis functions with degree d can be written as some linear combination of Bernstein basis functions with degree $d-m$. Specifically, for directions $\boldsymbol{u}_1,\ldots,\boldsymbol{u}_m$, there exists a $\binom{d+3}{3}\times\binom{d+3-m}{3}$ matrix $\mathbf{C}^{(m)}_d(\boldsymbol{u}_1,\ldots,\boldsymbol{u}_m)$ such that

$$D_{\boldsymbol{u}_m}\cdots D_{\boldsymbol{u}_1}\mathbf{B}_d(\boldsymbol{p}) = \mathbf{C}^{(m)}_d(\boldsymbol{u}_1,\ldots,\boldsymbol{u}_m)\mathbf{B}_{d-m}(\boldsymbol{p}).$$

Consequently, with $D_{\boldsymbol{u}_{m-1}}\cdots D_{\boldsymbol{u}_1}\mathbf{B}_d(\boldsymbol{p}) = \mathbf{C}^{(m-1)}_d(\boldsymbol{u}_1,\ldots,\boldsymbol{u}_{m-1})\mathbf{B}_{d-m+1}(\boldsymbol{p})$, we have

$$\begin{aligned}
D_{\boldsymbol{u}_m}D_{\boldsymbol{u}_{m-1}}\cdots D_{\boldsymbol{u}_1}\mathbf{B}_d(\boldsymbol{p}) &= \mathbf{C}^{(m-1)}_d(\boldsymbol{u}_1,\ldots,\boldsymbol{u}_{m-1})D_{\boldsymbol{u}_m}\mathbf{B}_{d-m+1}(\boldsymbol{p}) \\
&= \mathbf{C}^{(m-1)}_d(\boldsymbol{u}_1,\ldots,\boldsymbol{u}_{m-1})\mathbf{C}^{(1)}_{d-m+1}(\boldsymbol{u}_m)\mathbf{B}_{d-m}(\boldsymbol{p}),
\end{aligned}$$

which implies $\mathbf{C}_d^{(m)}(\boldsymbol{u}_1,\ldots,\boldsymbol{u}_m) = \mathbf{C}_d^{(m-1)}(\boldsymbol{u}_1,\ldots,\boldsymbol{u}_{m-1})\mathbf{C}_{d-m+1}^{(1)}(\boldsymbol{u}_m)$. Keep decomposing matrix $\mathbf{C}_d^{(m-1)}(\boldsymbol{u}_1,\ldots,\boldsymbol{u}_{m-1})$, we have $\mathbf{C}_d^{(m)}(\boldsymbol{u}_1,\ldots,\boldsymbol{u}_m) = \mathbf{C}_d^{(1)}(\boldsymbol{u}_1)\cdots\mathbf{C}_{d-m+1}^{(1)}(\boldsymbol{u}_m)$.

Therefore, we can obtain the explicit form of the mth order directional derivatives for the Bernstein basis functions with degree d, given the explicit form of matrix $\mathbf{C}_d^{(1)}(\boldsymbol{u})$. Based on Lemma A.3,

$$D_{\boldsymbol{u}} B_{ijkl}^d(\boldsymbol{p}) = d\left\{a_1 B_{i-1,j,k,l}^{d-1}(\boldsymbol{p}) + a_2 B_{i,j-1,k,l}^{d-1}(\boldsymbol{p}) + a_3 B_{i,j,k-1,l}^{d-1}(\boldsymbol{p}) + a_4 B_{i,j,k,l-1}^{d-1}(\boldsymbol{p})\right\},$$

where (a_1,a_2,a_3,a_4) is the barycentric coordinate of direction $\boldsymbol{u}$. Then, the $\{\sum_{m=0}^{d-i}(m+1)m/2 + \sum_{n=0}^{d-i-j}(n+1) - k\}$th row of matrix $\mathbf{C}_d^{(1)}(\boldsymbol{u})$ is $d(a_1\boldsymbol{e}_{\mathcal{I}_1} + a_2\boldsymbol{e}_{\mathcal{I}_2} + a_3\boldsymbol{e}_{\mathcal{I}_3} + a_4\boldsymbol{e}_{\mathcal{I}_4})$, where $\boldsymbol{e}_{\mathcal{I}}$ is unit vector with $\mathcal{I}$th element being one, $\mathcal{I}_1$, $\mathcal{I}_2$, $\mathcal{I}_3$ and $\mathcal{I}_4$ are indexes of basis functions $B_{i-1,j,k,l}^{d-1}(\boldsymbol{p})$, $B_{i,j-1,k,l}^{d-1}(\boldsymbol{p})$, $B_{i,j,k-1,l}^{d-1}(\boldsymbol{p})$, and $B_{i,j,k,l-1}^{d-1}(\boldsymbol{p})$.

In the following, we provide a simple example to illustrate how to calculate the first and second-order derivatives and therefore to construct the penalty matrix $\mathbf{P}_T$ in Equation A.18 in Section A.6. Since the penalty function is calculated through the second-order derivatives in terms of the three-dimensional Cartesian coordinate system $x=(1,0,0)$, $y=(0,1,0)$ and $z=(0,0,1)$, in the following example, we only consider directional derivatives for x,y,z.

Consider the basis functions on the tetrahedron T in the Figure A.1 with $d=3$. The horizontal axis $x=(1,0,0)$ can be written as $x=\boldsymbol{v}_1-\boldsymbol{v}_2$, therefore, the barycentric coordinates of x is $(1,-1,0,0)$. Then, the first and second order derivatives of all the Bernstein basis functions $\mathbf{B}_3(\boldsymbol{p})$ are $D^{(1,0,0)}\mathbf{B}_3(\boldsymbol{p}) = \mathbf{C}_3^{(1)}(1,0,0)\mathbf{B}_2(\boldsymbol{p})$ and $D^{(2,0,0)}\mathbf{B}_3(\boldsymbol{p}) = \mathbf{C}_3^{(2)}(x,x)\mathbf{B}_1(\boldsymbol{p}) = \mathbf{C}_3^{(1)}(x)\mathbf{C}_2^{(1)}(x)\mathbf{B}_1(\boldsymbol{p})$, where matrices $\mathbf{C}_2^{(1)}(x)$ and $\mathbf{C}_3^{(1)}(x)$ are

$$\mathbf{C}_2^{(1)}(x) = \begin{pmatrix} 2 & 0 & 0 & 0 \\ -2 & 2 & 0 & 0 \\ 0 & 0 & 2 & 0 \\ 0 & 0 & 0 & 2 \\ 0 & -2 & 0 & 0 \\ 0 & 0 & -2 & 0 \\ 0 & 0 & 0 & -2 \\ 0 & 0 & 0 & 0 \\ 0 & 0 & 0 & 0 \\ 0 & 0 & 0 & 0 \end{pmatrix}, \quad \mathbf{C}_3^{(1)}(x) = \begin{pmatrix} 3 & 0 & 0 & 0 & 0 & 0 & 0 & 0 & 0 & 0 \\ -3 & 3 & 0 & 0 & 0 & 0 & 0 & 0 & 0 & 0 \\ 0 & 0 & 3 & 0 & 0 & 0 & 0 & 0 & 0 & 0 \\ 0 & 0 & 0 & 3 & 0 & 0 & 0 & 0 & 0 & 0 \\ 0 & -3 & 0 & 0 & 3 & 0 & 0 & 0 & 0 & 0 \\ 0 & 0 & -3 & 0 & 0 & 3 & 0 & 0 & 0 & 0 \\ 0 & 0 & 0 & -3 & 0 & 0 & 3 & 0 & 0 & 0 \\ 0 & 0 & 0 & 0 & 0 & 0 & 0 & 3 & 0 & 0 \\ 0 & 0 & 0 & 0 & 0 & 0 & 0 & 0 & 3 & 0 \\ 0 & 0 & 0 & 0 & 0 & 0 & 0 & 0 & 0 & 3 \\ 0 & 0 & 0 & 0 & -3 & 0 & 0 & 0 & 0 & 0 \\ 0 & 0 & 0 & 0 & 0 & -3 & 0 & 0 & 0 & 0 \\ 0 & 0 & 0 & 0 & 0 & 0 & -3 & 0 & 0 & 0 \\ 0 & 0 & 0 & 0 & 0 & 0 & 0 & -3 & 0 & 0 \\ 0 & 0 & 0 & 0 & 0 & 0 & 0 & 0 & -3 & 0 \\ 0 & 0 & 0 & 0 & 0 & 0 & 0 & 0 & 0 & -3 \\ 0 & 0 & 0 & 0 & 0 & 0 & 0 & 0 & 0 & 0 \\ 0 & 0 & 0 & 0 & 0 & 0 & 0 & 0 & 0 & 0 \\ 0 & 0 & 0 & 0 & 0 & 0 & 0 & 0 & 0 & 0 \\ 0 & 0 & 0 & 0 & 0 & 0 & 0 & 0 & 0 & 0 \end{pmatrix}.$$

A.6 Details of Constructing Penalty Matrix

Next, we introduce the details of constructing penalty $\mathbf{P}$ matrix. Following the definition of penalty matrix $\mathbf{P}_T$ in the Section 3, we can further implement as

$$\begin{aligned}\mathcal{E}(s_T) &= \sum_{|\boldsymbol{\alpha}|=2} \binom{2}{\alpha_1}\binom{2-\alpha_1}{\alpha_2} \int_T \Big\{ \sum_{i+j+k+l=d} \gamma_{T;ijkl} D^{\boldsymbol{\alpha}} B^{d,T}_{ijkl}(\boldsymbol{p}) \Big\}^2 \mathrm{d}\boldsymbol{p} \\ &= \sum_{|\boldsymbol{\alpha}|=2} \binom{2}{\alpha_1}\binom{2-\alpha_1}{\alpha_2} \sum_{i+j+k+l=d}\ \sum_{i'+j'+k'+l'=d} \gamma_{T;ijkl}\gamma_{T;i'j'k'l'} \times \\ &\quad \int_T \Big\{ D^{\boldsymbol{\alpha}} B^{d,T}_{ijkl}(\boldsymbol{p}) \Big\} \Big\{ D^{\boldsymbol{\alpha}} B^{d,T}_{i'j'k'l'}(\boldsymbol{p}) \Big\} \mathrm{d}\boldsymbol{p} \\ &= \sum_{|\boldsymbol{\alpha}|=2} \boldsymbol{\gamma}_T^\top \mathbf{P}^{\boldsymbol{\alpha}}_T \boldsymbol{\gamma}_T = \boldsymbol{\gamma}_T^\top \mathbf{P}_T \boldsymbol{\gamma}_T, \end{aligned} \tag{A.18}$$

where each $\mathbf{P}^{\boldsymbol{\alpha}}_T$ is a $\binom{d+3}{3} \times \binom{d+3}{3}$ matrix with entries $\int_T \{D^{\boldsymbol{\alpha}} B^{d,T}_{ijkl}(\boldsymbol{p})\}\ \{D^{\boldsymbol{\alpha}} B^{d,T}_{i'j'k'l'}(\boldsymbol{p})\}\mathrm{d}\boldsymbol{p}$ for $\boldsymbol{\alpha}$ satisfying $|\boldsymbol{\alpha}| = 2$. Applying the results of directional derivatives in Section A.5, the second order derivative of $\mathbf{B}_d(\boldsymbol{p})$ at directions $\boldsymbol{u}_1$ and $\boldsymbol{u}_2$ are

$$D_{\boldsymbol{u}_2} D_{\boldsymbol{u}_1} \mathbf{B}_d(\boldsymbol{p}) = \mathbf{C}^{(2)}_d(\boldsymbol{u}_1, \boldsymbol{u}_2)\mathbf{B}_{d-2}(\boldsymbol{p}).$$

For notation simplicity, we denote $\mathbf{C}^{\boldsymbol{\alpha}}_d$ the derivative coefficient matrix for $D^{\boldsymbol{\alpha}}\mathbf{B}_d(\boldsymbol{p})$ with respect to direction(s) of Cartesian coordinate system, that is, $D^{\boldsymbol{\alpha}}\mathbf{B}_d(\boldsymbol{p}) = \mathbf{C}^{\boldsymbol{\alpha}}_d \mathbf{B}_{d-|\boldsymbol{\alpha}|}(\boldsymbol{p})$. Therefore, for $\boldsymbol{\alpha}$ with $|\boldsymbol{\alpha}| = 2$, we have

$$\int_T \{D^{\boldsymbol{\alpha}}\mathbf{B}_d(\boldsymbol{p})\} \{D^{\boldsymbol{\alpha}}\mathbf{B}_d(\boldsymbol{p})\}^\top \mathrm{d}\boldsymbol{p} = \mathbf{C}^{\boldsymbol{\alpha}}_d \left\{ \int_T \mathbf{B}_{d-2}(\boldsymbol{p})\mathbf{B}_{d-2}(\boldsymbol{p})^\top \mathrm{d}\boldsymbol{p} \right\} (\mathbf{C}^{\boldsymbol{\alpha}}_d)^\top.$$

By the Lemma 15.29 in Lai and Schumaker (2007), we have

$$\begin{aligned}\int_T B^{d-2,T}_{\nu\mu\kappa\delta}(\boldsymbol{p}) B^{d-2,T}_{\nu'\mu'\kappa'\delta'}(\boldsymbol{p})\mathrm{d}\boldsymbol{p} &= \frac{\binom{\nu+\nu'}{\nu}\binom{\mu+\mu'}{\mu}\binom{\kappa+\kappa'}{\kappa}\binom{\delta+\delta'}{\delta}}{\binom{2d-4}{d-2}} \int_T B^{2d-4,T}_{\nu+\nu',\mu+\mu',\kappa+\kappa',\delta+\delta'}(\boldsymbol{p})\mathrm{d}\boldsymbol{p} \\ &= \frac{\binom{\nu+\nu'}{\nu}\binom{\mu+\mu'}{\mu}\binom{\kappa+\kappa'}{\kappa}\binom{\delta+\delta'}{\delta}}{\binom{2d-4}{d-2}\binom{2d-1}{3}} V_T,\end{aligned}$$

recall that V_T is the volume of tetrahedron T. Let $\mathbf{L}^{d-2}_T = \int_T \mathbf{B}_{d-2}(\boldsymbol{p})\mathbf{B}_{d-2}(\boldsymbol{p})^\top \mathrm{d}\boldsymbol{p}$ be the $\binom{d+1}{3} \times \binom{d+1}{3}$ matrix with entries $\int_T B^{d-2,T}_{\nu\mu\kappa\delta}(\boldsymbol{p}) B^{d-2,T}_{\nu'\mu'\kappa'\delta'}(\boldsymbol{p})\mathrm{d}\boldsymbol{p}$. We finally obtain $\mathbf{P}^{\boldsymbol{\alpha}}_T = \mathbf{C}^{\boldsymbol{\alpha}}_d \mathbf{L}^{d-2}_T (\mathbf{C}^{\boldsymbol{\alpha}}_d)^\top$ and $\mathbf{P}_T = \sum_{|\boldsymbol{\alpha}|=2} \mathbf{P}^{\boldsymbol{\alpha}}_T$. Consequently, $\mathcal{E}(s) = \boldsymbol{\gamma}^\top \mathbf{P} \boldsymbol{\gamma}$.

Following the same example in Section A.5, similarly, the barycentric coordinates of $y = \boldsymbol{v}_3 - \boldsymbol{v}_2$ and $z = \boldsymbol{v}_4 - \boldsymbol{v}_2$ are $(0, -1, 1, 0)$ and $(0, -1, 0, 1)$, respectively. Following similar inductions, the second directional derivative of the Bernstein basis polynomials are the linear combination of $\{B^{1,T}_{1000}(\boldsymbol{p}), B^{1,T}_{0100}(\boldsymbol{p}), B^{1,T}_{0010}(\boldsymbol{p}), B^{1,T}_{0001}(\boldsymbol{p})\}$. For example, given the formulae of $\mathbf{C}^{(1)}_2$ and $\mathbf{C}^{(1)}_3$ shown above, we have $D^{(1,0,0)} B^{3,T}_{2100}(\boldsymbol{p}) = 3B^{2,T}_{1100}(\boldsymbol{p}) - 3B^{2,T}_{2000}(\boldsymbol{p})$, and $D^{(2,0,0)} B^{3,T}_{2100}(\boldsymbol{p}) = -12B^{1,T}_{1000}(\boldsymbol{p}) + 6B^{1,T}_{0100}(\boldsymbol{p})$. According to the results in Sections A.5

and A.6, the coefficient matrices for second order derivatives are $\mathbf{C}_3(2,0,0)$, $\mathbf{C}_3(0,2,0)$, $\mathbf{C}_3(0,0,2)$, $\mathbf{C}_3(1,1,0)$, $\mathbf{C}_3(1,0,1)$, and $\mathbf{C}_3(0,1,1)$:

$$
\overset{\mathbf{C}_3(2,0,0)}{\begin{pmatrix}
6 & 0 & 0 & 0 \\
-12 & 6 & 0 & 0 \\
0 & 0 & 6 & 0 \\
0 & 0 & 0 & 6 \\
6 & -12 & 0 & 0 \\
0 & 0 & -12 & 0 \\
0 & 0 & 0 & -12 \\
0 & 0 & 0 & 0 \\
0 & 0 & 0 & 0 \\
0 & 0 & 0 & 0 \\
0 & 6 & 0 & 0 \\
0 & 0 & 6 & 0 \\
0 & 0 & 0 & 6 \\
0 & 0 & 0 & 0 \\
0 & 0 & 0 & 0 \\
0 & 0 & 0 & 0 \\
0 & 0 & 0 & 0 \\
0 & 0 & 0 & 0 \\
0 & 0 & 0 & 0 \\
0 & 0 & 0 & 0
\end{pmatrix}},
\overset{\mathbf{C}_3(0,2,0)}{\begin{pmatrix}
0 & 0 & 0 & 0 \\
0 & 0 & 0 & 0 \\
0 & 0 & 0 & 0 \\
0 & 0 & 0 & 0 \\
6 & 0 & 0 & 0 \\
-12 & 0 & 0 & 0 \\
0 & 0 & 0 & 0 \\
6 & 0 & 0 & 0 \\
0 & 0 & 0 & 0 \\
0 & 0 & 0 & 0 \\
0 & 6 & 0 & 0 \\
0 & -12 & 6 & 0 \\
0 & 0 & 0 & 6 \\
0 & 6 & -12 & 0 \\
0 & 0 & 0 & -12 \\
0 & 0 & 0 & 0 \\
0 & 0 & 6 & 0 \\
0 & 0 & 0 & 6 \\
0 & 0 & 0 & 0 \\
0 & 0 & 0 & 0
\end{pmatrix}},
\overset{\mathbf{C}_3(0,0,2)}{\begin{pmatrix}
0 & 0 & 0 & 0 \\
0 & 0 & 0 & 0 \\
0 & 0 & 0 & 0 \\
0 & 0 & 0 & 0 \\
6 & 0 & 0 & 0 \\
0 & 0 & 0 & 0 \\
-12 & 0 & 0 & -12 \\
0 & 0 & 0 & 0 \\
0 & 0 & 0 & 0 \\
6 & 0 & 0 & 0 \\
0 & 6 & 0 & 0 \\
0 & 0 & 6 & 0 \\
0 & -12 & 0 & 6 \\
0 & 0 & 0 & 0 \\
0 & 0 & -12 & 0 \\
0 & 6 & 0 & -12 \\
0 & 0 & 0 & 0 \\
0 & 0 & 6 & 0 \\
0 & 0 & 0 & 6 \\
0 & 0 & 0 & 0
\end{pmatrix}},
$$

$$
\overset{\mathbf{C}_3(1,1,0)}{\begin{pmatrix}
0 & 0 & 0 & 0 \\
-6 & 0 & 0 & 0 \\
6 & 0 & 0 & 0 \\
0 & 0 & 0 & 0 \\
6 & -6 & 0 & 0 \\
-6 & 6 & -6 & 0 \\
0 & 0 & 0 & -6 \\
0 & 0 & 6 & 0 \\
0 & 0 & 0 & 6 \\
0 & 0 & 0 & 0 \\
0 & 6 & 0 & 0 \\
0 & -6 & 6 & 0 \\
0 & 0 & 0 & 6 \\
0 & 0 & -6 & 0 \\
0 & 0 & 0 & -6 \\
0 & 0 & 0 & 0 \\
0 & 0 & 0 & 0 \\
0 & 0 & 0 & 0 \\
0 & 0 & 0 & 0 \\
0 & 0 & 0 & 0
\end{pmatrix}},
\overset{\mathbf{C}_3(1,0,1)}{\begin{pmatrix}
0 & 0 & 0 & 0 \\
-6 & 0 & 0 & 0 \\
0 & 0 & 0 & 0 \\
6 & 0 & 0 & 0 \\
6 & -6 & 0 & 0 \\
0 & 0 & -6 & 0 \\
-6 & 6 & 0 & -6 \\
0 & 0 & 0 & 0 \\
0 & 0 & 6 & 0 \\
0 & 0 & 0 & 6 \\
0 & 6 & 0 & 0 \\
0 & 0 & 6 & 0 \\
0 & -6 & 0 & 6 \\
0 & 0 & 0 & 0 \\
0 & 0 & -6 & 0 \\
0 & 0 & 0 & -6 \\
0 & 0 & 0 & 0 \\
0 & 0 & 0 & 0 \\
0 & 0 & 0 & 0 \\
0 & 0 & 0 & 0
\end{pmatrix}},
\overset{\mathbf{C}_3(0,1,1)}{\begin{pmatrix}
0 & 0 & 0 & 0 \\
0 & 0 & 0 & 0 \\
0 & 0 & 0 & 0 \\
0 & 0 & 0 & 0 \\
6 & 0 & 0 & 0 \\
-6 & 0 & 0 & 0 \\
-6 & 0 & 0 & 0 \\
0 & 0 & 0 & 0 \\
6 & 0 & 0 & 0 \\
0 & 0 & 0 & 0 \\
0 & 6 & 0 & 0 \\
0 & -6 & 6 & 0 \\
0 & -6 & 0 & 6 \\
0 & 0 & -6 & 0 \\
0 & 6 & -6 & -6 \\
0 & 0 & 0 & -6 \\
0 & 0 & 0 & 0 \\
0 & 0 & 0 & 0 \\
0 & 0 & 0 & 0 \\
0 & 0 & 0 & 0
\end{pmatrix}}.
$$

Therefore, we have

$$\mathbf{L}_T^1 = \int_T \mathbf{B}_1(\boldsymbol{p})\mathbf{B}_1(\boldsymbol{p})^\top \mathrm{d}\boldsymbol{p} = \frac{1}{12}\begin{pmatrix} 2 & 1 & 1 & 1 \\ 1 & 2 & 1 & 1 \\ 1 & 1 & 2 & 1 \\ 1 & 1 & 1 & 2 \end{pmatrix}, \text{ and } \mathbf{P}_T = \sum_{|\boldsymbol{\alpha}|=2} \mathbf{C}_3(\boldsymbol{\alpha})\mathbf{L}_T^1\{\mathbf{C}_3(\boldsymbol{\alpha})\}^\top.$$

Appendix B. Proof of Theoretical Results

B.1 Preliminaries

In this section, we start with the stability condition for trivariate spline bases over triangulations, and establish the uniform rate of the approximation of empirical inner product to the theoretical inner product based on the stability property.

Lemma B.1 *Let $\{B_\xi\}_{\xi\in\mathcal{M}}$ be the basis for $\mathcal{S}_d^r(\triangle)$ constructed in Lai and Schumaker (2007), where $\mathcal{M}$ stands for the index set of spline bases. Then there exist positive constants C_1, C_2 depending on degree d and partition quasi-uniform parameter β such that*

$$C_1|\triangle|^3 \sum_{\xi\in\mathcal{M}} |c_\xi|^2 \leq \left\| \sum_{\xi\in\mathcal{M}} c_\xi B_\xi \right\|_{L^2(\Omega)}^2 \leq C_2|\triangle|^3 \sum_{\xi\in\mathcal{M}} |c_\xi|^2,$$

for all $c_\xi, \xi \in \mathcal{M}$.

Proof The proof of Lemma B.1 follows directly from the Theorem 17.18 in Lai and Schumaker (2007). ■

We illustrate the connection between the theoretical norm and L^2 norm in Lemma B.2.

Lemma B.2 *Let $\{B_\xi\}_{\xi\in\mathcal{M}}$ be the basis for $\mathcal{S}_d^r(\triangle)$ as in Lemma* B.1*. Under Assumption (A3), if $d \geq 6r+3$ and $\triangle$ is a β-quasi-uniform triangulation, there exist positive constants C_1, C_2 such that*

$$C_1 \left\| \sum_{\xi\in\mathcal{M}} c_\xi B_\xi \right\|_{L^2(\Omega)}^2 \leq \left\| \sum_{\xi\in\mathcal{M}} c_\xi B_\xi \right\|_{\Omega}^2 \leq C_2 \left\| \sum_{\xi\in\mathcal{M}} c_\xi B_\xi \right\|_{L^2(\Omega)}^2,$$

for all $c_\xi, \xi \in \mathcal{M}$.

Proof It is straightforward to obtain upper and lower bounds of $\|\sum_{\xi\in\mathcal{M}} c_\xi B_\xi\|_\Omega^2$ by Assumption (A3). ■

Proof of Lemma 3. According to the definition of empirical inner product and induced norm of the theoretical L^2 inner product in Section 3,

$$\langle g_1, g_2\rangle_{n,\Omega} = \frac{1}{n}\sum_{i=1}^n \left\{\sum_{\xi\in\mathcal{M}} c_\xi B_\xi(\boldsymbol{p}_i)\right\}\left\{\sum_{\zeta\in\mathcal{M}} \tilde{c}_\zeta B_\zeta(\boldsymbol{p}_i)\right\} = \sum_{\xi,\zeta\in\mathcal{M}} c_\xi \tilde{c}_\zeta \langle B_\xi, B_\zeta\rangle_{n,\Omega},$$

$$\|g_1\|_{\Omega}^2 = \sum_{\xi,\xi'\in\mathcal{M}} c_\xi c_{\xi'} \left\langle B_\xi, B_{\xi'}\right\rangle_\Omega, \text{ and } \|g_2\|_{\Omega}^2 = \sum_{\zeta,\zeta'\in\mathcal{M}} \tilde{c}_\zeta \tilde{c}_{\zeta'} \left\langle B_\zeta, B_{\zeta'}\right\rangle_\Omega.$$

By Lemma B.1, we have for g_1 and g_2, $C_1|\triangle|^3 \sum_{\xi\in\mathcal{M}} |c_\xi|^2 \le \|g_1\|_{L^2(\Omega)}^2 \le C_2|\triangle|^3 \sum_{\xi\in\mathcal{M}} |c_\xi|^2$ and $C_1|\triangle|^3 \sum_{\zeta\in\mathcal{M}} |\tilde{c}_\zeta|^2 \le \|g_2\|_{L^2(\Omega)}^2 \le C_2|\triangle|^3 \sum_{\zeta\in\mathcal{M}} |\tilde{c}_\zeta|^2$. Consequently, by Lemma B.2,

$$C_1|\triangle|^3 \left\{\sum_{\xi\in\mathcal{M}} |c_\xi|^2 \sum_{\zeta\in\mathcal{M}} |\tilde{c}_\zeta|^2\right\}^{1/2} \le \|g_1\|_\Omega \|g_2\|_\Omega \le C_2|\triangle|^3 \left\{\sum_{\xi\in\mathcal{M}} |c_\xi|^2 \sum_{\zeta\in\mathcal{M}} |\tilde{c}_\zeta|^2\right\}^{1/2}.$$

Thus,

$$\begin{aligned} R_n &\le \frac{\sum_{\xi,\zeta\in\mathcal{M}} |c_\xi \tilde{c}_\zeta|}{C_1|\triangle|^3 \left\{\sum_{\xi\in\mathcal{M}} |c_\xi|^2 \sum_{\zeta\in\mathcal{M}} |\tilde{c}_\zeta|^2\right\}^{1/2}} \max_{\xi,\zeta\in\mathcal{M}} \left|\langle B_\xi, B_\zeta\rangle_{n,\Omega} - \langle B_\xi, B_\zeta\rangle_\Omega\right| \\ &\le C_1^{-1} |\triangle|^{-3} \max_{\xi,\zeta\in\mathcal{M}} \left|\langle B_\xi, B_\zeta\rangle_{n,\Omega} - \langle B_\xi, B_\zeta\rangle_\Omega\right|. \end{aligned} \tag{B.1}$$

To obtain the conclusion, it suffices to show that with probability one,

$$\max_{\xi,\zeta\in\mathcal{M}} \left|\langle B_\xi, B_\zeta\rangle_{n,\Omega} - \langle B_\xi, B_\zeta\rangle_\Omega\right| = O_P\left\{(\log n)^{1/2}(nN)^{-1/2}\right\}. \tag{B.2}$$

Let $R_{\xi,\zeta,i} = B_\xi(\boldsymbol{p}_i) B_\zeta(\boldsymbol{p}_i) - \mathrm{E}B_\xi(\boldsymbol{p}_i) B_\zeta(\boldsymbol{p}_i)$. Then we have the second moment $\mathrm{E}R_{\xi,\zeta,i}^2 = \mathrm{E}\{B_\xi^2(\boldsymbol{p}_i) B_\zeta^2(\boldsymbol{p}_i)\} - \{\mathrm{E}B_\xi(\boldsymbol{p}_i) B_\zeta(\boldsymbol{p}_i)\}^2$, where we have $\mathrm{E}\{B_\xi^2(\boldsymbol{p}_i) B_\zeta^2(\boldsymbol{p}_i)\} \sim |\triangle|^3$ and $\{\mathrm{E}B_\xi(\boldsymbol{p}_i) B_\zeta(\boldsymbol{p}_i)\}^2 \sim |\triangle|^6$. Hence, $\mathrm{E}R_{\xi,\zeta,i}^2 \sim |\triangle|^3$. Note that the k-th moment is $\mathrm{E}|R_{\xi,\zeta,i}|^k \le 2^{k-1}\{\mathrm{E}|B_\xi(\boldsymbol{p}_i)B_\zeta(\boldsymbol{p}_i)|^k + |\mathrm{E}B_\xi(\boldsymbol{p}_i)B_\zeta(\boldsymbol{p}_i)|^k\}$, where $\mathrm{E}\left|B_\xi(\boldsymbol{p}_i) B_\zeta(\boldsymbol{p}_i)\right|^k \sim |\triangle|^3$, $\left|\mathrm{E}B_\xi(\boldsymbol{p}_i) B_\zeta(\boldsymbol{p}_i)\right|^k \sim |\triangle|^{3k}$. Then there exists a constant $C > 0$ such that $\mathrm{E}\left|R_{\xi,\zeta,i}\right|^k \le C2^{k-1}k!\mathrm{E}R_{\xi,\zeta,i}^2$. Thus, $\{R_{\xi,\zeta,i}\}_{i=1}^n$ satisfying Cramer's condition with some constant $C \sim O(1)$. By Bernstein's inequality in Bosq (1998), for $\delta > 0$ large enough,

$$P\left(\frac{1}{n}\left|\sum_{i=1}^n R_{\xi,\zeta,i}\right| \ge \delta\sqrt{\frac{\log n}{nN}}\right) \le 2\exp\left(\frac{-\delta^2 \log n}{4 + 2c\delta\sqrt{N\log n/n}}\right) \le 2n^{-4}. \tag{B.3}$$

It is easy to see that the cardinality of $\mathcal{M}$ is $(d+1)(d+2)(d+3)N/6$ as discussed in Section 2.3. Thus, for the $\delta > 0$ in Equation B.3,

$$\sum_{n=1}^\infty P\left(\max_{\xi,\zeta\in\mathcal{M}}\left|\frac{1}{n}\sum_{i=1}^n R_{\xi,\zeta,i}\right| \ge \delta\sqrt{\frac{\log n}{nN}}\right) \le \frac{1}{6}\sum_{n=1}^\infty \frac{\{(d+1)(d+2)(d+3)N\}^2}{n^4} \le C\sum_{n=1}^\infty n^{-2} < \infty.$$

Borel-Cantelli Lemma entails that $\max_{\xi,\zeta\in\mathcal{M}} |n^{-1}\sum_{l=1}^n R_{\xi,\zeta,i}| = O_P\{(\log n)^{1/2}(nN)^{-1/2}\}$. The desired result follows from Equations B.1 and B.2. ■

As a direct result of Lemma 3, we have

$$\sup_{g\in\mathcal{S}_d^r(\triangle)} \left|\|g\|_{n,\Omega}^2 \big/ \|g\|_\Omega^2 - 1\right| = O_P\left\{(N\log n)^{1/2} n^{-1/2}\right\}. \tag{B.4}$$

B.2 Size of the Bias and Noise Terms (Propositions 5—7)

In this section, we measure the size of the bias term and show the proofs of Proposition 5—7. We firstly proof Lemma 4.

Proof of Lemma 4. According to Lemma 15.2 of Lai and Schumaker (2007), for any $g \in \mathcal{S}_d^r(\triangle)$,

$$\|g\|_\infty = \|g\|_{\infty,T} \le K_1 V_T^{-1/2} \|g\|_{L^2(T)} \le \frac{K}{\varrho_\triangle^{3/2}} \|g\|_{L^2(T)} \le \frac{K}{\varrho_\triangle^{3/2}} \|g\|_{L^2(\Omega)} \le \frac{K_\beta}{|\triangle|^{3/2}} \|g\|_{L^2(\Omega)},$$

where K_β is a positive constant dependent on β and recall that V_T is the volume of tetrahedron T. Consequently, by the Markov's inequality Theorem 15.28 in Lai and Schumaker (2007), for any $g \in \mathcal{S}_d^r(\triangle)$, $\|g\|_{\infty,\Omega} \le K_\beta|\triangle|^{-3/2}\|g\|_{L^2(\Omega)}$ and $\|g\|_{\mathcal{E}} \le K_\beta|\triangle|^{-2}\|g\|_{L^2(\Omega)}$. Equation B.4 implies that

$$\sup_{g\in\mathcal{S}_d^r(\triangle)} \left\{ \|g\|_{n,\Omega} \big/ \|g\|_{L^2(\Omega)} \right\} \ge \left[1 - O_P\left\{(N\log n)^{1/2} n^{-1/2}\right\}\right]^{1/2}.$$

Thus, we have

$$\begin{aligned} V_n &\le K_\beta |\triangle|^{-3/2} \left[1 - O_P\left\{(N\log n)^{1/2} n^{-1/2}\right\}\right]^{-1/2} = O_P\left(|\triangle|^{-3/2}\right), \\ \overline{V}_n &\le K_\beta |\triangle|^{-2} \left[1 - O_P\left\{(N\log n)^{1/2} n^{-1/2}\right\}\right]^{-1/2} = O_P\left(|\triangle|^{-2}\right). \end{aligned}$$

■

Proof of Proposition 5. By triangle inequality and the definition of V_n,

$$\begin{aligned} \|m - s_{\rho_n,m}\|_{\infty,\Omega} &\le \|m - s_{0,m}\|_{\infty,\Omega} + \|s_{0,m} - s_{\rho_n,m}\|_{\infty,\Omega} \\ &\le \|m - s_{0,m}\|_{\infty,\Omega} + V_n \|s_{0,m} - s_{\rho_n,m}\|_{n,\Omega}. \end{aligned} \quad (B.5)$$

By the definition of $s_{\rho_n,m}$, we have $\forall u \in \mathcal{S}_d^r(\triangle)$, $t \in \mathbb{R}_+$,

$$\begin{aligned} & n\|m - s_{\rho_n,m}\|_{n,\Omega}^2 + \rho_n \|s_{\rho_n,m}\|_{\mathcal{E}}^2 \le n\|m - s_{\rho_n,m} - tu\|_{n,\Omega}^2 + \rho_n\|s_{\rho_n,m} + tu\|_{\mathcal{E}}^2 \\ \Rightarrow\quad & n\langle m - s_{\rho_n,m}, u\rangle_{n,\Omega} - \rho_n \langle s_{\rho_n,m}, u\rangle_{\mathcal{E}} \le \frac{t}{2}\left(\|u\|_{n,\Omega}^2 + \rho_n\|u\|_{\mathcal{E}}^2\right). \end{aligned}$$

Similarly, take $t \in \mathbb{R}_-$ and induct in a similar fashion, we thus obtain

$$\left| n\langle m - s_{\rho_n,m}, u\rangle_{n,\Omega} - \rho_n \langle s_{\rho_n,m}, u\rangle_{\mathcal{E}}\right| \le \frac{t}{2}\left(\|u\|_{n,\Omega}^2 + \rho_n\|u\|_{\mathcal{E}}^2\right). \quad (B.6)$$

Since Equation B.6 holds for any $t \in \mathbb{R}$, we can conclude that the penalized spline $s_{\rho_n,m}$ of m is characterized by the orthogonality relations

$$n\langle m - s_{\rho_n,m}, u\rangle_{n,\Omega} = \rho_n \langle s_{\rho_n,m}, u\rangle_{\mathcal{E}}, \quad \text{for all } u \in \mathcal{S}_d^r(\triangle). \quad (B.7)$$

When $\rho_n = 0$, we have for $s_{0,m}$,

$$\langle m - s_{0,m}, u\rangle_{n,\Omega} = 0, \quad \text{for all } u \in \mathcal{S}_d^r(\triangle). \quad (B.8)$$

Combining Equations B.7 and B.8, we obtain $n\left\langle s_{0,m}-s_{\rho_n,m},u\right\rangle_{n,\Omega}=\rho_n\left\langle s_{\rho_n,m},u\right\rangle_{\mathcal{E}}$, for all $u\in\mathcal{S}_d^r(\triangle)$. Inserting $u=s_{0,m}-s_{\rho_n,m}$ yields that

$$n\left\|s_{0,m}-s_{\rho_n,m}\right\|_{n,\Omega}^2=\rho_n\left\langle s_{\rho_n,m},s_{0,m}-s_{\rho_n,m}\right\rangle_{\mathcal{E}}. \tag{B.9}$$

Thus, by Cauchy-Schwarz inequality and the definition of $\overline{V}_n$,

$$n\left\|s_{0,m}-s_{\rho_n,m}\right\|_{n,\Omega}^2\le\rho_n\left\|s_{\rho_n,m}\right\|_{\mathcal{E}}\left\|s_{0,m}-s_{\rho_n,m}\right\|_{\mathcal{E}}\le\overline{V}_n\rho_n\left\|s_{\rho_n,m}\right\|_{\mathcal{E}}\left\|s_{0,m}-s_{\rho_n,m}\right\|_{n,\Omega}.$$

Similarly, using Equation B.9, we have

$$n\left\|s_{0,m}-s_{\rho_n,m}\right\|_{n,\Omega}^2=\rho_n\left\{\left\langle s_{\rho_n,m},s_{0,m}\right\rangle_{\mathcal{E}}-\left\langle s_{\rho_n,m},s_{\rho_n,m}\right\rangle_{\mathcal{E}}\right\}\ge 0.$$

Thus, by Cauchy-Schwarz inequality, $\|s_{\rho_n,m}\|_{\mathcal{E}}^2\le\langle s_{\rho_n,g},s_{0,m}\rangle_{\mathcal{E}}\le\|s_{\rho_n,m}\|_{\mathcal{E}}\|s_{0,m}\|_{\mathcal{E}}$, which implies that $\left\|s_{\rho_n,m}\right\|_{\mathcal{E}}\le\left\|s_{0,m}\right\|_{\mathcal{E}}$. Therefore

$$\left\|s_{0,m}-s_{\rho_n,m}\right\|_{n,\Omega}\le n^{-1}\overline{V}_n\rho_n\left\|s_{0,m}\right\|_{\mathcal{E}}. \tag{B.10}$$

Combining Equations B.5, B.10 and Lemma 1 in Section 2.3 in main part yields that

$$\begin{aligned}\left\|s_{0,m}-s_{\rho_n,m}\right\|_{\infty,\Omega} &\le V_n\left\|s_{0,m}-s_{\rho_n,m}\right\|_{n,\Omega}\le n^{-1}V_n\overline{V}_n\rho_n\left\|s_{0,m}\right\|_{\mathcal{E}}\\ &\le n^{-1}V_n\overline{V}_n\rho_nC_1\left(|m|_{2,\infty,\Omega}+\sum_{|\boldsymbol{\alpha}|=2}\left\|D^{\boldsymbol{\alpha}}\left(m-s_{0,m}\right)\right\|_{\infty,\Omega}\right)\\ &\le n^{-1}V_n\overline{V}_n\rho_nC_2\left(|m|_{2,\infty,\Omega}+|\triangle|^{\ell-1}|m|_{\ell+1,\infty,\Omega}\right).\end{aligned}$$

Plugging the orders of V_n and $\overline{V}_n$, therefore,

$$\left\|s_{0,m}-s_{\rho_n,m}\right\|_{\infty,\Omega}=O_P\left\{\frac{\rho_n}{n\,|\triangle|^{7/2}}\left(|m|_{2,\infty,\Omega}+|\triangle|^{\ell-1}|m|_{\ell+1,\infty,\Omega}\right)\right\}.$$

Hence by Equation B.5,

$$\left\|m-s_{\rho_n,m}\right\|_{\infty,\Omega}=O_P\left(|\triangle|^{\ell+1}|m|_{\ell+1,\infty,\Omega}\right)+O_P\left\{\frac{\rho_n}{n\,|\triangle|^{7/2}}\left(|m|_{2,\infty,\Omega}+|\triangle|^{\ell-1}|m|_{\ell+1,\infty,\Omega}\right)\right\}.$$

Therefore, Proposition 5 is established. ■

Next we give the proof of Proposition 6.

Proof of Proposition 6. It is known from Lai and Schumaker (2007) that there is a locally support basis B_ξ, $\xi\in\mathcal{M}$ for $\mathcal{S}_d^r(\triangle)$. We write $s_{\rho_n,\epsilon}(\boldsymbol{p})=\sum_{\xi\in\mathcal{M}}c_{\rho_n,\xi}B_\xi(\boldsymbol{p})$ for some coefficients $c_{\rho_n,\xi}$. It is easy to obtain the orthogonal relations for the penalized spline $s_{\rho_n,\epsilon}$ $n\langle s_{\rho_n,\epsilon}-\varepsilon,u\rangle_{n,\Omega}+\rho_n\langle s_{\rho_n,\epsilon},u\rangle_{\mathcal{E}}=0$, for all $u\in\mathcal{S}_d^r(\triangle)$. Consequently

$$\sum_{i=1}^n s_{\rho_n,\epsilon}\left(\boldsymbol{p}_i\right)B_\xi\left(\boldsymbol{p}_i\right)+\rho_n\langle s_{\rho_n,\epsilon},B_\xi\rangle_{\mathcal{E}}=\sum_{i=1}^n B_\xi\left(\boldsymbol{p}_i\right)\sigma\left(\boldsymbol{p}_i\right)\varepsilon_i$$

for all $\xi \in \mathcal{M}$. Multiply $c_{\rho_n,\xi}$ to both sides and take the summation of $\xi \in \mathcal{M}$ and use Cauchy-Schwarz's inequality,

$$\begin{aligned}\|s_{\rho_n,\epsilon}\|^2_{n,\Omega} &\leq \|s_{\rho_n,\epsilon}\|^2_{n,\Omega} + \frac{\rho_n}{n}\mathcal{E}(s_{\rho_n,\epsilon}) = \frac{1}{n}\sum_{\xi\in\mathcal{M}} c_{\rho_n,\xi}\sum_{i=1}^{n} B_\xi\left(\boldsymbol{p}_i\right)\sigma\left(\boldsymbol{p}_i\right)\varepsilon_i \\ &\leq \left(\sum_{\xi\in\mathcal{M}}|c_{\rho_n,\xi}|^2\right)^{1/2}\left[\sum_{\xi\in\mathcal{M}}\left\{\frac{1}{n}\sum_{i=1}^{n} B_\xi\left(\boldsymbol{p}_i\right)\sigma\left(\boldsymbol{p}_i\right)\varepsilon_i\right\}^2\right]^{1/2} \\ &\leq \frac{1}{K_1|\triangle|^{3/2}}\|s_{\rho_n,\epsilon}\|_{L^2(\Omega)}\left[\sum_{\xi\in\mathcal{M}}\left\{\frac{1}{n}\sum_{i=1}^{n} B_\xi\left(\boldsymbol{p}_i\right)\sigma\left(\boldsymbol{p}_i\right)\varepsilon_i\right\}^2\right]^{1/2}.\end{aligned}$$

It follows from Equation B.4 that

$$\begin{aligned}&\|s_{\rho_n,\epsilon}\|^2_{L^2(\Omega)} \leq \|s_{\rho_n,\epsilon}\|^2_{n,\Omega} + O_P\left\{n^{-1/2}(\log n)^{1/2}N^{1/2}\|s_{\rho_n,\epsilon}\|^2_{L^2(\Omega)}\right\} \\ &\leq \frac{\|s_{\rho_n,\epsilon}\|_{L^2(\Omega)}}{K_1|\triangle|^{3/2}}\left[\sum_{\xi\in\mathcal{M}}\left\{\frac{1}{n}\sum_{i=1}^{n} B_\xi\left(\boldsymbol{p}_i\right)\sigma\left(\boldsymbol{p}_i\right)\varepsilon_i\right\}^2\right]^{1/2} + O_P\left\{\frac{(N\log n)^{1/2}}{n^{1/2}}\|s_{\rho_n,\epsilon}\|^2_{L^2(\Omega)}\right\}.\end{aligned}$$

Next we note that $V_\Omega \leq C_1|\triangle|^3 N$. That is, $|\triangle| \geq (C_1 N)^{-1/3}V_\Omega^{1/3}$. We thus have

$$\begin{aligned}\|s_{\rho_n,\epsilon}\|_{L^2(\Omega)} \leq\ & \frac{(C_1N)^{1/2}}{V_\Omega^{1/2}K_1}\left[\sum_{\xi\in\mathcal{M}}\left\{\frac{1}{n}\sum_{i=1}^{n} B_\xi\left(\boldsymbol{p}_i\right)\sigma\left(\boldsymbol{p}_i\right)\varepsilon_i\right\}^2\right]^{1/2} \\ &+O_P\left\{n^{-1/2}(N\log n)^{1/2}\|s_{\rho_n,\epsilon}\|_{L^2(\Omega)}\right\}\end{aligned}$$

Thus, we have

$$\begin{aligned}&\left[1 - O_P\left\{\frac{(N\log n)^{1/2}}{n^{1/2}}\right\}\right]\|s_{\rho_n,\epsilon}\|_{L^2(\Omega)} \\ &\qquad\leq \frac{CN^{1/2}}{V_\Omega^{1/2}}\left[\sum_{\xi\in\mathcal{M}}\left\{\frac{1}{n}\sum_{i=1}^{n} B_\xi\left(\boldsymbol{p}_i\right)\sigma\left(\boldsymbol{p}_i\right)\varepsilon_i\right\}^2\right]^{1/2} \qquad \text{(B.11)}\end{aligned}$$

for a constant $C > 0$. Observing that the two random variables ε_i and $\boldsymbol{p}_i$ are independent, we have $\mathrm{E}\{n^{-1}\sum_{i=1}^n \varepsilon_i B_\xi(\boldsymbol{p}_i)\sigma(\boldsymbol{p}_i)\}^2 = n^{-1}\mathrm{E}[B_\xi^2(\boldsymbol{p}_i)\sigma^2(\boldsymbol{p}_i)]$, where according to Lemma 15.2 in Lai and Schumaker (2007), the expectation of $B_\xi^2\left(\boldsymbol{p}_i\right)\sigma^2\left(\boldsymbol{p}_i\right)$ can be estimated as follows:

$$\begin{aligned}\mathrm{E}\left[B_\xi^2\left(\boldsymbol{p}_i\right)\sigma^2\left(\boldsymbol{p}_i\right)\right] &\leq C_\sigma^2\left\|B_\xi\right\|^2_{L^2(\Omega)} \\ &\leq c(\beta,\sigma)|\triangle|^3\|B_\xi\|^2_{\infty,\Omega} \leq C(\beta,\sigma)N^{-1}V_\Omega \qquad \text{(B.12)}\end{aligned}$$

for positive constants $c(\beta,\sigma)$ and $C(\beta,\sigma)$ which depend only on β and σ. Thus, Assumption (A2) and Equation B.12 imply that $n^{-1}\sum_{i=1}^n B_\xi(\boldsymbol{p}_i)\sigma(\boldsymbol{p}_i)\varepsilon_i = O_P(n^{-1/2}N^{-1/2})$. Therefore,

we have $\sum_{\xi \in \mathcal{M}}\{n^{-1}\sum_{i=1}^{n} B_{\xi}(\boldsymbol{p}_i)\sigma(\boldsymbol{p}_i)\varepsilon_i\}^2 = O_P(n^{-1})$. Combining Equation B.11, we obtain that $\|s_{\rho_n,\epsilon}\|_{L^2(\Omega)} = O_P\left(N^{1/2}n^{-1/2}\right)$. So Proposition 6 is established. ■

Let $\boldsymbol{\Gamma}_{\rho_n}$ be the symmetric positive definite matrix

$$\left[\frac{1}{n}\sum_{i=1}^{n} B_{\xi}\left(\boldsymbol{p}_i\right) B_{\zeta}\left(\boldsymbol{p}_i\right) + \frac{\rho_n}{n}\langle B_{\xi}, B_{\zeta}\rangle_{\mathcal{E}}\right]_{\xi,\zeta\in\mathcal{M}}. \tag{B.13}$$

We firstly show the bounds of $\boldsymbol{\Gamma}_{\rho_n}$.

Lemma B.3 *Suppose Assumption (A3) holds, $d \geq 6r+3$, $\triangle$ is a β-quasi-uniform triangulation and $n^{-1}N\log n \to 0$ as $n \to \infty$. For $\boldsymbol{\Gamma}_{\rho_n}$ defined in Equation B.13, we have the following asymptotic properties:*

(i) As $n \to \infty$, for some constants $0 < c_\rho < C_\rho < \infty$, with probability approaching one,

$$c_\rho|\triangle|^3 \leq \rho_{\min}(\boldsymbol{\Gamma}_{\rho_n}) \leq \rho_{\max}(\boldsymbol{\Gamma}_{\rho_n}) \leq C_\rho\left(|\triangle|^3 + \frac{\rho_n}{n|\triangle|}\right).$$

(ii) There exists a constant $M_d > 0$ such that $\|\boldsymbol{\Gamma}_{\rho_n}^{-1}\|_\infty \leq M_d|\triangle|^{-3}$.

(iii) For every vector $\boldsymbol{a} = (a_1,\ldots,a_n)^\top$, there exists a constant $C_d > 0$ such that

$$\left\|\mathbf{B}^\top(\boldsymbol{p})\ \boldsymbol{\Gamma}_{\rho_n}^{-1}\frac{1}{n}\sum_{i=1}^{n}\mathbf{B}(\boldsymbol{p}_i)a_i\right\|_\infty \leq C_d\|\boldsymbol{a}\|_\infty.$$

Proof (i) Let's randomly pick $\boldsymbol{\theta} \in \mathbb{R}^{|\mathcal{M}|}$, then for $g(\boldsymbol{p}) = \mathbf{B}^\top(\boldsymbol{p})\boldsymbol{\theta}$, we have $g \in \mathcal{S}_d^r(\triangle)$. Then

$$\boldsymbol{\theta}^\top\boldsymbol{\Gamma}_{\rho_n}\boldsymbol{\theta} = \boldsymbol{\theta}^\top\frac{1}{n}\sum_{i=1}^{n}\mathbf{B}(\boldsymbol{p}_i)\mathbf{B}^\top(\boldsymbol{p}_i)\boldsymbol{\theta} + \boldsymbol{\theta}^\top\frac{\rho_n}{n}[\langle B_m, B_{m'}\rangle_{\mathcal{E}}]_{m,m'\in\mathcal{M}}\boldsymbol{\theta} = \|g\|_{n,\Omega}^2 + \frac{\rho_n}{n}\|g\|_{\mathcal{E}}^2.$$

By Equation B.4, $\left|\|g\|_{n,\Omega}^2/\|g\|_{L^2(\Omega)}^2 - 1\right| \leq R_n$, and combined with Lemma B.1, we have

$$c(1-R_n)|\triangle|^3\|\boldsymbol{\theta}\|^2 \leq (1-R_n)\|g\|_{L^2(\Omega)}^2 \leq \|g\|_{n,\Omega}^2 \leq (1+R_n)\|g\|_{L^2(\Omega)}^2 \leq C(1+R_n)|\triangle|^3\|\boldsymbol{\theta}\|^2.$$

Thus, $\rho_{\min}(\boldsymbol{\Gamma}_{\rho_n}) \geq c_\rho|\triangle|^3$ for some positive constant c_ρ.

On the other side, as shown in the proof of Proposition 5 and by Lemma B.1, $\|g\|_{\mathcal{E}}^2 \leq C|\triangle|^{-4}\|g\|_{L^2(\Omega)}^2 \leq C|\triangle|^{-1}\|\boldsymbol{\theta}\|^2$. Thus,

$$\rho_{\max}(\boldsymbol{\Gamma}_{\rho_n}) \leq C\left\{(1+R_n)|\triangle|^3 + \frac{\rho_n}{n}\frac{1}{|\triangle|}\right\} \leq C_\rho\left(|\triangle|^3 + \frac{\rho_n}{n|\triangle|}\right),$$

for some positive constant C_ρ.

(ii) By (i), $\boldsymbol{\Gamma}_{\rho_n}$ is an invertible symmetric matrix, and its condition number $c_d = \rho_{\max}(\boldsymbol{\Gamma}_{\rho_n})/\rho_{\min}(\boldsymbol{\Gamma}_{\rho_n})$ satisfies $1 < c_d \leq c_\rho^{-1}C_\rho$.

According to the definition of banded matrix in DeVore and Lorentz (1993), a matrix $\mathbf{A} = (a_{ij})$ is said banded with bandwidth b if $a_{ij} = 0, |i-j| \geq b$, and if b is the smallest

integer with this property. Based on the construction of trivariate splines, $\mathbf{\Gamma}_{\rho_n}$ is a banded matrix with bandwidth $b = \binom{d+3}{3}$. By Theorem 13.4.3 in DeVore and Lorentz (1993), $\|\mathbf{\Gamma}_{\rho_n}^{-1}\|_\infty \le 2\tau^{-2b}\|\mathbf{\Gamma}_{\rho_n}^{-1}\|_2(1-\tau)^{-1}$, where $\tau = (c_d^2 - 1/c_d^2 + 1)^{1/4b} < 1$. Therefore, there exist some positive constant M_d such that $\|\mathbf{\Gamma}_{\rho_n}^{-1}\|_\infty \le M_d|\triangle|^{-3}$.

(iii) Combining (i) and (ii), it is straightforward to have

$$\left\|\mathbf{B}^\top(\boldsymbol{p})\mathbf{\Gamma}_{\rho_n}^{-1}\frac{1}{n}\sum_{i=1}^{n}\mathbf{B}(\boldsymbol{p}_j)a_j\right\|_\infty \le \left\|\mathbf{B}^\top\right\|_\infty \|\mathbf{\Gamma}_{\rho_n}^{-1}\|_\infty \left\|\frac{1}{n}\sum_{i=1}^{n}\mathbf{B}(\boldsymbol{p}_i)\right\|_\infty \|\boldsymbol{a}\|_\infty \le C_d\|\boldsymbol{a}\|_\infty.$$

■

Lemma B.4 *Under Assumptions (A2) and (A4),* $\|s_{0,\epsilon}\|_{\infty,\Omega} = O_P\{(\log n)^{1/2}n^{-1/2}|\triangle|^{-3/2}\}$.

Proof Note that $s_{0,\epsilon}(\boldsymbol{p}) = \sum_{\xi\in\mathcal{M}}\widehat{c}_{0,\xi}B_\xi(\boldsymbol{p})$ for some coefficients $\widehat{c}_{0,\xi}$, so the order of $s_{\rho_n,\epsilon}(\boldsymbol{p})$ is related to that of $\widehat{c}_{0,\xi}$. In fact

$$\|s_{0,\epsilon}\|_{\infty,\Omega} \le c\,\|\widehat{\mathbf{c}}_0\|_\infty = \left\|\mathbf{\Gamma}_0^{-1}\left[n^{-1}\sum_{i=1}^{n}B_\xi\left(\boldsymbol{p}_i\right)\sigma\left(\boldsymbol{p}_i\right)\varepsilon_i\right]_{\xi\in\mathcal{M}}\right\|_{\infty,\Omega},$$

where $\widehat{\mathbf{c}}_0 = (\widehat{c}_{0,\xi})_{\xi\in\mathcal{M}}$ and $\mathbf{\Gamma}_0$ is the symmetric positive definite matrix

$$\left[\frac{1}{n}\sum_{i=1}^{n}B_\xi\left(\boldsymbol{p}_i\right)B_\zeta\left(\boldsymbol{p}_i\right)\right]_{\xi,\zeta\in\mathcal{M}}$$

defined in Equation B.13. Thus,

$$\|s_{0,\epsilon}\|_{\infty,\Omega} \le CN\max_{\xi\in\mathcal{M}}\left|n^{-1}\sum_{i=1}^{n}B_\xi\left(\boldsymbol{p}_i\right)\sigma\left(\boldsymbol{p}_i\right)\varepsilon_i\right|, \quad a.s.$$

Next, we show that with probability one,

$$\max_{\xi\in\mathcal{M}}\left|n^{-1}\sum_{i=1}^{n}B_\xi\left(\boldsymbol{p}_i\right)\sigma\left(\boldsymbol{p}_i\right)\varepsilon_i\right| = O\left\{(\log n)^{1/2}(nN)^{-1/2}\right\}. \tag{B.14}$$

To prove Equation B.14, we decompose the noise variable ε_i into a truncated part and a tail part $\varepsilon_i = \varepsilon_{i,1}^{D_n} + \varepsilon_{i,2}^{D_n} + \mu^{D_n}$, where $D_n = n^\alpha$ with $\max\{1/(2+\eta),(1+\gamma)/2(1+\eta)\} < \alpha < (1-\gamma)/2$, and $\varepsilon_{i,1}^{D_n} = \varepsilon_i I\left\{|\varepsilon_i| > D_n\right\}$,

$$\varepsilon_{i,2}^{D_n} = \varepsilon_i I\left\{|\varepsilon_i| \le D_n\right\} - \mu^{D_n}, \quad \mu^{D_n} = \mathrm{E}\left[\varepsilon_i I\left\{|\varepsilon_i| \le D_n\right\}\right].$$

It is straightforward to see $\varepsilon_{i,2}^{D_n}$ has mean 0, thus is uniformly bounded by D_n^{-2}. And it is also straightforward to verify that μ^{D_n} is uniformly bounded by $D_n^{-(1+\eta)}$, so the boundedness of trivariate spline basis and of the function σ^2 entail that

$$\left|B_\xi\left(\boldsymbol{p}_i\right)\sigma\left(\boldsymbol{p}_i\right)\mu^{D_n}\right| = O\left(D_n^{-(1+\eta)}\right). \tag{B.15}$$

Next we show that tail part vanishes almost surely. Recall that $\mathrm{E}|\varepsilon_n^{2+\eta}| \le \upsilon_\eta$ in Assumption (A2), so

$$\sum_{n=1}^{\infty} P\left\{|\varepsilon_n| > D_n\right\} \le \sum_{n=1}^{\infty} \frac{E\left|\varepsilon_n\right|^{2+\eta}}{D_n^{2+\eta}} \le \upsilon_\eta \sum_{n=1}^{\infty} D_n^{-(2+\eta)} < \infty.$$

By the Borel-Cantelli Lemma, we have

$$P\left\{\omega | \exists N(\omega), |\varepsilon_n(\omega)| \le D_n \text{ for } n > N(\omega)\right\} = 1.$$

Let $\upsilon_\varepsilon = \max\{|\varepsilon_1|, |\varepsilon_2|, ..., |\varepsilon_{N(\omega)}|\}$ and there exists $N_1(\omega) > N(\omega)$, $D_{N_1(\omega)} > \upsilon_\varepsilon$. Since $D_n = n^\alpha$ is an increasing function, we have $D_n > D_{N_1(\omega)} > \upsilon_\varepsilon$, for $n > N_1(\omega)$. Thus,

$$P\left\{\omega | \exists N(\omega), |\varepsilon_i(\omega)| \le D_n,\ 1 \le i \le n, \text{ for } n > N(\omega)\right\} = 1,$$

which implies that

$$P\left\{\omega | \exists N(\omega), \left|\varepsilon_{i,1}^{D_n}\right| = 0,\ 1 \le i \le n, \text{ for } n > N(\omega)\right\} = 1.$$

The boundedness of the spline basis implies that

$$\left|\frac{1}{n}\sum_{i=1}^{n} \sigma(\boldsymbol{p}_i)\, \varepsilon_{i,1}^{D_n} B_\xi(\boldsymbol{p}_i)\right| = O_{a.s.}\left(n^{-k}\right), \text{ for any } k > 0. \tag{B.16}$$

Next let $Z_i = n^{-1}\varepsilon_{i,2}^{D_n}\sigma(\boldsymbol{p}_i) B_\xi(\boldsymbol{p}_i)$. Since $\mathrm{E}(\varepsilon_{i,2}^{D_n}) = 0$, we have

$$\mathrm{Var}\left(\varepsilon_{i,2}^{D_n}\right) = \mathrm{E}\left(\varepsilon_i^2\right) - \mathrm{E}\left[\varepsilon_i^2 I\left\{|\varepsilon_i| > D_n\right\}\right] - \left(\mu^{D_n}\right)^2 = 1 + O_P\left\{D_n^{-\eta} + D_n^{-2(1+\eta)}\right\}.$$

According to Equation B.12, $\mathrm{E}[B_\xi^2(\boldsymbol{p}_i)\,\sigma^2(\boldsymbol{p}_i)] \le C(\beta,\sigma)N^{-1}V_\Omega$. Note the independence of $\varepsilon_{i,2}^{D_n}, i = 1, \cdots, n$ and the independence between $\varepsilon_{i,2}^{D_n}$ and $B_\xi(\boldsymbol{p}_i)$, we therefore obtain that $V_n^2 = \mathrm{Var}\left(\sum_{i=1}^n Z_i\right) = c\,(nN)^{-1}$ for some $c > 0$. By the fact that $|\varepsilon_{i,2}^{D_n}| < 2D_n$, we have

$$\mathrm{E}\left|\varepsilon_{i,2}^{D_n}\right|^k \le 2^{k-2}D_n^{k-2}\mathrm{E}\left|\varepsilon_{i,2}^{D_n}\right|^2, \quad k \ge 2.$$

Note that

$$\begin{aligned}\mathrm{E}\left|Z_i\right|^k &= n^{-k}\mathrm{E}\left|\varepsilon_{i,2}^{D_n}\right|^k \mathrm{E}\left|\sigma(\boldsymbol{p}_i) B_\xi(\boldsymbol{p}_i)\right|^k \\ &\le n^{-k}\mathrm{E}\left|\varepsilon_{i,2}^{D_n}\right|^k C_\sigma^{k-2}\left\|B_\xi\right\|_{\infty,\Omega}^{k-2}\mathrm{E}\left|\sigma(\boldsymbol{p}_i) B_\xi(\boldsymbol{p}_i)\right|^2 \le (2D_n n^{-1})^{k-2}k!\mathrm{E}(Z_i^2).\end{aligned}$$

Thus, $\{Z_i\}_{i=1}^n$ satisfies the Cramer condition with constant $c^* = 2n^{-1}D_n$. By the Bernstein inequality, for any $\delta > 0$ large enough,

$$P\left(\left|\sum_{i=1}^n Z_i\right| \ge \delta\sqrt{\frac{\log n}{nN}}\right) \le 2\exp\left(\frac{-\delta^2\frac{\log n}{nN}}{4V_n^2 + 2c^*\delta\sqrt{\frac{\log n}{nN}}}\right) = 2\mathrm{exp}\left(\frac{-\delta^2\log n}{4c + 4D_n\delta\sqrt{\frac{N\log n}{n}}}\right) \le 2n^{-3}.$$

Therefore,

$$\sum_{n=1}^{\infty} P\left(\max_{\xi\in\mathcal{M}}\left|\frac{1}{n}\sum_{i=1}^{n}\varepsilon_{i,2}^{D_n}\sigma\left(\boldsymbol{p}_i\right)B_{\xi}\left(\boldsymbol{p}_i\right)\right|\geq\delta\sqrt{\frac{\log n}{nN}}\right)\leq\frac{N}{3}(d+1)(d+2)(d+3)\sum_{n=1}^{\infty}n^{-3}<\infty$$

for such $\delta > 0$. Thus, the conclusion follows by combining Borel-Cantelli's lemma with Equations B.15 and B.16. ■

Proof of Proposition 7. Note that the penalized spline $s_{\rho_n,\epsilon}$ of ε is characterized by the orthogonality relations

$$n\left\langle\varepsilon-s_{\rho_n,\varepsilon},u\right\rangle_{n,\Omega}=\rho_n\left\langle s_{\rho_n,\varepsilon},u\right\rangle_{\mathcal{E}},\quad\text{for all } u\in\mathcal{S}_d^r(\triangle). \tag{B.17}$$

In particular, $s_{0,\varepsilon}$ is characterized by

$$\left\langle\varepsilon-s_{0,\varepsilon},u\right\rangle_{n,\Omega}=0,\quad\text{for all } u\in\mathcal{S}_d^r(\triangle). \tag{B.18}$$

Inserting $u = s_{0,\varepsilon} - s_{\rho_n,\epsilon}$ in Equation B.17 and using Equation B.18 with this u yield that

$$\begin{aligned} n\left\|s_{0,\varepsilon}-s_{\rho_n,\varepsilon}\right\|_{n,\Omega}^2 &= \rho_n\left\langle s_{\rho_n,\varepsilon},s_{0,\varepsilon}-s_{\rho_n,\varepsilon}\right\rangle_{\mathcal{E}} \\ &= \rho_n(\langle s_{\rho_n,\epsilon},s_{0,\epsilon}\rangle_{\mathcal{E}}-\langle s_{\rho_n,\epsilon},s_{\rho_n,\epsilon}\rangle_{\mathcal{E}}). \end{aligned}$$

It follows, by Cauchy-Schwarz inequality, that

$$\left\|s_{\rho_n,\varepsilon}\right\|_{\mathcal{E}}^2\leq\left\langle s_{\rho_n,\varepsilon},s_{0,\varepsilon}\right\rangle_{\mathcal{E}}\leq\left\|s_{\rho_n,\varepsilon}\right\|_{\mathcal{E}}\left\|s_{0,\varepsilon}\right\|_{\mathcal{E}},$$

which implies that $\|s_{\rho_n,\varepsilon}\|_{\mathcal{E}} \leq \|s_{0,\varepsilon}\|_{\mathcal{E}}$. Thus, by Cauchy-Schwarz inequality and the definition of $\overline{V}_n$.

$$n\left\|s_{0,\varepsilon}-s_{\rho_n,\varepsilon}\right\|_{n,\Omega}^2\leq\rho_n\left\|s_{\rho_n,\varepsilon}\right\|_{\mathcal{E}}\left\|s_{0,\varepsilon}-s_{\rho_n,\varepsilon}\right\|_{\mathcal{E}}\leq\overline{V}_n\rho_n\left\|s_{\rho_n,\varepsilon}\right\|_{\mathcal{E}}\left\|s_{0,\varepsilon}-s_{\rho_n,\varepsilon}\right\|_{n,\Omega}.$$

Hence, we have

$$\left\|s_{0,\varepsilon}-s_{\rho_n,\varepsilon}\right\|_{n,\Omega}\leq n^{-1}\overline{V}_n\rho_n\left\|s_{0,\varepsilon}\right\|_{\mathcal{E}}. \tag{B.19}$$

Combining Equations 11 and B.19 yields that

$$\left\|s_{0,\varepsilon}-s_{\rho_n,\varepsilon}\right\|_{\infty,\Omega}\leq V_n\left\|s_{0,\varepsilon}-s_{\rho_n,\varepsilon}\right\|_{n,\Omega}\leq n^{-1}V_n\overline{V}_n\rho_n\left\|s_{0,\varepsilon}\right\|_{\mathcal{E}}.$$

Again, applying Markov's inequality Theorem 15.28 in Lai and Schumaker (2007), we get

$$\left\|s_{0,\varepsilon}\right\|_{\mathcal{E}}\leq\frac{C_1}{|\triangle|^2}\left\|s_{0,\varepsilon}\right\|_{L^2(\Omega)}.$$

It therefore follows

$$\|s_{\rho_n,\varepsilon}\|_{\infty,\Omega}\leq\|s_{0,\epsilon}\|_{\infty,\Omega}+\|s_{0,\varepsilon}-s_{\rho_n,\epsilon}\|_{\infty,\Omega}\leq\|s_{0,\varepsilon}\|_{\infty,\Omega}+\frac{\rho_n}{n}V_n\bar{V}_n\frac{C_1}{|\triangle|^2}\|s_{0,\varepsilon}\|_{L^2(\Omega)}.$$

According to Lemma B.4, we have $\|s_{0,\varepsilon}\|_{\infty,\Omega} = O_P\left\{n^{-1/2}|\triangle|^{-3/2}(\log n)^{1/2}\right\}$. The conclusion of Proposition 7 follows from Proposition 6. ■

B.3 Variance of the Noise Term

In this section, we first derive the size of the asymptotic conditional variance given in Theorem B.5.

Theorem B.5 *Under Assumptions (A1)—(A4), we have with probability approaching one as* $n \to \infty$

$$\frac{C_1 c_\sigma^2}{n(1+n^{-1}|\triangle|^{-4}\rho_n)^2|\triangle|^3} \leq \operatorname{Var}\{s_{\rho_n,\epsilon}(\boldsymbol{p})|\mathbb{P}\} \leq \frac{C_2 C_\sigma^2}{n|\triangle|^3},\ \boldsymbol{p} \in \Omega,$$

for positive constants C_1 *and* C_2.

Proof Note that

$$s_{\rho_n,\epsilon}(\boldsymbol{p}) = \mathbf{B}(\boldsymbol{p})^\top \widehat{\mathbf{c}}_{\rho_n,\varepsilon}, \tag{B.20}$$

where $\widehat{\mathbf{c}}_{\rho_n,\varepsilon}$ is the coefficient vector for $s_{\rho_n,\epsilon}$ using basis functions B_ξ, $\xi \in \mathcal{M}$ and $\mathbf{B}(\boldsymbol{p}) = [B_\xi(\boldsymbol{p}),\ \xi \in \mathcal{M}]^\top$ is the vector of basis functions. Note that

$$\widehat{\mathbf{c}}_{\rho_n,\varepsilon} = \left[\sum_{i=1}^n B_\xi(\boldsymbol{p}_i) B_\zeta(\boldsymbol{p}_i) + \rho_n \langle B_\xi, B_\zeta\rangle_{\mathcal{E}}\right]^{-1}_{\xi,\zeta\in\mathcal{M}} \left[\sum_{i=1}^n B_\xi(\boldsymbol{p}_i)\sigma(\boldsymbol{p}_i)\varepsilon_i\right]_{\xi\in\mathcal{M}}$$

and $\operatorname{Var}\{s_{\rho_n,\epsilon}(\boldsymbol{p})|\mathbb{P}\} = \mathbf{B}(\boldsymbol{p})^\top \mathrm{E}\left(\widehat{\mathbf{c}}_{\rho_n,\varepsilon}\widehat{\mathbf{c}}_{\rho_n,\varepsilon}^\top|\mathbb{P}\right)\mathbf{B}(\boldsymbol{p})$. Recall the definition of $\boldsymbol{\Gamma}_{\rho_n}$ in Equation B.13 in main part, we have $\mathrm{E}\left(\widehat{\mathbf{c}}_{\rho_n,\varepsilon}\widehat{\mathbf{c}}_{\rho_n,\varepsilon}^\top|\mathbb{P}\right)$ equal to

$$\boldsymbol{\Gamma}_{\rho_n}^{-1}\mathrm{E}\left(\left[\frac{1}{n}\sum_{i=1}^n B_\xi(\boldsymbol{p}_i)\sigma(\boldsymbol{p}_i)\varepsilon_i\right]_{\xi\in\mathcal{M}}\left[\frac{1}{n}\sum_{i=1}^n B_\xi(\boldsymbol{p}_i)\sigma(\boldsymbol{p}_i)\varepsilon_i\right]^\top_{\xi\in\mathcal{M}}\middle|\mathbb{P}\right)\boldsymbol{\Gamma}_{\rho_n}^{-1}.$$

The central conditional expectation term in the above line satisfies that

$$\frac{c_\sigma^2}{n}\boldsymbol{\Gamma}_0 \leq \frac{1}{n^2}\left[\sum_{i=1}^n B_\xi(\boldsymbol{p}_i)B_\zeta(\boldsymbol{p}_i)\sigma^2(\boldsymbol{p}_i)\right]_{\xi,\zeta\in\mathcal{M}} \leq \frac{C_\sigma^2}{n}\boldsymbol{\Gamma}_0.$$

That is,

$$n^{-1}c_\sigma^2\mathbf{B}(\boldsymbol{p})^\top\boldsymbol{\Gamma}_{\rho_n}^{-1}\boldsymbol{\Gamma}_0\boldsymbol{\Gamma}_{\rho_n}^{-1}\mathbf{B}(\boldsymbol{p}) \leq \operatorname{Var}\{s_{\rho_n,\epsilon}(\boldsymbol{p})|\mathbb{P}\} \leq n^{-1}C_\sigma^2\mathbf{B}(\boldsymbol{p})^\top\boldsymbol{\Gamma}_{\rho_n}^{-1}\boldsymbol{\Gamma}_0\boldsymbol{\Gamma}_{\rho_n}^{-1}\mathbf{B}(\boldsymbol{p}).$$

Let $\alpha_{\min}(\rho_n)$ and $\alpha_{\max}(\rho_n)$ be the smallest and largest eigenvalues of the positive definite matrix $\boldsymbol{\Gamma}_{\rho_n}$. It follows easily that with probability approaching one,

$$\frac{c_\sigma^2}{n}\alpha_{\max}(\rho_n)^{-2}\alpha_{\min}(0)\|\mathbf{B}(\boldsymbol{p})\|^2 \leq \operatorname{Var}\{s_{\rho_n,\epsilon}(\boldsymbol{p})|\mathbb{P}\} \leq \frac{C_\sigma^2}{n}\alpha_{\min}(\rho_n)^{-2}\alpha_{\max}(0)\|\mathbf{B}(\boldsymbol{p})\|^2.$$

Note that $\|\mathbf{B}(\boldsymbol{p})\|^2 = \sum_{\xi\in\mathcal{M}} B_\xi^2(\boldsymbol{p})$ is bounded above by a constant $C_2 < \infty$ and below by $C_1 > 0$ for any point $\boldsymbol{p} \in \Omega$. Indeed, if $C_1 = 0$ for a point $\boldsymbol{p}$, then $B_\xi(\boldsymbol{p}) = 0$ for all $\xi \in \mathcal{M}$. That is, we have $0 = \sum_{\xi\in\mathcal{M}} B_\xi(\boldsymbol{p})$. It follows that these basis functions are linearly dependent which is a contradiction. On the other hand, for any fixed point $\boldsymbol{p} \in \Omega$,

there is at most C_2 nonzero terms in the above summation, where C_2 is dependent on the smallest angle of the triangulation $\triangle$. Note that B_ξ is uniformly bounded for all $\xi \in \mathcal{M}$, say bounded by 1. Thus, we know $C_2 < \infty$. We summarize the above discussion to get

$$n^{-1}C_1c_\sigma^2\alpha_{\max}(\rho_n)^{-2}\alpha_{min}(0) \le \mathrm{Var}\left\{s_{\rho_n,\epsilon}(\boldsymbol{p})|\mathbb{P}\right\} \le n^{-1}C_2C_\sigma^2\alpha_{\min}(\rho_n)^{-2}\alpha_{\max}(0)$$

with probability approaching one.

We now spend some effort to estimate the largest and smallest eigenvalues of $\mathbf{\Gamma}_{\rho_n}$. It is easy to see that for any vector $\mathbf{a} = [a_\xi, \xi \in \mathcal{M}]^\top$,

$$\begin{aligned} \mathbf{a}^\top\mathbf{\Gamma}_{\rho_n}\mathbf{a} &= \mathbf{a}^\top \left[\frac{1}{n}\sum_{i=1}^n B_\xi\left(\boldsymbol{p}_i\right)B_\zeta\left(\boldsymbol{p}_i\right) + \frac{\rho_n}{n}\langle B_\xi, B_\zeta\rangle_{\mathcal{E}}\right]_{\xi,\zeta\in\mathcal{M}} \mathbf{a} \\ &= \left[\frac{1}{n}\sum_{i=1}^n\left\{\sum_{\xi\in\mathcal{M}} a_\xi B_\xi\left(\boldsymbol{p}_i\right)\right\}^2\right] + \frac{\rho_n}{n}\mathcal{E}\left(\sum_{\xi\in\mathcal{M}} a_\xi B_\xi\right). \end{aligned}$$

Let $s_a = \sum_{\xi\in\mathcal{M}} a_\xi B_\xi \in \mathcal{S}_d^r(\triangle)$ be the spline associated with vector $\mathbf{a} = (a_\xi, \xi \in \mathcal{M})^T$. By Equation B.4, we have

$$\|s_a\|_{n,\Omega}^2 = \left\{1 + O_P\left(\sqrt{\frac{\log n}{n/N}}\right)\right\}\|s_a\|_{L^2(\Omega)}^2 \le K_2\left\{1 + O_P\left(\sqrt{\frac{\log n}{n/N}}\right)\right\}|\triangle|^3\|\mathbf{a}\|^2.$$

Here we have used the stability conditions in Lemma B.1. Furthermore, using Markov's inequality, we have

$$\frac{\rho_n}{n}\mathcal{E}\left(\sum_{\xi\in\mathcal{M}} a_\xi B_\xi\right) \le \frac{\rho_n}{n}\frac{C}{|\triangle|^4}\|s_a\|_{L^2(\Omega)}^2 \le \frac{\rho_n}{n}\frac{C}{|\triangle|}K_2\|\mathbf{a}\|^2.$$

Thus, the largest eigenvalue $\alpha_{\max}(\rho_n)$ of the matrix $\mathbf{\Gamma}_{\rho_n}$ in Equation B.13 is less than or equal to

$$K_2\left\{1 + O_P\left(\sqrt{\frac{\log n}{n/N}}\right)\right\}|\triangle|^3 + K_2\frac{\rho_n}{n}\frac{C}{|\triangle|}.$$

Thus, we have with probability approaching one

$$\begin{cases} \alpha_{\max}(0) \le C|\triangle|^3, & \text{if } \rho_n = 0 \\ \alpha_{\max}(\rho_n) \le C\left(|\triangle|^3 + \frac{\rho_n}{n|\triangle|}\right), & \text{if } \rho_n > 0 \end{cases}$$

for positive constant C. On the other hand, we use Lemma B.1 and Equation B.4 to have

$$\|s_a\|_{n,\Omega}^2 = \left\{1 + O_P\left(\sqrt{\frac{\log n}{n/N}}\right)\right\}\|s_a\|_{L^2(\Omega)}^2 \ge K_1\left\{1 + O_P\left(\sqrt{\frac{\log n}{n/N}}\right)\right\}|\triangle|^3\|\mathbf{a}\|^2.$$

Therefore, the smallest eigenvalue $\alpha_{\min}(\rho_n)$ of the matrix $\mathbf{\Gamma}_{\rho_n}$ in Equation B.13 is greater than $K_1\{1 + O_P(\sqrt{Nn^{-1}\log n})\}|\triangle|^3 = C|\triangle|^3$. Summarizing the above discussions to conclude that for $\rho_n = 0$, we have with probability approaching one,

$$c_\beta c_\sigma^2 n^{-1}|\triangle|^{-3} \le \mathrm{Var}\left\{s_{\rho_n,\epsilon}(\boldsymbol{p})|\mathbb{P}\right\} \le C_\beta C_\sigma^2 n^{-1}|\triangle|^{-3}.$$

This establishes the result in the case for $\rho_n = 0$. Similar for the case $\rho_n > 0$. We have therefore completed the proof. ■

The above variance result can be more precise when spline space $\mathcal{S}_0^{-1}(\triangle)$ is considered. The next lemma provides the pointwise variance of $\widehat{s}_{\rho_n,\varepsilon}$ when using spline space $\mathcal{S}_0^{-1}(\triangle)$.

Lemma B.6 *Consider piecewise constant spline space $\mathcal{S}_0^{-1}(\triangle)$ and suppose that the tetrahedra are of equal size. Under Assumptions (A1)—(A4), if the density function $f(\boldsymbol{p})$ of $\boldsymbol{p}$ is continuous and positive on Ω, then*

$$\mathrm{Var}\left\{s_{\rho_n,\epsilon}\left(\boldsymbol{p}\right)\right\} = \frac{1}{n}\frac{\sigma^2\left(\boldsymbol{p}\right)}{f\left(\boldsymbol{p}\right)V_T}\{1+o(1)\},$$

where V_T is the volume of the tetrahedron T as defined before.

Proof When using spline space $\mathcal{S}_0^{-1}(\triangle)$, the space of piecewise constant functions over $\triangle$, we have $\mathcal{E}(s) = 0$ for all $s \in \mathcal{S}_0^{-1}(\triangle)$. Thus, following Equation B.20, we have $s_{\rho_n,\varepsilon}\left(\boldsymbol{p}\right) = \mathbf{B}(\boldsymbol{p})^\top \widehat{\mathbf{c}}_{\rho_n,\varepsilon}$, where

$$\widehat{\mathbf{c}}_{\rho_n,\varepsilon} = \left[\|B_\xi\|_{n,\Omega}^{-2}\frac{1}{n}\sum_{i=1}^n \varepsilon_i B_\xi\left(\boldsymbol{p}_i\right)\sigma\left(\boldsymbol{p}_i\right)\right]_{\xi\in\mathcal{M}}.$$

Let $\tilde{s}_{\rho_n,\varepsilon}\left(\boldsymbol{p}\right) = \mathbf{B}(\boldsymbol{p})^\top \tilde{\mathbf{c}}_{\rho_n,\varepsilon}$, where

$$\tilde{\mathbf{c}}_{\rho_n,\varepsilon} = \left[\|B_\xi\|_{L^2(\Omega)}^{-2}\frac{1}{n}\sum_{i=1}^n \varepsilon_i B_\xi\left(\boldsymbol{p}_i\right)\sigma\left(\boldsymbol{p}_i\right)\right]_{\xi\in\mathcal{M}}.$$

For any $\boldsymbol{p} \in \Omega$,

$$\left|s_{\rho_n,\epsilon}\left(\boldsymbol{p}\right) - \tilde{s}_{\rho_n,\varepsilon}\left(\boldsymbol{p}\right)\right| \le \left|\tilde{s}_{\rho_n,\varepsilon}\left(\boldsymbol{p}\right)\right| \max_{\xi\in\mathcal{M}}\left|\|B_\xi\|_{L^2(\Omega)}^2/\|B_\xi\|_{n,\Omega}^2 - 1\right|.$$

According to Lemma 3, we have

$$\max_{\xi\in\mathcal{M}}\left|\frac{\|B_\xi\|_{L^2(\Omega)}^2}{\|B_\xi\|_{n,\Omega}^2} - 1\right| \le \frac{R_n}{1-R_n} = O_P\left\{\sqrt{N\log n/n}\right\}.$$

Thus,

$$\left|s_{\rho_n,\epsilon}\left(\boldsymbol{p}\right) - \tilde{s}_{\rho_n,\varepsilon}\left(\boldsymbol{p}\right)\right| \le O_P\left\{\left(N\log n/n\right)^{1/2}\left|\tilde{s}_{\rho_n,\varepsilon}\left(\boldsymbol{p}\right)\right|\right\}.$$

Hence, finding the asymptotic variance of $\widehat{s}_{\rho_n,\varepsilon}(\boldsymbol{p})$ is equivalent to finding the asymptotic variance of $\tilde{s}_{\rho_n,\varepsilon}(\boldsymbol{p})$. Next we calculate the pointwise variance of $\tilde{s}_{\rho_n,\varepsilon}(\boldsymbol{p})$. Note that

$$\begin{aligned}\mathrm{Var}\left\{\tilde{s}_{\rho_n,\varepsilon}\left(\boldsymbol{p}\right)\right\} &= \mathbf{B}(\boldsymbol{p})^\top \mathrm{E}\left(\tilde{\mathbf{c}}_{\rho_n,\varepsilon}\tilde{\mathbf{c}}_{\rho_n,\varepsilon}^\top\right)\mathbf{B}(\boldsymbol{p}) \\ &= \sum_{\xi\in\mathcal{M}} B_\xi^2\left(\boldsymbol{p}\right)\mathrm{E}\left\{\frac{1}{n}\sum_{i=1}^n \varepsilon_i B_\xi\left(\boldsymbol{p}_i\right)\sigma\left(\boldsymbol{p}_i\right)\|B_\xi\|_{L^2(\Omega)}^{-2}\right\}^2.\end{aligned}$$

For any $\boldsymbol{p} \in \Omega$, let $T_{\xi(\boldsymbol{p})}$ be the tetrahedron that contains $\boldsymbol{p}$. It is easy to see that

$$\|B_\xi\|^2_{L^2(\Omega)} = \int_\Omega B_\xi^2(\boldsymbol{p}) f(\boldsymbol{p}) \mathrm{d}\boldsymbol{p} = \int_{T_{\xi(\boldsymbol{p})}} f(\boldsymbol{p}) \mathrm{d}\boldsymbol{p}.$$

For any continuous function g, let $\omega(g, \varrho) = \sup_{\boldsymbol{p},\boldsymbol{p}' \in \Omega, \|\boldsymbol{p}-\boldsymbol{p}'\| \le \varrho} |g(\boldsymbol{p}) - g(\boldsymbol{p}')|$ be the moduli of continuity of g on Ω. Then for any $\boldsymbol{p}' \in T_{\xi(\boldsymbol{p})}$, we can write $\{f(\boldsymbol{p}') - \omega(f, d_T)\}V_T \le \|B_\xi\|^2_{L^2(\Omega)} \le \{f(\boldsymbol{p}') + \omega(f, d_T)\}V_T$ with d_T being the diameter of the smallest ball containing T. Next, we have

$$\begin{aligned} n\mathrm{Var}\{\tilde{s}_{\rho_n,\varepsilon}(\boldsymbol{p})\} &= n \sum_{\xi \in \mathcal{M}} \|B_\xi\|^{-4}_{L^2(\Omega)} B_\xi^2(\boldsymbol{p}) \mathrm{E}\left\{\frac{1}{n}\sum_{i=1}^n \varepsilon_i B_\xi(\boldsymbol{p}_i)\sigma(\boldsymbol{p}_i)\right\}^2 \\ &= \sum_{\xi \in \mathcal{M}} \|B_\xi\|^{-4}_{L^2(\Omega)} B_\xi^2(\boldsymbol{p}) \mathrm{E}\left\{B_\xi^2(\boldsymbol{p})\sigma^2(\boldsymbol{p})\right\} \\ &= \sum_{\xi \in \mathcal{M}} \|B_\xi\|^{-4}_{L^2(\Omega)} B_\xi^2(\boldsymbol{p}) \int_\Omega B_\xi^2(\boldsymbol{p}')\sigma^2(\boldsymbol{p}') f(\boldsymbol{p}') \mathrm{d}\boldsymbol{p}'. \end{aligned}$$

By the continuity of functions $\sigma^2(\boldsymbol{p})$ and $f(\boldsymbol{p})$, we have

$$\begin{aligned} \mathrm{Var}\{\tilde{s}_{\rho_n,\varepsilon}(\boldsymbol{p})\} &= \frac{1}{n}\sum_{\xi \in \mathcal{M}} \|B_\xi\|^{-4}_{L^2(\Omega)} B_\xi^2(\boldsymbol{p}) \\ &\quad \times \left[\sigma^2(\boldsymbol{p}) f(\boldsymbol{p}) V_T + \int_{T_{\xi(\boldsymbol{p})}} \left\{\sigma^2(\boldsymbol{p}') f(\boldsymbol{p}') - \sigma^2(\boldsymbol{p}) f(\boldsymbol{p})\right\} \mathrm{d}\boldsymbol{p}'\right] \\ &\le \frac{1}{n}\frac{\left\{\sigma^2(\boldsymbol{p}) f(\boldsymbol{p}) + \omega(\sigma^2 f, |T|)\right\} V_T}{\left[\left\{f(\boldsymbol{p}) - \omega(f, |T|)\right\} V_T\right]^2} = \frac{1}{n}\frac{\sigma^2(\boldsymbol{p})}{f(\boldsymbol{v}) V_T}\{1 + o(1)\}. \end{aligned}$$

The conclusion follows. ■

B.4 Proof of Theorem 12

Proof of Theorem 12. To prove Theorem 12, we first show that under Assumptions (A1)—(A4),

$$\frac{s_{\rho_n,\epsilon}(\boldsymbol{p})}{\sqrt{\mathrm{Var}\{s_{\rho_n,\epsilon}(\boldsymbol{p})\,|\mathbb{P}\}}} \xrightarrow{D} N(0,1), \quad n \to \infty. \tag{B.21}$$

Let $\{B_\xi\}_{\xi \in \mathcal{M}}$ be the constructed spline basis functions for $\mathcal{S}$, where $\mathcal{M}$ stands for the index set for spline bases. Note that $s_{\rho_n,\epsilon}(\boldsymbol{p}) = \mathbf{B}(\boldsymbol{p})^\top \widehat{\mathbf{c}}_{\rho_n,\varepsilon}$, where $\widehat{\mathbf{c}}_{\rho_n,\varepsilon}$ is the coefficient vector for $s_{\rho_n,\epsilon}$ with basis functions B_ξ, $\xi \in \mathcal{M}$ and $\mathbf{B}(\boldsymbol{p}) = [B_\xi(\boldsymbol{p}), \xi \in \mathcal{M}]^\top$ is the vector of basis functions. Then, we have

$$\widehat{\mathbf{c}}_{\rho_n,\varepsilon} = \left[\sum_{i=1}^n B_\xi(\boldsymbol{p}_i) B_\zeta(\boldsymbol{p}_i) + \rho_n \langle B_\xi, B_\zeta\rangle_{\mathcal{E}}\right]^{-1}_{\xi,\zeta \in \mathcal{M}} \left[\sum_{i=1}^n B_\xi(\boldsymbol{p}_i)\sigma(\boldsymbol{p}_i)\varepsilon_i\right]_{\xi \in \mathcal{M}}.$$

Recall the definition of $\mathbf{\Gamma}_{\rho_n}$ in Equation B.13, then we have

$$s_{\rho_n,\epsilon}(\boldsymbol{p}) = \mathbf{B}(\boldsymbol{p})^\top \mathbf{\Gamma}_{\rho_n}^{-1} \left[\frac{1}{n}\sum_{i=1}^{n} B_\xi(\boldsymbol{p}_i)\,\sigma(\boldsymbol{p}_i)\,\varepsilon_i\right]_{\xi\in\mathcal{M}} = \frac{1}{n}\sum_{i=1}^{n} \mathbf{B}(\boldsymbol{p})^\top \mathbf{\Gamma}_{\rho_n}^{-1}\mathbf{B}(\boldsymbol{p}_i)\sigma(\boldsymbol{p}_i)\,\varepsilon_i.$$

Let $a_i = n^{-1}\mathbf{B}(\boldsymbol{p})^\top \mathbf{\Gamma}_{\rho_n}^{-1}\mathbf{B}(\boldsymbol{p}_i)\sigma(\boldsymbol{p}_i)$. Obviously, $s_{\rho_n,\epsilon}(\boldsymbol{p}) = \sum_{i=1}^n a_i\varepsilon_i$, and

$$a_i^2 = n^{-2}\mathbf{B}(\boldsymbol{p})^\top \mathbf{\Gamma}_{\rho_n}^{-1}\mathbf{B}(\boldsymbol{p}_i)\mathbf{B}(\boldsymbol{p}_i)^\top \mathbf{\Gamma}_{\rho_n}^{-1}\mathbf{B}(\boldsymbol{p})\sigma^2(\boldsymbol{p}_i).$$

For any vector $\mathbf{c} = (c_\xi, \xi \in \mathcal{M})^T$, we have

$$\mathbf{c}^\top \mathbf{B}(\boldsymbol{p}_i)\mathbf{B}(\boldsymbol{p}_i)^\top \mathbf{c} = \left\{\sum_{\xi\in\mathcal{M}} c_\xi B_\xi(\boldsymbol{p}_i)\right\}^2 \le \|\mathbf{c}\|_2^2 \sum_{\xi\in\mathcal{M}} B_\xi^2(\boldsymbol{p}_i),$$

which means the maximal eigenvalue of the matrix $\mathbf{B}(\boldsymbol{p}_i)\mathbf{B}(\boldsymbol{p}_i)^\top$ is bounded by $\sum_{\xi\in\mathcal{M}} B_\xi^2(\boldsymbol{p}_i)$. Consequently, according to the proof of Theorem B.5 in Section B.3, with probability approaching one,

$$\begin{aligned} a_i^2 &\le n^{-2}\sum_{\xi\in\mathcal{M}} B_\xi^2(\boldsymbol{p}_i)\mathbf{B}(\boldsymbol{p})^\top \mathbf{\Gamma}_{\rho_n}^{-1}\mathbf{\Gamma}_{\rho_n}^{-1}\mathbf{B}(\boldsymbol{p})\sigma^2(\boldsymbol{p}_i) \\ &\le \frac{C_\sigma^2}{n^2|\triangle|^6}\sum_{\xi\in\mathcal{M}} B_\xi^2(\boldsymbol{p}_i)\mathbf{B}(\boldsymbol{p})^\top\mathbf{B}(\boldsymbol{p}) = \frac{C_\sigma^2}{n^2|\triangle|^6}\sum_{\xi\in\mathcal{M}} B_\xi^2(\boldsymbol{p})\sum_{\xi\in\mathcal{M}} B_\xi^2(\boldsymbol{p}_i). \end{aligned}$$

On the other hand, $\sum a_i^2 = \mathrm{Var}\{s_{\rho_n,\epsilon}(\boldsymbol{p})|\mathbb{P}\}$. Using similar arguments in the proof of Theorem B.5, for $\rho_n = 0$, we have $\sum a_i^2 \ge c_\sigma^2 n^{-1}|\triangle|^{-3}\sum_{\xi\in\mathcal{M}} B_\xi^2(\boldsymbol{p})$ with probability approaching one. Therefore, there exists some positive constant C, such that

$$\frac{\max_{1\le i\le n} a_i^2}{\sum a_i^2} \le \frac{CC_\sigma^2}{n|\triangle|^3 c_\sigma^2}\sum_{\xi\in\mathcal{M}} B_\xi^2(\boldsymbol{p}_i) = O_P\left(Nn^{-1}\right) = o_P(1).$$

Hence, $\sum a_i\varepsilon_i/\left(\sum a_i^2\right)^{1/2} \to N(0,1)$ by Linderberg-Feller CLT. Similarly for $\rho_n > 0$, we have with probability approaching one,

$$\sum a_i^2 \ge \frac{c_\sigma^2}{n(1+n^{-1}|\triangle|^{-4}\rho_n)^2|\triangle|^3}\sum_{\xi\in\mathcal{M}} B_\xi^2(\boldsymbol{p}),$$

and

$$\frac{\max_{1\le i\le n} a_i^2}{\sum a_i^2} \le \frac{CC_\sigma^2\left(1+n^{-1}|\triangle|^{-4}\rho_n\right)^2}{n|\triangle|^3 c_\sigma^2}\sum_{\xi\in\mathcal{M}} B_\xi^2(\boldsymbol{p}_i) = O_P\left(Nn^{-1}\right) = o_P(1),$$

which gives Equation B.21.

Note that under Assumptions (A3), (A4') and (A5), the bias term in Equation 12 is negligible compared to the order of $[\mathrm{Var}\{s_{\rho_n,\epsilon}(\boldsymbol{p})|\mathbb{P}\}]^{1/2}$ given in Theorem B.5 in the supplementary material (Section B). Thus, Theorem 12 follows directly from Equation B.21. ■
